# Game-based learning in microbiology education: design and classroom use of MicroBioCombat

**DOI:** 10.1099/acmi.0.001238.v3

**Published:** 2026-08-07

**Authors:** Raul Vinicius Salata Souto, Edmara Rocha dos Santos Moreira, Leandro Marcio Moreira

**Affiliations:** 1 Mestrado Profissional em Ensino de Ciências - MPEC, Universidade Federal de Ouro Preto, Campus Morro do Cruzeiro, Ouro Preto, MG, Brazil; 2 Departamento de Ciências Biológicas - DECBI, Instituto de Ciências Exatas e Biológicas, Universidade Federal de Ouro Preto, Campus Morro do Cruzeiro, Ouro Preto, MG, Brazil

**Keywords:** active learning, biology teaching, educational games, gamification, microbiology education, secondary education

## Abstract

Microbiology is often perceived by students as a challenging subject, particularly when addressing pathogenic mechanisms and disease transmission. Active learning approaches, including educational games, have been shown to enhance student engagement and conceptual understanding by integrating playful elements with scientific content. In this study, we present *MicroBioCombat*, a card-based educational game developed for use in secondary school microbiology education. The game comprises 81 cards representing pathogenic bacteria, transmission routes and prophylactic or therapeutic strategies. During gameplay, students combine cards to simulate infection scenarios and corresponding defence mechanisms, with the aim of reducing their opponent’s score to zero. Beyond reinforcing microbiological concepts, *MicroBioCombat* promotes peer interaction, communication and decision-making, contributing to a more dynamic and inclusive classroom environment. The game is adaptable to different educational levels and curricular contexts, and an accompanying online forum enables educators and students to share experiences and gameplay strategies, supporting collaborative and reflective learning.

## Graphical abstract

Didactic intervention proposal via *MicroBioCombat*. The gamification tool is presented as a cognitive mediator, facilitating the internalization of complex knowledge regarding pathogens and sanitary control strategies through playful practice.

**Impact Statement**

- Microbiology education in secondary schools is challenged by conceptual complexity and limited laboratory infrastructure, often reducing student engagement with infectious disease topics. This study presents *MicroBioCombat*, a low-cost, card-based educational game that integrates pathogen biology, transmission routes, immune responses and preventive strategies into an interactive learning experience.
- Quantitative learning gains and qualitative analyses of student dialogue demonstrate significant improvements in conceptual integration and scientific reasoning, moving students beyond memorization toward systems-level understanding.
- Adaptable and scalable, MicroBioCombat offers an accessible pedagogical model for resource-limited educational contexts. Given the global importance of infectious disease awareness and antimicrobial resistance literacy, this work contributes evidence that structured gamification can meaningfully strengthen microbiology education and early scientific literacy.

**Outcomes**

Upon completion of the *MicroBioCombat* intervention, students are expected to:

- Identify and differentiate between key pathogenic bacterial genera and their specific modes of transmission.
- Explain the physiological mechanisms of bacterial infection and the role of host defence systems, including innate and adaptive immunity.
- Apply strategic reasoning to correlate clinical manifestations with appropriate preventive or therapeutic interventions.
- Demonstrate the ability to integrate multidisciplinary concepts (microbiology, immunology and epidemiology) through systemic analysis.

## Data Summary

All data associated with this work is reported within the article and supplementary files.

## Introduction

The relevance of microbiology extends far beyond the boundaries of the classroom, as micro-organisms are integral to everyday life through the diverse interactions between humans and microbial communities across multiple environments and ecological niches [1]. These interactions are complex and play a fundamental role in maintaining ecosystem equilibrium. However, anthropogenic activities and rapid population growth have increasingly disrupted these balances, contributing to the emergence and spread of human diseases [2].

Although the incidence of bacterial diseases such as syphilis, tuberculosis and Hansen’s disease has declined due to preventive campaigns, vaccination programmes and the widespread use of antibiotics, the past decade has seen the re-emergence of other infectious pathologies [3]. This resurgence is largely associated with the increasing prevalence of antibiotic resistance, often resulting from the inappropriate or excessive use of antimicrobial drugs [4]. Resistant strains, such as vancomycin-resistant *Staphylococcus aureus*, represent major medical, political and social challenges in an increasingly globalized world [5].

Preventive measures are therefore essential to reduce infections caused by multidrug-resistant micro-organisms [6]. The success of such measures is closely linked to the effective dissemination of scientific knowledge and health education initiatives [7]. Evidence indicates that high-quality educational programmes significantly improve public awareness and the adoption of preventive behaviours [8].

Despite this, traditional teaching approaches frequently limit student engagement and learning outcomes, particularly in under-resourced schools that lack laboratory infrastructure and costly equipment [9]. In response to these challenges, dynamic and interactive teaching strategies, such as digital tools and educational games, have been increasingly adopted worldwide [10]. Within secondary education, including both lower- and upper-secondary school curricula, active learning methodologies are essential to shift students from passive listeners to active participants [11]. Gamification, by integrating structured mechanics like rules, goals and strategic choices, serves as a powerful pedagogical bridge for school-aged learners. It has been widely documented to increase emotional engagement, sustain motivation and foster collaborative problem-solving [12]. By translating abstract, microscopic phenomena into visible, interactive dynamics, educational games help adolescent students contextualize scientific knowledge within their reality, overcoming the limitations of memorization-based teaching [13]. Given its conceptual complexity and relevance to real-world issues, medical microbiology is particularly well suited to gamified learning approaches [14].

Didactic games have been shown to enhance learning by diversifying instructional strategies while promoting creativity, social interaction, problem-solving, leadership and mutual respect among students [15]. When effectively implemented, such tools capture learners’ attention, simulate real-life scenarios and encourage healthy competition, thereby fostering deeper engagement with scientific content [16].

Inspired by the popular game *Magic: The Gathering™*, we developed MicroBioCombat, a card-based educational game designed for secondary school students that simulates biological conflicts. Players use cards representing pathogenic bacteria, transmission mechanisms and prophylactic or therapeutic strategies to attack opponents and defend against infections. Through strategic decision-making and gameplay, the activity reinforces key microbiological concepts while providing an engaging and playful learning environment.

## Methods

### Study design and research characterization

This study is characterized as qualitative and descriptive research with a focus on pedagogical intervention [17]. It aimed to evaluate the effectiveness of the educational game *MicroBioCombat* as a didactic tool for teaching microbiology and pathologies associated with the Domain Bacteria and Domain Archaea, emphasizing student engagement and the application of scientific concepts through ludic strategies.

### Research setting and subjects

The study was conducted in a public high school operating under the standard Brazilian education curriculum in the state of Minas Gerais, Brazil. The participants were 36 students aged 14–16 years, including 22 female students. The MicroBioCombat game proposal was incorporated into the regular curriculum and classroom planning, ensuring that the activity complemented the standard teaching objectives for microbiology and biology. All procedures followed ethical guidelines for educational research and were carried out with the support of the school’s pedagogical coordination. The school provided a regular classroom environment without specialized laboratory facilities, reflecting the typical conditions of public education in the region.

### Preparation and pedagogical intervention

The pedagogical intervention was structured in three sequential phases:

- Phase 1 (theoretical preparation). Involved theoretical preparation and contextualization, mediated through expository and dialogical lectures [18], during which the teacher introduced core concepts related to the Domain Bacteria and Domain Archaea, bacterial morphology and physiology and the distinction between commensal and pathogenic relationships. Mechanisms of bacterial infection, including antibiotic resistance and host defence systems, were also discussed. Following these lectures, a diagnostic problematization activity was applied to establish the students’ baseline understanding, representing the conceptual grasp achieved after standard instruction, prior to the gamified intervention [19].
- Phase 2 (gameplay intervention). The second phase consisted of the application of the game *MicroBioCombat*, presented as a biological battle inspired by *Magic: The Gathering™*. The rules and card categories (pathogenic bacteria, defence strategies and conveyance or fostering mechanisms) were explained by the teacher. Students were organized into pairs or small groups [20] and provided with card decks and a ‘Vital Energy Table’ to record health damage during the rounds (see details below). Each player began with 20 life points and used dice-generated energy points to activate card combinations representing biological interactions.
- Phase 3 (knowledge consolidation). Focused on post-game synthesis and knowledge consolidation. During gameplay, the teacher acted as a mediator, clarifying doubts and reinforcing scientific concepts [21]. After the activity, a second diagnostic problematization was applied to evaluate changes in students’ technical reasoning and conceptual integration [22]. As a concluding activity, students collaboratively developed concept maps linking pathogens, transmission routes, clinical manifestations and prevention strategies [23].

### Data collection and analysis

To ensure clarity regarding the assessment of MicroBioCombat, all evaluation procedures are detailed herein:

(1) Pre- and post-diagnostic evaluations [24]. Students completed written problem-solving tasks before and after the gameplay. These tasks included hypothetical scenarios requiring identification of pathogens, vectors, affected organs, defence mechanisms and preventive strategies. Responses were scored for accuracy, completeness and conceptual integration, allowing a quantitative comparison of pre- and post-game performance. (2) Participant observation and analysis of scientific dialogue [25]. The teacher observed students in real time during gameplay, recording interactions, strategic decision-making and card usage. Dialogues between students, and between students and the teacher, were transcribed and analysed to identify the real-time application of microbiology concepts. (3) Content analysis of written and oral responses [26]. Both the diagnostic written tasks and recorded oral dialogues (including the structural design of student-generated concept maps) were subjected to qualitative analysis. Responses were coded according to predefined categories (e.g. pathogen identification, transmission mechanism, host response and preventive measures), allowing the tracking identification of patterns of conceptual growth and student engagement across the study population.

Written pre- and post-test assessments provided the quantitative data for Table 1, representing the proportion of the cohort that correctly addressed each microbiological concept. Qualitative insights from scientific dialogue and oral responses were used exclusively for thematic validation and were not included in these statistical calculations.

**Table 1.** Comparative analysis of student performance before and after the gamified intervention (*N*=36)

Concept category	Subitem/indicator	Pre-game (% correct)	Post-game (% correct)	Normalized gain (*g*)	Statistical significance (*χ*^2^, df=1)	***P*-value**	Observation/impact of gameplay
**Pathogen recognition**	Correct identification of bacteria	40	85	0.75	13.06	<0.001	Most students learned to correctly identify pathogens based on card and symptoms
	Association with cell type (Gram-positive/Gram-negative, aerobic/anaerobic)	25	75	0.67	14.44	<0.001	Students differentiated essential microbiological characteristics
**Transmission/vectors**	Recognition of vectors/transmission mechanisms	30	80	0.71	14.45	<0.001	Students correctly associated vectors such as rats, fleas and contaminated water
	Association pathogen+vector+disease	20	70	0.63	14.49	<0.001	Demonstrates integration of pathogen and transmission route concepts
**Defence/immunity**	Identification of immune cells (neutrophils, macrophages)	20	75	0.69	16.33	<0.001	Improved understanding of immune system function
	Application of defence strategies using cards	15	70	0.65	16.41	<0.001	Students correctly applied cards such as vitamin C, neutrophils and vaccines
**Preventive measures**	Recognition of preventive strategies	10	65	0.61	16.53	<0.001	Includes hygiene, vaccination, food and sexual safety measures
	Planning defence by combining cards	5	60	0.58	16.68	<0.001	Demonstrates strategic thinking and integrated reasoning
**Conceptual integration**	Ability to relate pathogen, vector and defence	15	75	0.71	18.27	<0.001	Gameplay promoted integration of microbiology, ecology and health knowledge
	Ability to reason causality (why disease occurred)	10	70	0.67	18.42	<0.001	Students elaborated explanations consistent with biological concepts
**Response complexity**	Complete and detailed responses in tests	10	65	0.61	16.53	<0.001	Gameplay increased the conceptual depth of students’ answers
	Application of knowledge to hypothetical scenarios	5	60	0.58	16.68	<0.001	Demonstrates transfer of learning to practical situations

Percentage of students (*n*=36) demonstrating proficiency in microbiological concepts based on structured written assessments. The values represent the proportion of the cohort that provided correct answers for each evaluated indicator. The normalized gain (*g*) was calculated according to Hake’s method, where gains are qualitatively categorized as low (*g*<0.30), medium (0.30≤*g*<0.70) or high (*g*≥0.70). Statistical significance for the change in cohort proportions from pre- to post-game was assessed individually for each indicator using McNemar’s chi-square (*χ*2) test with 1 degree of freedom (df=1) based on absolute cohort counts.

### Game architecture and mechanics

**Fig. 1.**
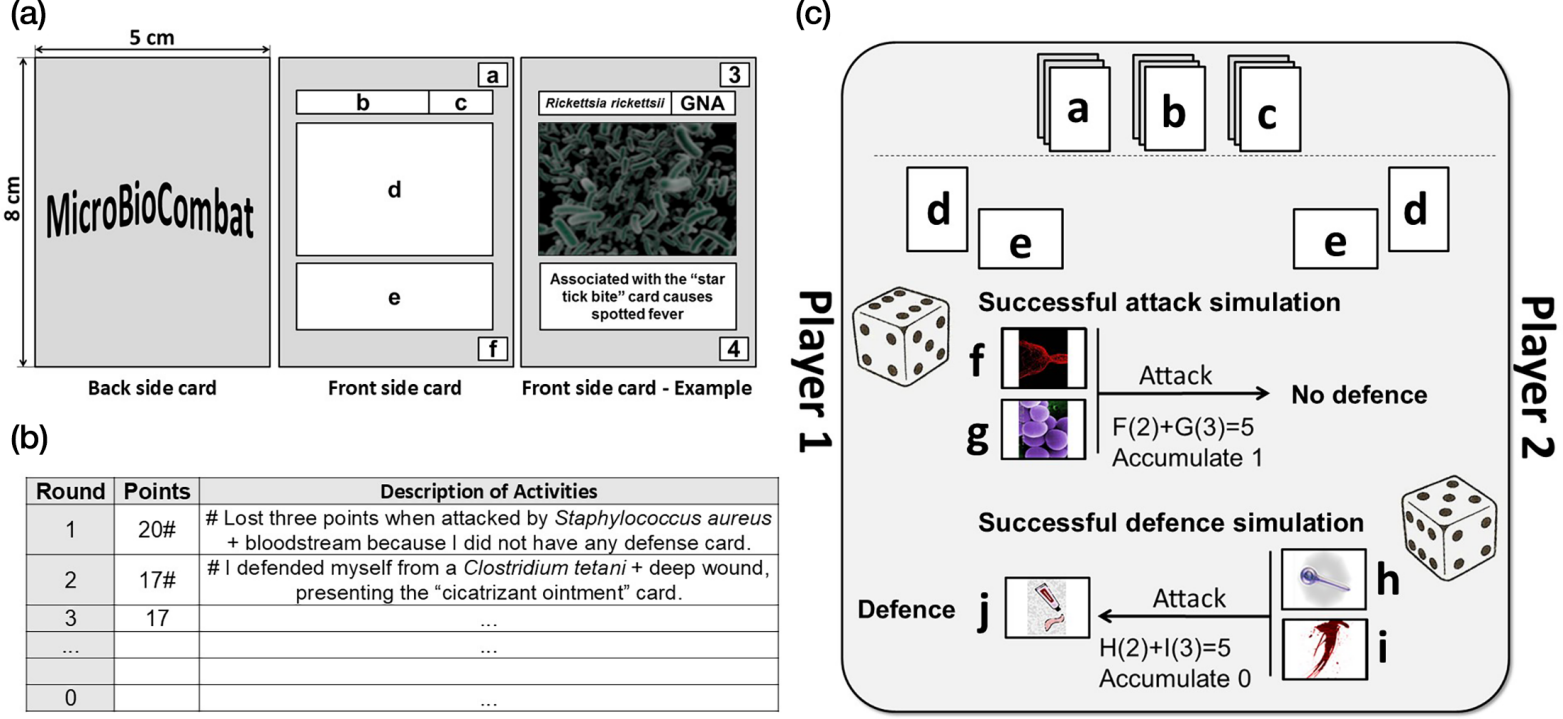
Structure, composition and game dynamics of *MicroBioCombat*. (**A**) Structure and composition of the cards, shown from left to right. Back-side card model; front-side information: a – vital energy required to activate the card (numeric, 1–5 digits); b – scientific name of the bacterium, defence mechanism or conveyance mechanism, including associated advantages (alphanumeric, up to 40 characters); c – bacterial cell structure and lifestyle (alphanumeric, up to 45 characters); d – illustration of the bacterium, environment or conveyance mechanism (image, any format, 4×4 cm); e – technical and additional information (alphanumeric, up to 330 characters); f – damage points inflicted on the opponent by the card (numeric, 1–5 digits). An example of a card created according to these specifications is shown. (**B**) Example of a ‘vital energy’ scoring table, which is essential for calculating the points obtained during gameplay. (**C**) Example of a game configuration: a–c, – set of pathogen, vector and conveyance cards, placed face down; d, e – attack and defence card sets of each player; f, g – simulation of a successful attack performed by player 1, in which the ‘bloodstream’ and *S. aureus* cards are played together against the opponent, who does not possess a compatible defence card; h–I – attack performed by player 2 using the *Clostridium tetani* and ‘deep wound’ cards, which is countered by a successful defence. The upper panel illustrates a successful attack, whereas the lower panel depicts a successful defence.

*MicroBioCombat* was originally structured to hold 81 cards whose dimensions and characteristics are presented in Fig. 1(a). These cards are grouped into three distinct, colour-coded categories with different colours: pathogenic bacteria (yellow, 26 cards – Table S1, available in the online Supplementary Material), defence strategies (blue, 33 cards – Table S2) and mechanisms of conveyance and aiding of pathogens (purple, 22 cards – Table S3). A version of these cards for printing is available in File S1.

Moreover, a die and a table to record energy points (Vital Energy Table – File S2) are also part of the game (Fig. 1b). The filling of this table is necessary for the discussion of the possible mechanisms of attack and defence proposed during the combats. The objective of the game is to use the cards of pathogenic bacteria to cause ‘health damage’ to the adversary until their points of life are reduced to zero. Each player starts with 20 points (or more, according to the players’ criteria), depending on the time available. The game can be played individually or in pairs. A table, which serves as the base for the board and battlefield, is placed between the adversaries, where cards are placed at an extremity of the table (Fig. 1b). In the absence of a table, the game can be played on the floor, as long as the space for the battle is defined.

### Rules of the gameplay dynamics

Before the beginning, each player or pair chooses seven cards from any group (Fig. 1c), provided the hand does not exceed seven cards. One of the players/pairs starts by throwing a die to obtain the points of energy. The value obtained is used to activate the cards held in the player’s hand, according to the energy cost indicated in the upper right corner of each card. This value activates as many cards as necessary, provided there is enough energy (see examples below).

The activated cards inflict damage on the adversary, and the attacks must be carried out by the participant who previously threw the die. This attack can be repelled if the defending player, in the same round, throws the die and obtains the points necessary to activate the specific defence card. Both for attack and defence purposes, the points obtained from the dice are cumulative; if players do not exhaust their points during a turn, the remainder can be saved for the following round. If the attacker causes damage, the defender will have those points deducted from their vital energy pool and all modifications must be recorded in the table of vital energy (Fig. 1c). At the end of each round (encompassing the attack and defence of each player), players can change one of their cards for any other from the piles, as long as the discarded card is returned to the bottom of the corresponding deck. The winner is the participant who reduces the vital points of the adversary to zero. A teacher/tutor support version containing the complete game rules can be found in File S3 for printing,

### Core combat simulations (attack and defence examples)

Here are two simulations that exemplify the game dynamics.

#### Simulation 1: successful attack

This combination simulates an attack on the adversary using the card of the bacterium *S. aureus.* To be successful, the player must simultaneously play the card *bloodstream*, which represents the conveyor of the bacterium (Fig. 1c). Players must understand that if this bacterium enters the bloodstream of the patient, it can cause systemic damage to organs and tissues. While there is no energy cost to activate the *bloodstream* card, the *S. aureus* card requires three energy points. This means that the player can only execute this attack when values equal to or higher than three are reached in the die (or compiled from previous rounds). In the absence of a compatible defensive mechanism from the opponent, the player being attacked will lose three life points.

#### Simulation 2: successful defence

An opponent initiates an attack combining the *Clostridium tetani* card with the card that characterizes ‘deep wound’, requiring a total activation cost of five energy points (3+2, respectively), achieved through a high die throw or cumulative energy points (Fig. 1c). When this combination is presented in the battlefield in an attempt to cause damage to the opponent, the latter can counter it by playing the aerobic *environments+cicatrizant ointment* cards, which require only one energy point (a value higher than one in the die). In a biological context, hydrogen peroxide/oxygen exposure limits the reproduction of the bacterium due to its obligate anaerobic physiology, and the ointment accelerates healing. This combination precludes the survival of the bacterium in the environment, preventing the development of tetanus. Another option would be the use of the DPT vaccine card to successfully counter this specific attack.

### Statistical data analysis

To evaluate the educational effectiveness of the MicroBioCombat intervention, student performance was assessed across 12 specific conceptual and behavioural indicators grouped into 5 core microbiological categories. Because data collection captured cohort-wide nominal outcomes (correct vs. incorrect responses) tracked pre- and post-game for the same group of students, a non-parametric approach for dependent proportions was employed.

To quantify the magnitude of conceptual progression, Hake’s normalized gain (*g*) [27] was calculated for each indicator using the standard formula: *g* = (%Post-Game−%Pre-Game)/100−%Pre-Game. The achieved learning gains were qualitatively interpreted using Hake’s boundaries: low gain (*g*<0.30), medium gain (0.30≤*g*<0.70) and high gain (*g*≥0.70).

To determine whether the changes in correct response proportions from pre- to post-game within the cohort (*N*=36) were statistically significant, McNemar’s chi-square (*χ*^2^) tests [28] for paired nominal data, incorporating Yates’s continuity correction [29], were performed individually for each of the 12 indicators. This analysis explicitly accounts for the dependent/dependent nature of repeated measurements obtained from the same subjects. Percentages were converted back to absolute student counts to populate 2×2 correlated contingency tables for each analytical item. All McNemar tests were evaluated with 1 degree of freedom (df=1), assuming an a priori significance level of *α*=0.05. Statistical computations were executed using R software v4.3.

### Printable materials for implementation

In order to share this pedagogical tool, the developed cards are available for printing in Supplementary File 1. Along with the cards, the general rules (File S3) and the scoring table (File S2) are also being made available.

## Results

### Learning outcomes and game dynamics overview

*MicroBioCombat* facilitated student learning in bacterial pathologies. The dynamic gameplay and diverse card scenarios initially revealed students’ gaps in basic microbiology knowledge. Over successive rounds, students increasingly applied concepts correctly, demonstrating improved reasoning and conceptual integration. Observation of interactions and informal discussions confirmed that the game supported learning effectively.

### Card composition and structural architecture

The MicroBioCombat game was developed with 81 cards, organized into 3 distinct, colour-coded categories: pathogens, vectors/fostering mechanisms and defence or protection strategies. The set of pathogen and vector cards includes clinically relevant bacteria, their biological characteristics and associated diseases, allowing students to explore modes of transmission and ecological interactions in a controlled, gamified context (Table S1). By engaging with these cards, students can relate theoretical microbiology concepts to concrete, hypothetical scenarios, reinforcing conceptual understanding and critical thinking.

Complementing this, the defence and protection cards introduce environmental interventions, antibiotics, vaccines, immune cells, behavioural strategies and other mechanisms that modulate bacterial attack or host defence (Tables S2 and S3). These cards provide opportunities for students to experiment with strategies, observe outcomes and relate them to biological principles, fostering a dynamic learning environment. Together, the three sets of cards integrate microbiology, disease transmission, immune responses and prevention, offering a multidisciplinary and interactive platform that promotes both conceptual understanding and practical reasoning in the classroom.

### Pre- and post-game diagnostic assessments

To systematically evaluate the cognitive gains of the intervention, student performance was tracked using a pre- and post-test design, administered immediately following the lecture sessions and again after the gameplay (*N*=36). These scores across 12 targeted indicators were converted back to absolute student frequencies to apply non-parametric repeated-measures analyses, as described in the statistical framework (Table 1).

The qualitative shifts observed in the diagnostic instruments echo these highly significant statistical trends. For instance, in a pre-game scenario describing a child who died after an autopsied discovery of fibrin accumulation in the throat, students consistently exhibited a diagnostic breakdown, misidentifying the condition as a simple cold without linking fibrin formation to an active bacterial infection. Conversely, in post-game assessments featuring a clinical presentation of diarrhoea and vomiting following the consumption of raw eggs, students demonstrated sophisticated technical reasoning. Post-game responses successfully identified *Salmonella* as the responsible genus and precisely mapped its physiological site of action to the digestive system, displaying an enhanced capacity to establish multi-tiered clinical connections.

### Qualitative analysis of student dialogues

Real-time tracking of peer-to-peer interactions and formal transcription of dialogues during the game rounds confirmed that *MicroBioCombat* moved learning beyond passive memorization. Over successive rounds, students minimized trial-and-error card plays and began verbalizing advanced physiological justifications.

Two explicit interaction transcripts (Clashes) highlight this intellectual development:

- Clash A: During an active combat round, an opponent initiated an attack combining a *C. tetani* card with a *deep wound* card. The defending student strategically countered by deploying an *aerobic environments* card alongside a *cicatrizant ointment* card, articulating: ‘*I can’t just use the ointment because the bacterium is already in a deep wound where there’s no oxygen, so I need to change the environment to stop it.*’ This exchange confirms that students successfully mastered the metabolic restrictions of obligate anaerobes to justify tactical defensive moves.
- Clash B: In a separate round, students integrated cell-mediated innate immunity concepts by correctly pairing *neutrophil* and *macrophage* cards during a bloodstream infection simulation. The active dialogue focused on cellular abundance and the kinetic timing of host responses within the vascular network, mapping complex systemic concepts rarely retained in standard high school biology curricula. These interaction transcripts serve as illustrative examples of the cognitive processes that underpinned the statistical gains presented in Table 1. While these specific dialogues highlight advanced reasoning, they represent recurring interaction patterns observed across the classroom, consistent with the 67–75% proficiency increase in identifying pathogenic mechanisms and host immune responses.

### Conceptual integration and engagement analysis

**Fig. 2.**
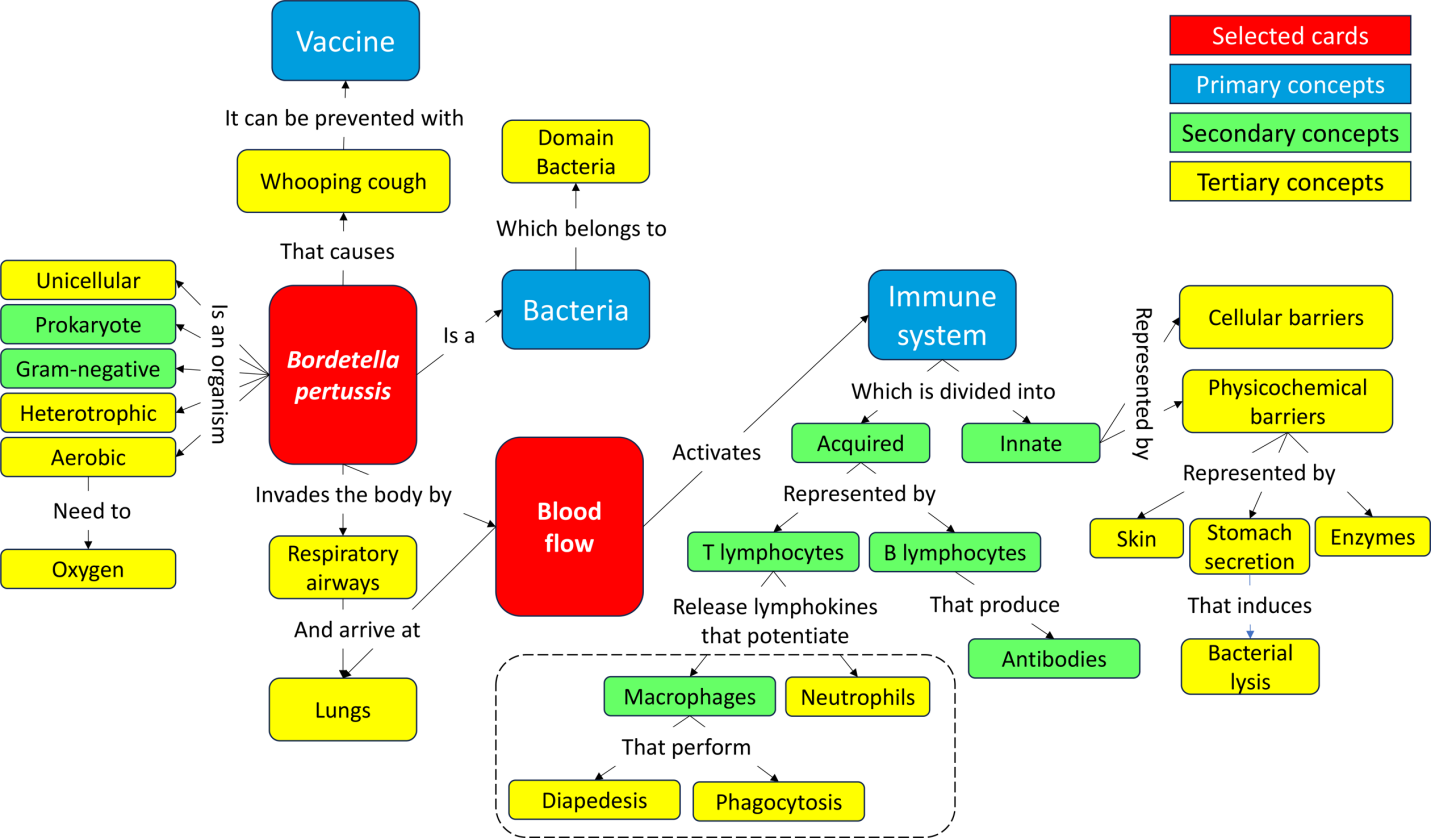
Concept map of *Bordetella pertussis* pathogenesis and immune response. This map organizes biological information through a three-tier hierarchy: primary (blue) for foundational pillars, secondary (green) for systemic subdivisions and tertiary (yellow) for specialized technical details. The dashed perimeter identifies the cluster of concepts related to cellular dynamics that required intensified tutorial intervention to be correctly integrated into the map’s structure.

The analysis of the concept maps produced by the students revealed a high level of technical accuracy, with over 80% of the groups correctly linking the pathogen’s morphological characteristics to its specific transmission route and the corresponding host immune response. (Fig. 2). This concept map is representative of the structural complexity achieved by the majority of the groups; its internal logic directly correlates with the high quantitative gains observed in ‘conceptual integration’ (Table 1), confirming that the depth seen in this example was a widespread outcome across the cohort. The only cluster requiring active tutorial mediation involved fine-scale cellular kinetics, which was successfully resolved during post-game debriefing.

The combination of diagnostic test scores and observed interactions informed the calculation of the indicators presented in Table 1, showing the evolution from pre-game to post-game performance. The qualitative assessment of students’ engagement demonstrated a clear increase in interest and motivation after the use of *MicroBioCombat*. Students actively participated in discussions, proposed new strategies and connected game scenarios to real-life microbiology concepts (Table 2). The collaborative nature of the game enhanced peer learning, and students showed enthusiasm for repeated gameplay, indicating that the tool effectively transforms microbiology into an interactive and stimulating subject.

**Table 2.** Qualitative assessment of student engagement and interest

Indicator	Pre-game observation	Post-game observation	Comment/impact
Participation in class discussions	Low, students reluctant to answer	High, students actively debated strategies	MicroBioCombat stimulated curiosity and encouraged discussion
Motivation to learn microbiology	Moderate, limited engagement	High, students asked for additional examples and strategies	Game made abstract concepts tangible and enjoyable
Initiative in problem-solving	Limited, followed textbook examples	Improved, students proposed alternative strategies and predictions	Promoted strategic thinking and applied learning
Collaboration and teamwork	Variable, some students disengaged	Consistent, paired or group play fostered discussion	Encouraged social learning and peer teaching
Overall enthusiasm	Moderate, passive learning	High, students requested extra rounds and explored card interactions	Increased intrinsic motivation for microbiology
Curiosity and questioning	Few spontaneous questions	Students actively questioned mechanisms and outcomes	Enhanced scientific inquiry and critical thinking
Attention and focus	Frequently distracted	Sustained attention throughout gameplay	Ludicity maintained engagement and minimized off-task behaviour
Strategic thinking	Rarely planned actions	Students developed multi-step strategies using cards	Fostered cognitive flexibility and foresight
Communication skills	Limited verbalization	Students articulated reasoning clearly to peers	Strengthened academic discourse and peer teaching
Confidence in applying knowledge	Low, hesitant to answer	Increased, students linked concepts to game scenarios	Built self-efficacy in microbiology and problem-solving
Creativity	Not observed	Students proposed novel combinations of cards and strategies	Stimulated imaginative approaches to microbiology concepts
Retention of concepts	Moderate, some misconceptions	Improved, students recalled and applied prior knowledge	Reinforced long-term understanding and concept integration

## Discussion

The results of this study demonstrate that *MicroBioCombat* effectively fosters active learning and conceptual understanding in microbiology. These improvements in knowledge retention and scientific dialogue align with Michael [30], reinforcing the role of active learning in translating theoretical frameworks into dynamic, applied scenarios. A key strength of this study lies in the sequential design: the expository phase provided the necessary theoretical scaffolding, while the game served as the mechanism for conceptual consolidation. By assessing students both post-lecture and post-game, we evidenced that gamification significantly enhanced mastery of complex concepts beyond the limits of traditional didactic instruction, establishing it as a potent consolidation strategy rather than a mere introductory tool.

Pre- and post-game assessments revealed a clear progression toward sophisticated reasoning, where students successfully linked pathogens to their sites of action. This progression mirrors Zambito *et al*. [31], who observed similar cognitive gains when using interactive, game-based tools to teach microbiology in health-related contexts. These qualitative improvements align with the high statistical gains observed across all conceptual categories.

Educational games have been increasingly recognized as powerful tools in science education, particularly in microbiology, where abstract concepts and limited laboratory access often challenge students’ engagement and understanding [32, 33]. While these studies primarily associate gamification with improved student affect and motivation, our findings suggest a more profound cognitive impact: the structural gains observed in concept mapping (Fig. 2) demonstrate that MicroBioCombat functions as a cognitive scaffold for systemic reasoning. Gamified approaches provide interactive simulations of real-life microbial events, enabling students to explore hypothetical scenarios, test strategies and observe consequences without the constraints of expensive laboratory resources. Such experiences have been shown to enhance motivation, stimulate discussion and foster critical thinking and problem-solving skills [ 34, 35, 36]. However, unlike these studies, which often rely on self-reported engagement metrics, our results provide quantitative evidence of conceptual mastery (Table 1), showing that gamification can drive specific learning outcomes in microbiology curricula. The effectiveness of MicroBioCombat is particularly relevant when considering the socioeconomic context of the school where this study was conducted. As a public institution lacking specialized laboratory facilities, the game served as a ‘virtual laboratory’, providing a low-cost yet high-impact alternative for practical scientific exploration. By simulating complex biological interactions that would otherwise require expensive equipment or culture media, the game democratized access to experimental reasoning, proving that high-quality microbiology education is achievable even in resource-limited settings.

While *MicroBioCombat* does not replace the tactile and technical expertise inherent to laboratory work, it offers a distinct advantage in modelling complex systems. Although Timmis (2023) [9] argues that technical expertise is better acquired through traditional bench-top practice, and we acknowledge that such manual proficiency remains a foundational pillar of scientific literacy; our study shifts the focus toward ‘minds-on’ systemic modelling for settings where physical practice is unattainable. Whereas traditional secondary-level laboratory exercises are often restricted to isolated techniques, such as Gram staining or microscopic observation, this game allows for the simulation of systemic interactions between pathogens, vectors and immune responses that would be difficult to reproduce in basic school laboratories. Therefore, gamification functions not as a replacement but as a complementary strategy that enables students to visualize biological dynamics at population and epidemiological scales, bridging the gap between conceptual theory and the 'hands-on' experience that resource-limited environments often fail to provide.

*MicroBioCombat* aligns with these pedagogical principles by combining conceptual complexity with playful engagement [37]. The game encourages students to reason strategically about microbial attacks, immune defences and prophylactic measures, integrating multiple disciplines such as microbiology, immunology, ecology and epidemiology. This multidimensional learning approach mirrors the complexity of real-world infectious disease scenarios, allowing students to connect theory with practice [38] and develop transferable skills, including decision-making, collaboration and scientific argumentation. While our analysis primarily focused on conceptual gains (Table 1), the qualitative evidence from student interactions (see Qualitative analysis of student dialogues) provides a window into these transferable skills: the observed ‘Clashes’ exemplify student-led scientific argumentation and collaborative decision-making, where the need to justify moves using microbiological principles directly incentivized critical thinking and consensus-building.

The qualitative analysis of student dialogues further illustrates the value of active discussion-based learning. Students demonstrated a capacity to apply concepts such as the anaerobic requirements of *C. tetani*, the functional differences between neutrophils and macrophages, and the pathogenic potential of otherwise commensal strains of *Escherichia coli*. These insights indicate that the game supports deeper conceptual integration, which is often difficult to achieve in traditional high school classrooms where laboratory exercises are limited [39]. Building upon this qualitative evidence, the alignment of our multi-stage analytical approach with our research questions confirms the multifaceted impact of *MicroBioCombat*. While diagnostic testing provided the ‘what’ (the statistical evidence of conceptual gains), the hypothetical scenarios and concept maps provided the ‘how’, demonstrating the students’ capacity to navigate complex biological systems. Specifically, the high structural density of the concept maps (Fig. 2) directly illustrates the ‘Systemic Interdependence’ theme identified in our content analysis, proving that students are not merely memorizing terms, but successfully integrating microbiology, immunology and epidemiology into a coherent mental framework. This triangulation of quantitative and qualitative data provides a comprehensive validation of the gamified strategy.

Importantly, the engagement promoted by the game also reduces classroom anxiety and encourages social interaction and peer learning, reinforcing positive educational outcomes beyond content acquisition [40]. By situating learning in a playful yet structured environment, *MicroBioCombat* fosters curiosity, reflection and intrinsic motivation, which are critical for developing scientific literacy and lifelong learning habits. Furthermore, the approach prepares students for higher education in biological and health sciences by fostering essential scientific inquiry skills. This aligns with broader evidence that active learning methodologies are not only effective for conceptual mastery but are also critical for increasing student participation and narrowing achievement gaps in Science, Technology, Engineering, and Mathematics curricula [11, 41].

Overall, these findings suggest that integrating educational games like *MicroBioCombat* into microbiology curricula offers a viable, scalable and effective strategy to overcome traditional limitations in teaching complex biological concepts. By combining content mastery with active, collaborative and reflective learning, such tools enhance both understanding and engagement, representing a promising direction for contemporary science education.

## Conclusions

*MicroBioCombat* is an educational card game inspired by *Magic: The Gathering™*, designed to teach bacterial pathologies and to engage students in scenarios involving the interplay between pathogens, transmission vectors and prophylactic strategies. The richness of information embedded in the cards allows students to explore complex microbiological concepts and promotes conceptual understanding in a dynamic and interactive manner. Beyond content acquisition, the playful nature of the game reduces classroom tension, strengthens the relationship between teachers and students and fosters a positive learning environment. By encouraging social interaction, communication and decision-making, *MicroBioCombat* supports the development of both cognitive and socio-emotional skills. Overall, this study demonstrates that *MicroBioCombat* is a promising pedagogical tool for microbiology education, suitable not only for high school but also for the later years of fundamental education. Its combination of ludicity, interdisciplinary content and active engagement positions it as an effective strategy to enhance both learning outcomes and classroom dynamics.

## Supplementary material

10.1099/acmi.0.001238.v3 Supplementary Material 1.
