# Supplementary material for: Game-based learning in microbiology education: design and classroom use of MicroBioCombat
Source: Access Microbiol. 2026 Aug 7;8(8):001238.v3. doi: 10.1099/acmi.0.001238.v3 (PMC13450638; doi:10.1099/acmi.0.001238.v3)
Supplement: Supplementary file 1 [file acmi-8-01238-s001.pdf]

Game-Based Learning in Microbiology Education: Design and Classroom Use of *MicroBioCombat*

By

Raul Vinicius Salata Souto[1], Edmara Rocha dos Santos Moreira[1] and Leandro Marcio Moreira[1,2#]

SUPPLEMENTARY MATERIAL

Supplementary Table 1. Overview of *MicroBioCombat* pathogens and vectors

Nº	Card / Pathogen	Card Type	Cell Struct / Lifestyle	Pathology / Diseases	Game Effect / Function	Energy	Damage points
1	*Bacillus anthracis*	Pathogen	G+ A F	Anthrax	Infects via inhalation or spores; potent attack card	2	4
2	*Bordetella pertussis*	Pathogen	G- A	Whooping cough	Spread via droplets; damages opponent when used with "Spittle"	3	3
3	*Brucella sp.*	Pathogen	G- A F	Brucellosis	Spread via contaminated meat or vectors	2	2
4	*Clostridium botulinum*	Pathogen	G+ An P	Botulism	Entry via contaminated food; potent toxin card	2	5
5	*Clostridium perfrigens*	Pathogen	G+ An P	Gas gangrene	Entry via wounds; damages opponent if combined with "Deep Wounds"	2	2
6	*Clostridium tetani*	Pathogen	G+ An P	Tetanus	Requires anaerobic wounds; damages over rounds	3	4
7	*Corynebacterium diphtheriae*	Pathogen	G+ A F	Diphtheria	Attack enhanced by "Lisogenic phage"; associated with throat infection	1	2
8	*Escherichia coli*	Pathogen	G- A F	Gastroenteritis	Can become pathogenic via "Biological risk" card	2	3
9	*Helicobacter pylori*	Pathogen	G- M	Peptic disease	Colonizes stomach; damage increased by "Stress"	1	3
10	*Leptospira interrogans*	Pathogen	G- A	Leptospirosis	Spread via rats and vectors	2	4
11	*Listeria monocytogenes*	Pathogen	G+ A F	Listeriosis	Enhanced by "Pregnancy" card; food-borne	3	4
12	*Mycobacterium tuberculosis*	Pathogen	*G+ A	Tuberculosis	Targets lungs; defended by neutrophils/macrophages	3	2
13	*Mycobacterium leprae*	Pathogen	*G+ A	Hansen's disease	Entry via open wounds; chronic infection card	3	3
14	*Neisseria gonorrhoeae*	Pathogen	G- A	Gonorrhea	Sexual transmission; attack card	1	2
15	*Neisseria meningitidis*	Pathogen	G- A	Meningitis	Spread via droplets; enhanced by "Spittle"	4	4
16	*Rickettsia prowazekii*	Pathogen	G- A	Epidemic typhus	Vector-borne; transmitted by lice	1	2
17	*Rickettsia rickettsii*	Pathogen	G- A	Rocky Mountain spotted fever	Vector-borne; transmitted by ticks	3	4
18	*Rickettsia typhi*	Pathogen	G- A	Endemic typhus	Transmitted by *Pediculus humanus*	1	2
19	*Salmonella sp.*	Pathogen	G- A F	Salmonellosis	Food-borne; entry enhanced by "Contaminated meat" or "Unpasteurized milk"	2	3
20	*Staphylococcus aureus*	Pathogen	G+ A F	Generalized infections	Entry via wounds; attack card	3	3
21	*Staphylococcus saprophyticus*	Pathogen	G+ A F	Cystitis	Sexual transmission; damage card	1	2
22	*Streptococcus pneumoniae*	Pathogen	G+ A	Bacterial pneumonia	Respiratory pathogen; combined with droplets	3	3
23	*Streptococcus pyogenes*	Pathogen	G+ A	Erysipelas	Entry via wounds; attack enhanced by throat infection	2	2
24	*Treponema pallidum*	Pathogen	G- M	Syphilis	Sexual transmission; attack card	3	2
25	*Vibrio cholerae*	Pathogen	G- A F	Cholera	Food/water transmission; strong damage card	1	3
26	*Yersinia pestis*	Pathogen	G- A N F	Bubonic plague	Vector-borne; enhanced via rat and flea cards	2	4

Cell Structure (Gram Staining): G+ (Gram-positive) - Indicates bacteria with a thick peptidoglycan cell wall; G- (Gram-negative) - Indicates bacteria with a thinner cell wall and an additional outer membrane. Lifestyle (Oxygen Metabolism): A (Aerobe) - Microorganisms that require oxygen to grow; An (Anaerobe) - Microorganisms that grow in the absence of oxygen; F (Facultative) - Also known as Facultative Anaerobes or microorganisms that can grow in both the presence and absence of oxygen. M (Microaerophilic) - Used to indicate specific atmospheric requirements or neutral growth conditions. P (Spore-former / Pleomorphic) - Refers to the ability to form spores for environmental survival. * Poorly visualized by Gram staining.

Supplementary Table 2. Defense and protection cards in *MicroBioCombat*

N°	Card Name	Card Type	Target	Effect / Function	Energy
1	Anaerobic environment	Environmental	Aerobic bacteria in attack	Kills all aerobic bacteria; prevails over defense cards	2
2	Aerobic environment	Environmental	Anaerobic bacteria in attack	Kills all anaerobic bacteria; prevails over defense cards	2
3	Penicillin	Antibiotic	*Treponema pallidum*, *Streptococcus pyogenes*	Destroys bacterial colonies of target pathogens	2
4	Phenol (carbolic acid)	Disinfectant	*Mycobacterium leprae*, *Mycobacterium tuberculosis*	Used with “1%” card to neutralize attacking bacteria	2
5	Biphenols (hexachlorophene)	Disinfectant	Gram-positive (*Streptococcus*, *Staphylococcus*)	Kills targeted bacteria on attack	1
6	Triclosan	Disinfectant	Gram-positive bacteria	Prevents attack for 2 rounds	2
7	Halogens (iodine)	Disinfectant	Open wound / Deep wound pathogens	Prevents pathogen entry for 1 round	2
8	Healing ointment	Treatment	—	Promotes recovery; assists immune defense	1
9	Platelets	Immune	—	Supports defense against attacking bacteria	1
10	Chlorine	Chemical defense	Water-borne bacteria	Protects individual for 2 rounds when combined with “Water” card	3
11	Alcohol 70%	Disinfectant	All bacteria	Protects individual for 1 round	2
12	Sodium nitrite	Food safety	Bacteria in “Contaminated meat”	Neutralizes attack; bacteria return to deck	2
13	Research and treatment	Medical	Attacking bacteria	Paralyzes attack for 2 rounds	1
14	Quaternary ammonium compounds	Disinfectant	Throat infection bacteria (except *Pseudomonas*)	Neutralizes attacks for 2 rounds; less effective in organic matter	2
15	Slow freezing	Physical defense	Attacking bacteria	Kills bacteria after 3 rounds via crystal formation	1
16	Fast freezing	Physical defense	Attacking bacteria	Paralyzes attack for 2 rounds; does not kill	1
17	Autoclave	Sterilization	All attacking bacteria	Kills all bacteria; requires “15 min” card	2
18	Pasteurization	Sterilization	All attacking bacteria	Kills bacteria from attacking opponent	5
19	Vitamin C	Immune booster	Macrophages / Neutrophils	Increases destructive power against 2 bacteria	2
20	Neutrophil	Immune	1 bacterium in attack	Kills bacteria; enhanced with Vitamin C / T4 lymphocytes	2
21	Macrophage	Immune	1 bacterium in attack	Kills bacteria; enhanced with Vitamin C / T4 lymphocytes	4
22	Aniquilating Serpent	Vector control	Rats transmitting plague	Removes rat cards from play; returns to deck	2
23	Poison against lice and ticks	Vector control	Lice / ticks	Eliminates vector cards; returns them to deck	3
24	Care in sexual relations	Behavioral	Sexually transmitted pathogens	Protects for 2 rounds	1
25	Decomposer bactéria	Biological	Food-borne bacteria	Removes multiplying bacteria from table	2
26	Health surveillance	Public health	Food-borne bacteria	Eliminates pathogens causing foodborne illness	0
27	Physical exercises	Immune booster	1 bacterium	Increases immune cell attack power	2
28	Keep calm	Behavioral	Stress card	Neutralizes “Stress” effect	1
29	*Bedellovibrio*	Bacteriophage / biological	1 Gram-negative bacterium	Kills targeted bacterium	1
30	Medical prescription	Antibiotic support	—	Enhances action of antibiotics cards	2
31	DPT vaccine	Vaccine	Diphtheria, Pertussis, Tetanus bacteria	Provides immunity to targeted pathogens	0
32	Water	Environmental	Chlorine card	Activates chlorine effectiveness against bacteria	1
33	Surfactant	Disinfectant	Surface pathogens / biofilms	Reduces pathogen adhesion; blocks attack for 1 round	4

Supplementary Table 3. Additional mechanisms and protection conditions

N°	Mechanism / Condition	Card Type	Target	Game Effect / Function	Energy
1	Bacterial transformation	Microbial process	Attacking bacteria	Increases genetic variability; evades antibiotic action	2
2	Bad eating habits	Host factor	Host immune cells	Reduces macrophage and neutrophil efficiency	1
3	Biological risk	Fostering	Commensal bacteria	Converts commensals into pathogens	2
4	Blood flow	Host factor / Fostering	Parasites / vectors	Allows direct entry of blood-borne pathogens	0
5	Conjugation	Microbial process	Attacking bacteria	Enhances variability; avoids antibiotics (except autoclave/pasteurization/surfactant)	2
6	Contaminated meat	Food-borne	Food-borne pathogens	Carries multiple pathogens; enables transmission	1
7	Deep wounds	Vector / Entry	Severe bacterial pathogens	Enables high-damage infections	2
8	Fibrin	Pathogen enhancer	All bacteria	Increases pathogen damage	2
9	Inhalation of spores	Vector	*Bacillus anthracis*	Enables infection via respiratory route	0
10	Kidney dysfunction	Host factor	*Streptococcus*	Induces nephritis if combined	0
11	Lysogenic phage	Fostering	*Corynebacterium*	Potentiates bacterial attack	2
12	Open wounds	Vector / Entry	Various bacteria	Enables pathogen entry	2
13	*Pediculus humanus*	Vector	*Rickettsia typhi*	Facilitates endemic typhus transmission	0
14	Pregnancy	Host factor / Fostering	*Listeria monocytogenes*, *Neisseria meningitidis*	Increases damage when associated	1
15	Rheumatic fever / Valve stenosis	Host factor	*Streptococcus*	Causes damage points depending on combination	0
16	Spittle	Vector / Fostering	Respiratory pathogens	Enhances pathogen spread	1
17	Spore intake	Vector	*Bacillus anthracis*, *Salmonella* spp.	Reintroduces bacteria; simulates rapid proliferation	1
18	Star tick bite	Vector	*Rickettsia rickettsii*	Facilitates Rocky Mountain spotted fever transmission	0
19	Stress	Fostering	Pathogens	Increases damage caused by pathogens	1
20	Throat infection	Host factor	*Streptococcus*	Boosts attack if combined with relevant cards	1
21	Unpasteurized milk	Food-borne	Food-borne bacteria	Reintroduces bacteria up to 3 times; simulates rapid proliferation	2
22	*Xenopsylla cheopis* + Sewer rat	Vector	*Yersinia pestis*	Facilitates bubonic plague transmission	0

Game-Based Learning in Microbiology Education: Design and Classroom Use of *MicroBioCombat*

Raul Vinicius Salata Souto[1], Edmara Rocha dos Santos Moreira[1] and Leandro Marcio Moreira[1,2#]

Supplementary File 1:

Game cards for printing.

Bacillus anthracis

2

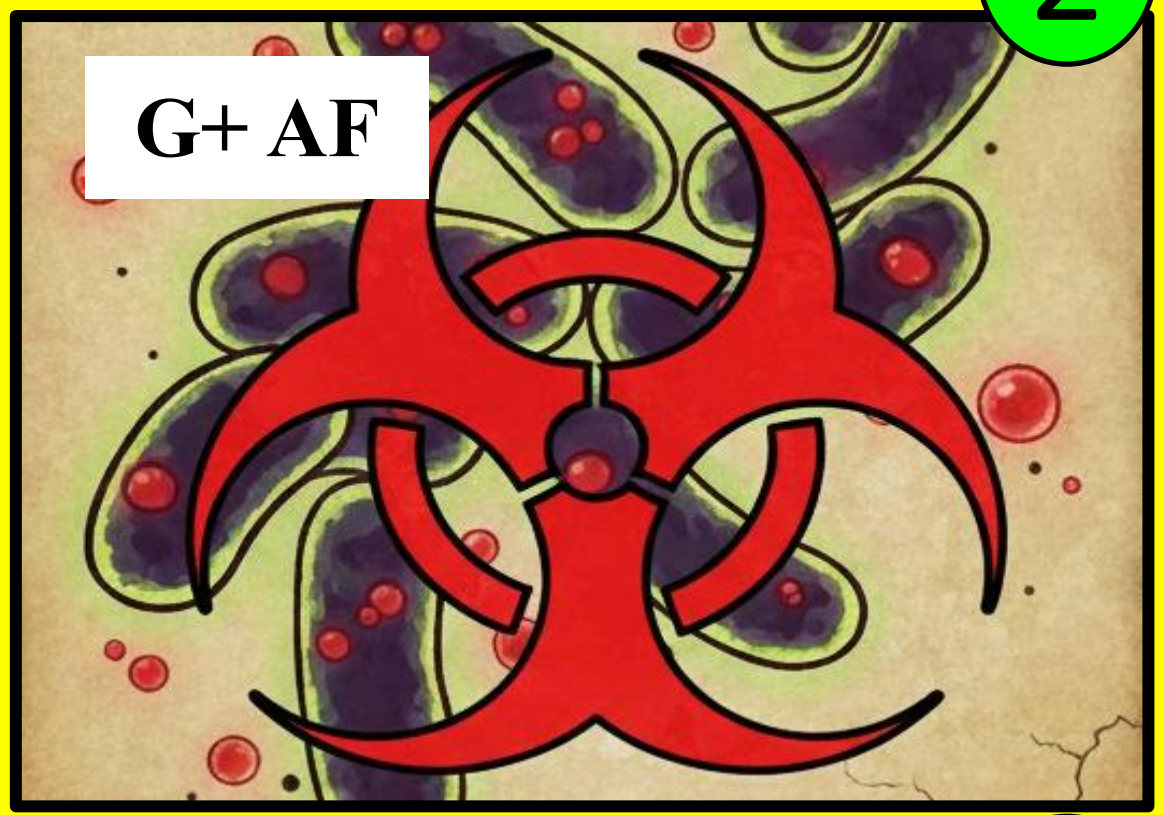

4

Infects via inhalation or spores; potent attack card

Bordetella pertussis

3

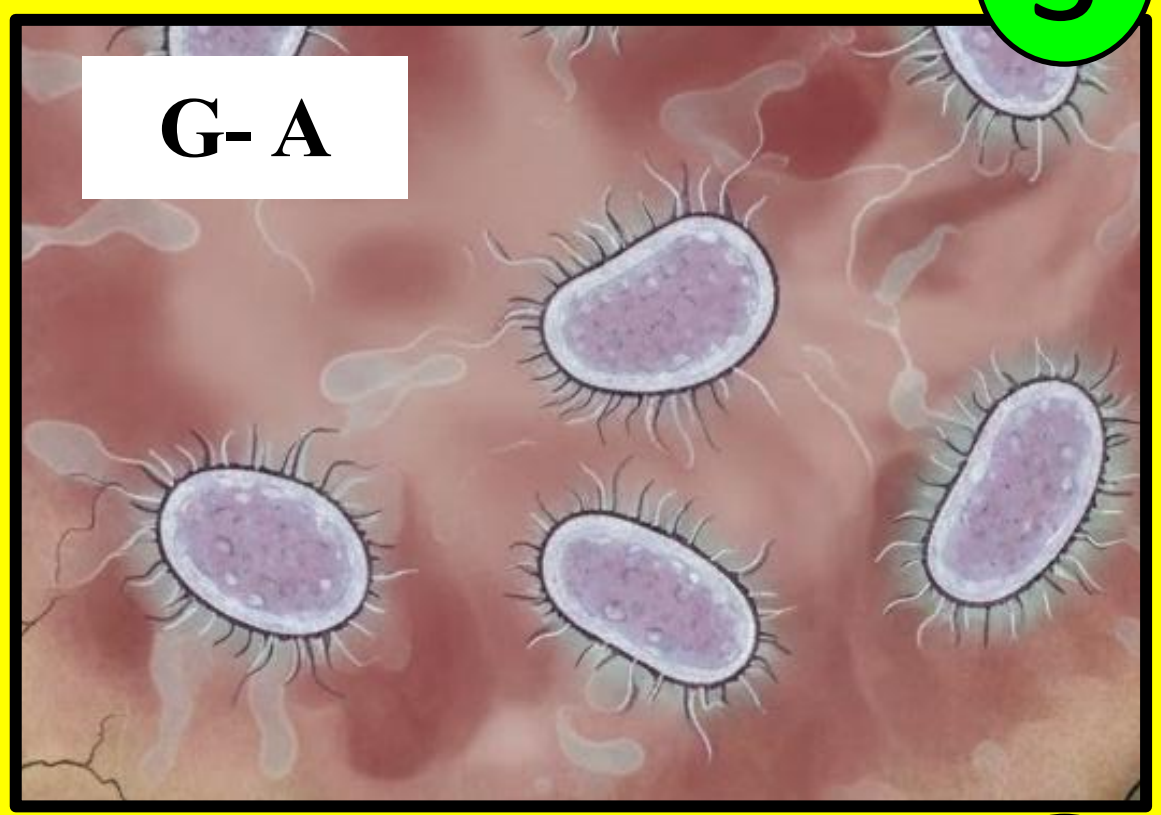

3

Spread via droplets; damages opponent when used with “Spittle”

Brucella sp.

2

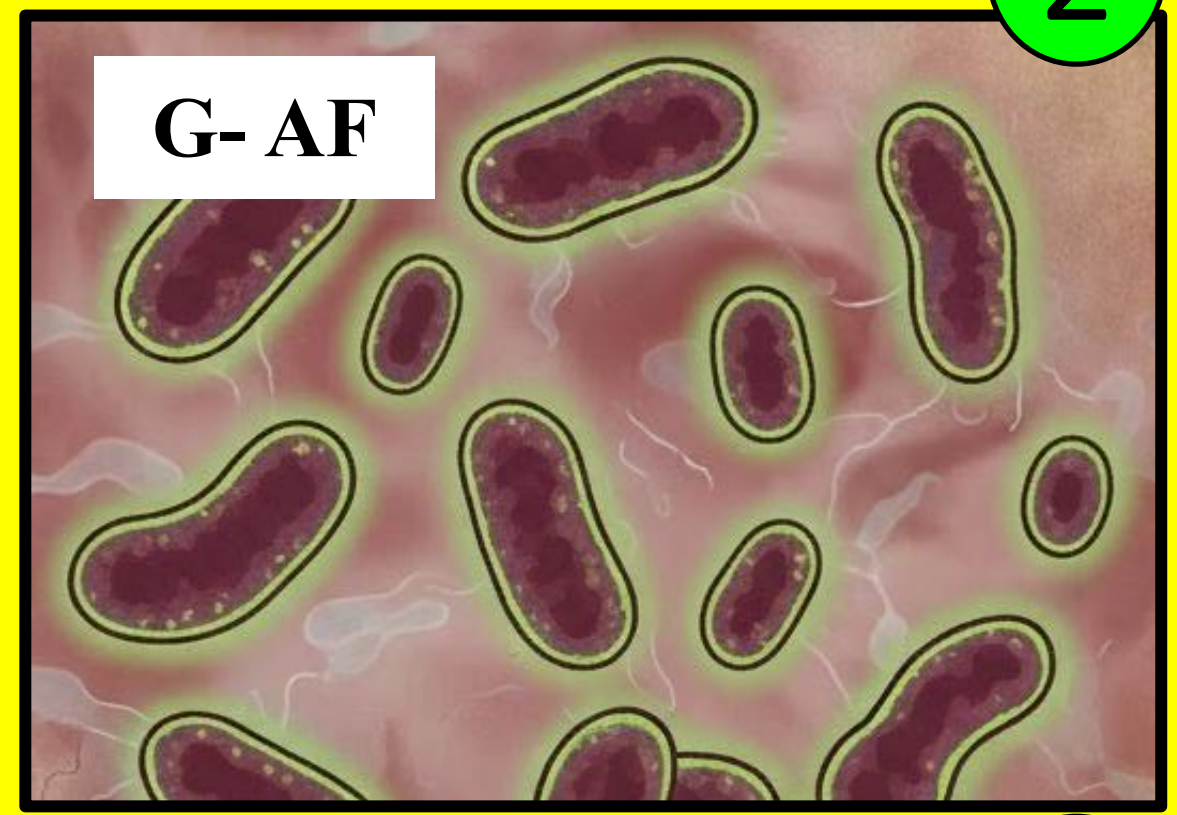

2

Spread via contaminated meat or vectors

Clostridium botulinum

2

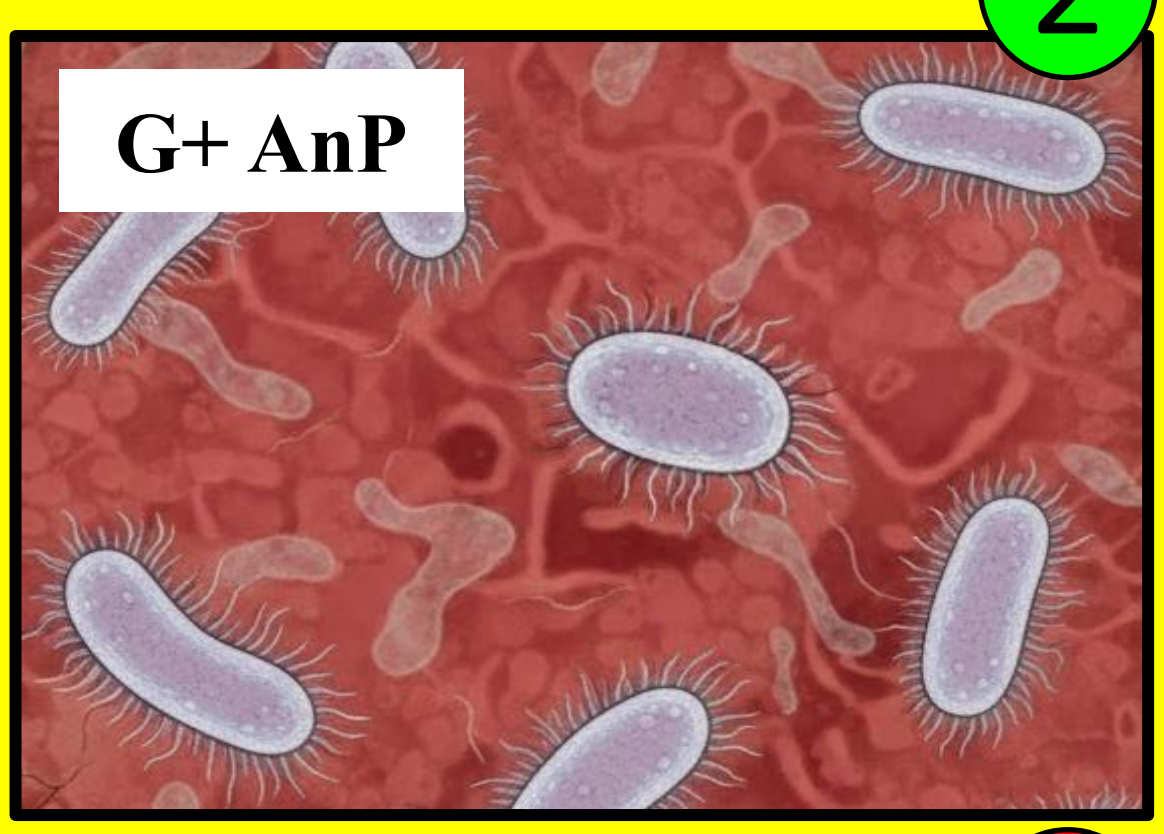

5

Entry via contaminated food; potent toxin card

Clostridium perfrigens

2

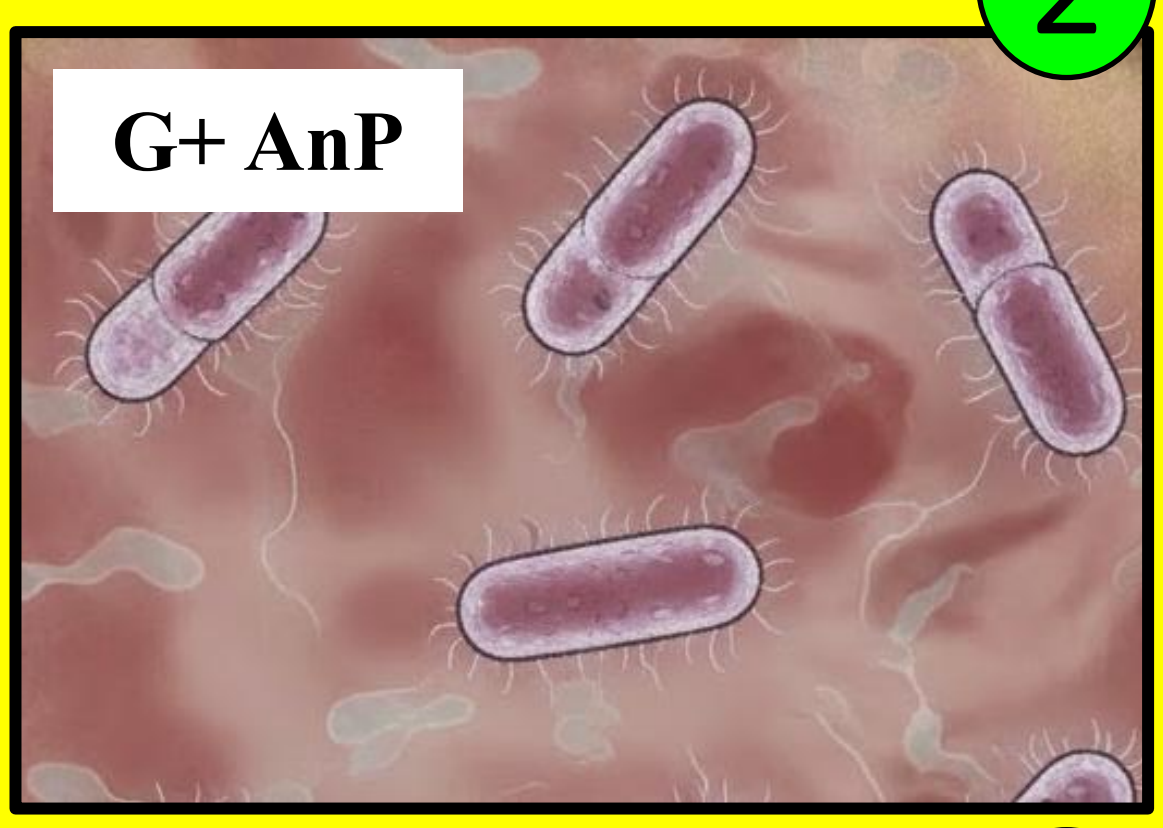

2

Entry via wounds; damages opponent if combined with “Deep Wounds”

Clostridium tetani

3

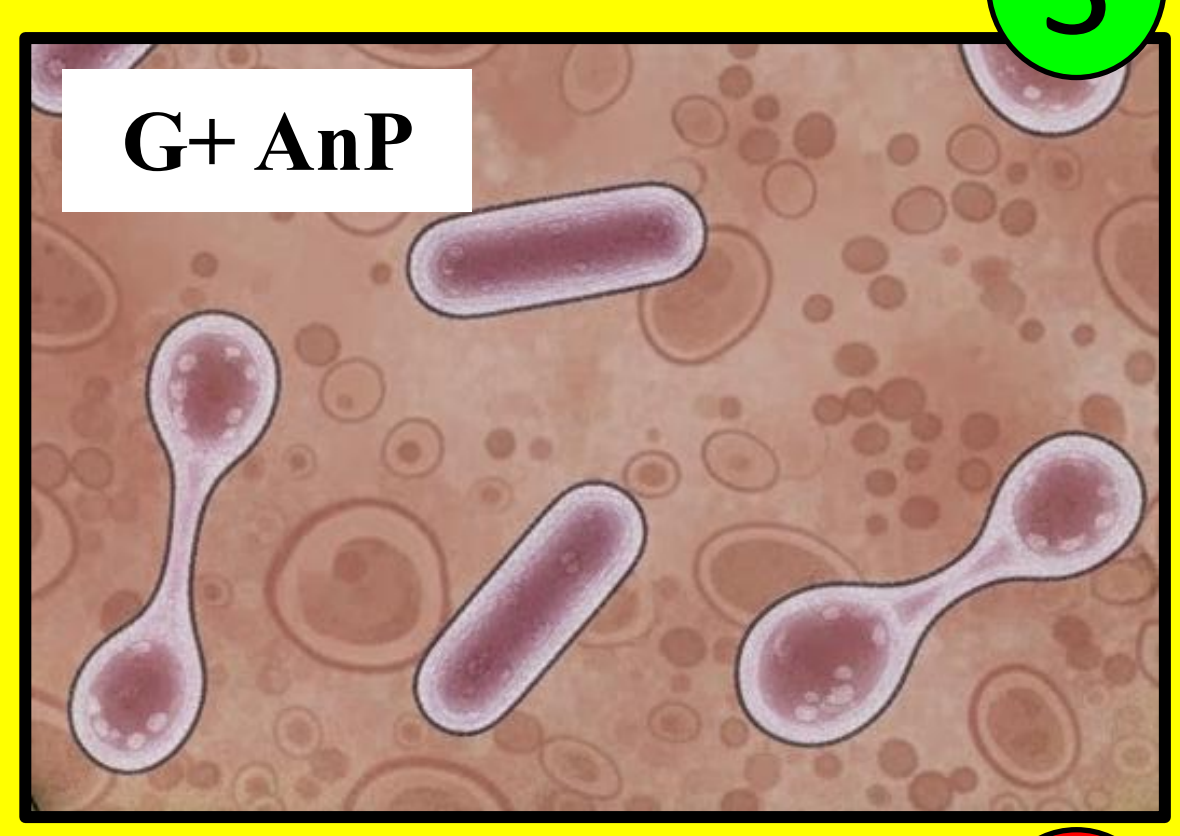

4

Requires anaerobic wounds; damages over rounds

Corynebacterium diphtheriae

1

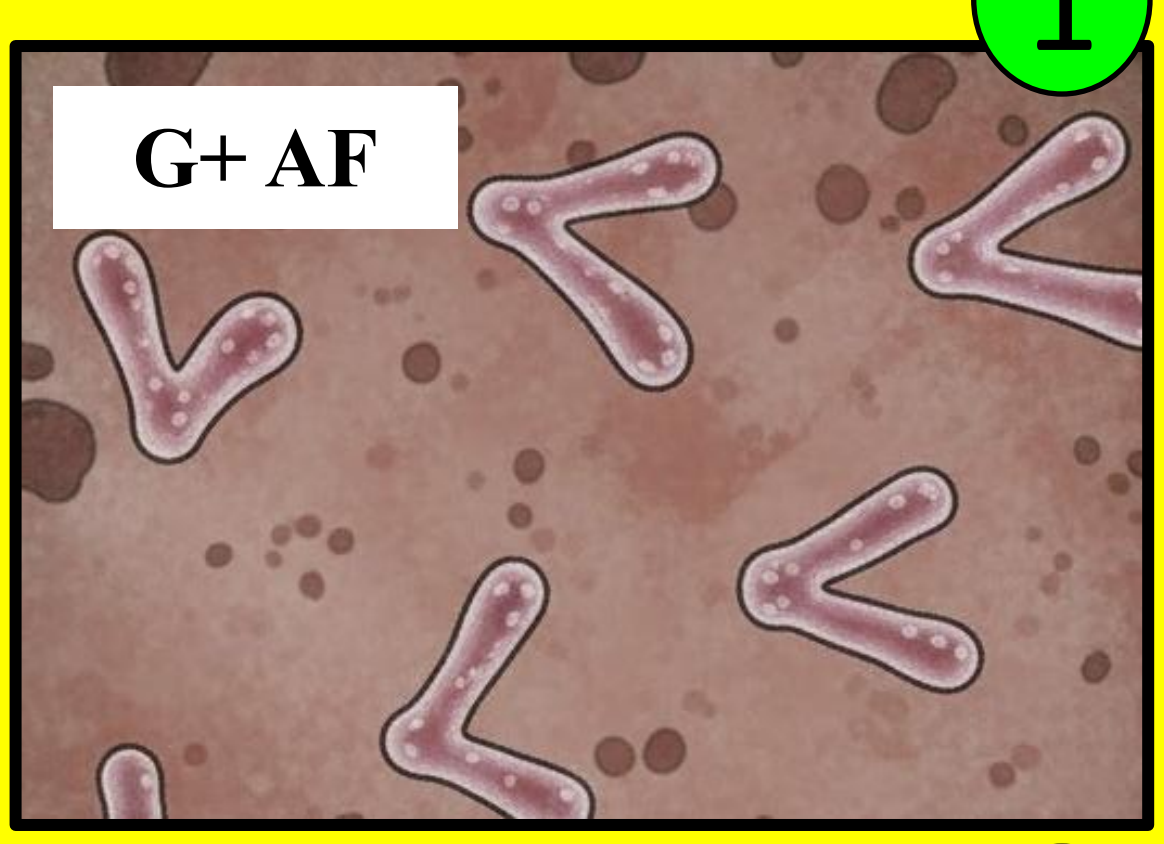

2

Attack enhanced by “Lisogenic phage”; associated with throat infection

Escherichia coli

2

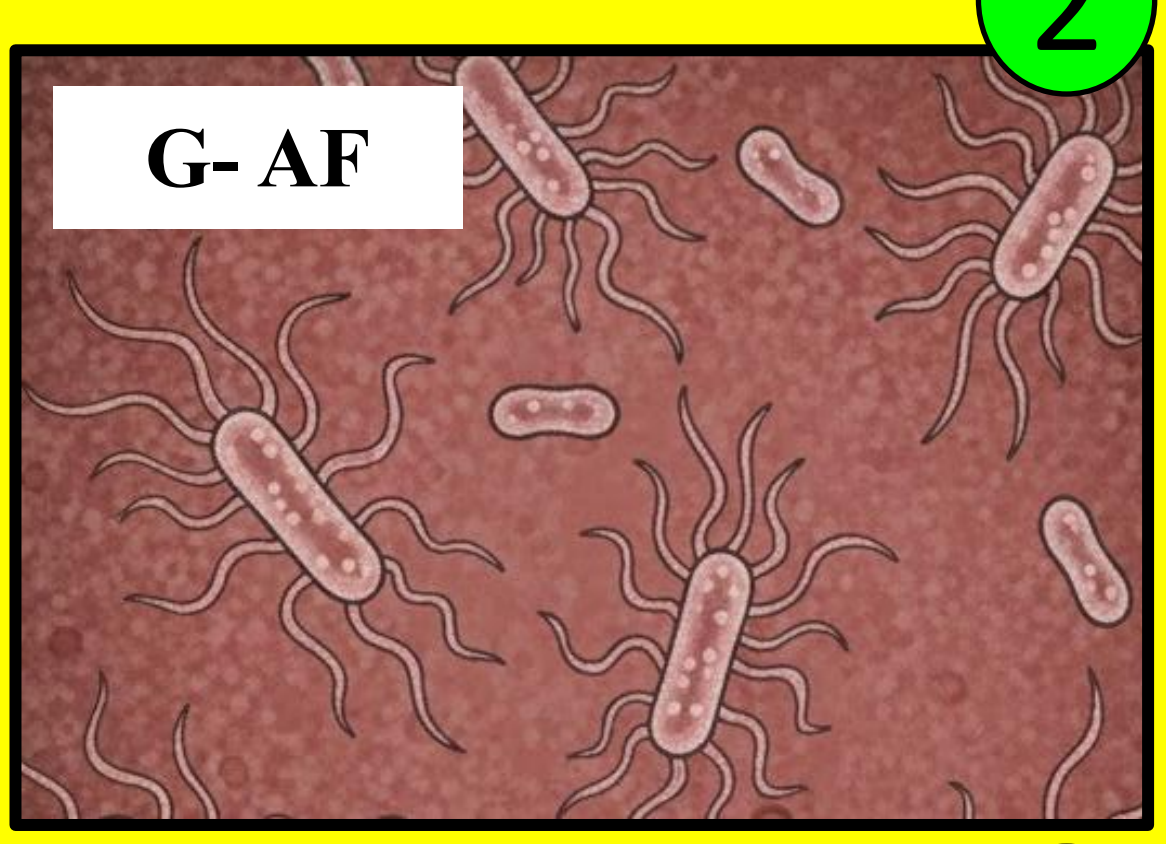

3

Can become pathogenic via “Biological risk” card

Helicobacter pylori

1

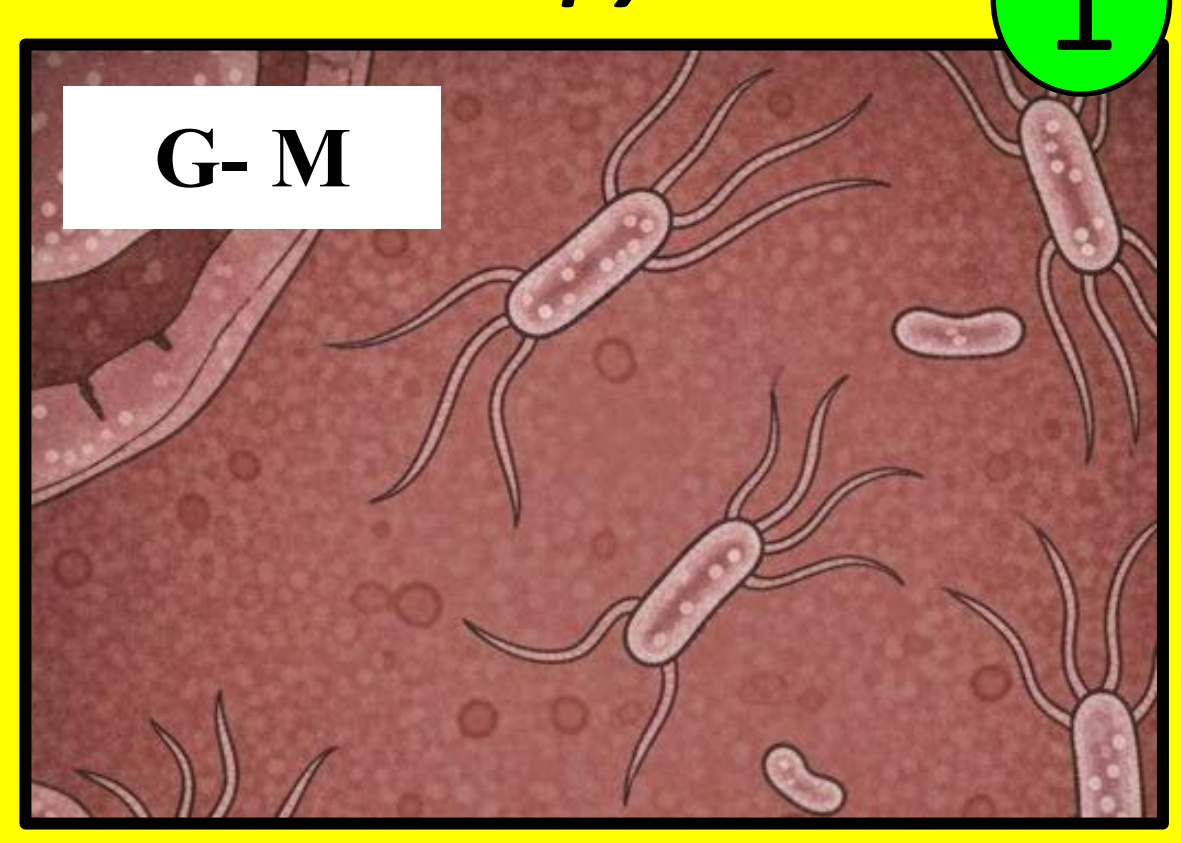

3

Colonizes stomach; damage increased by “Stress”

Leptospira interrogans 2

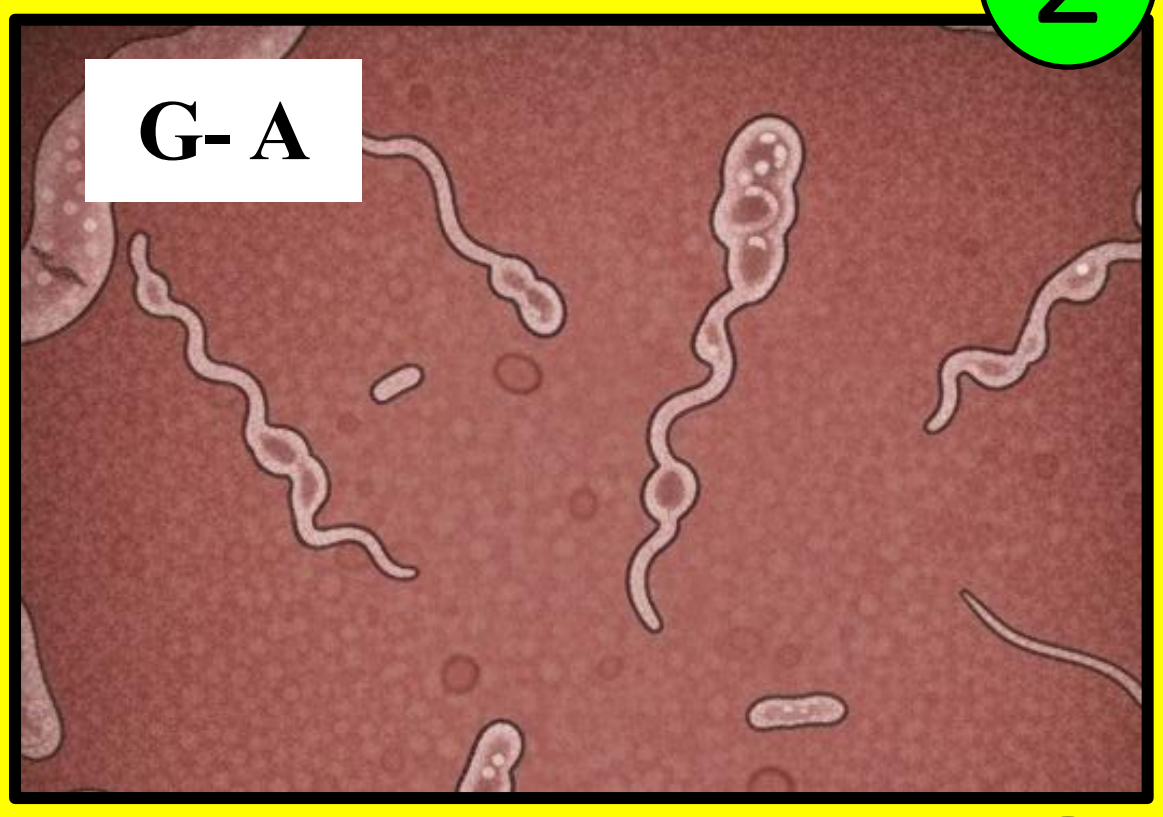

4

Spread via rats and vectors

Listeria monocytogenes 3

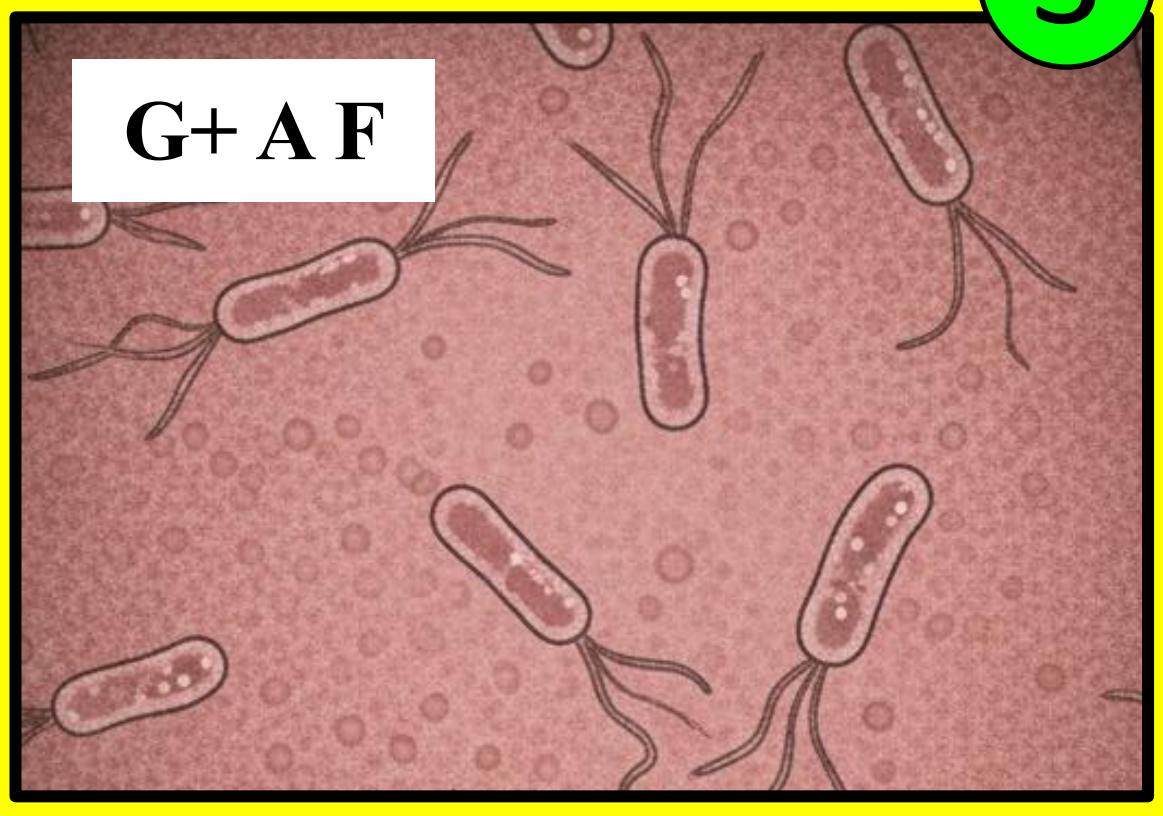

4

Enhanced by "Pregnancy" card; food-borne

Mycobacterium tuberculosis 3

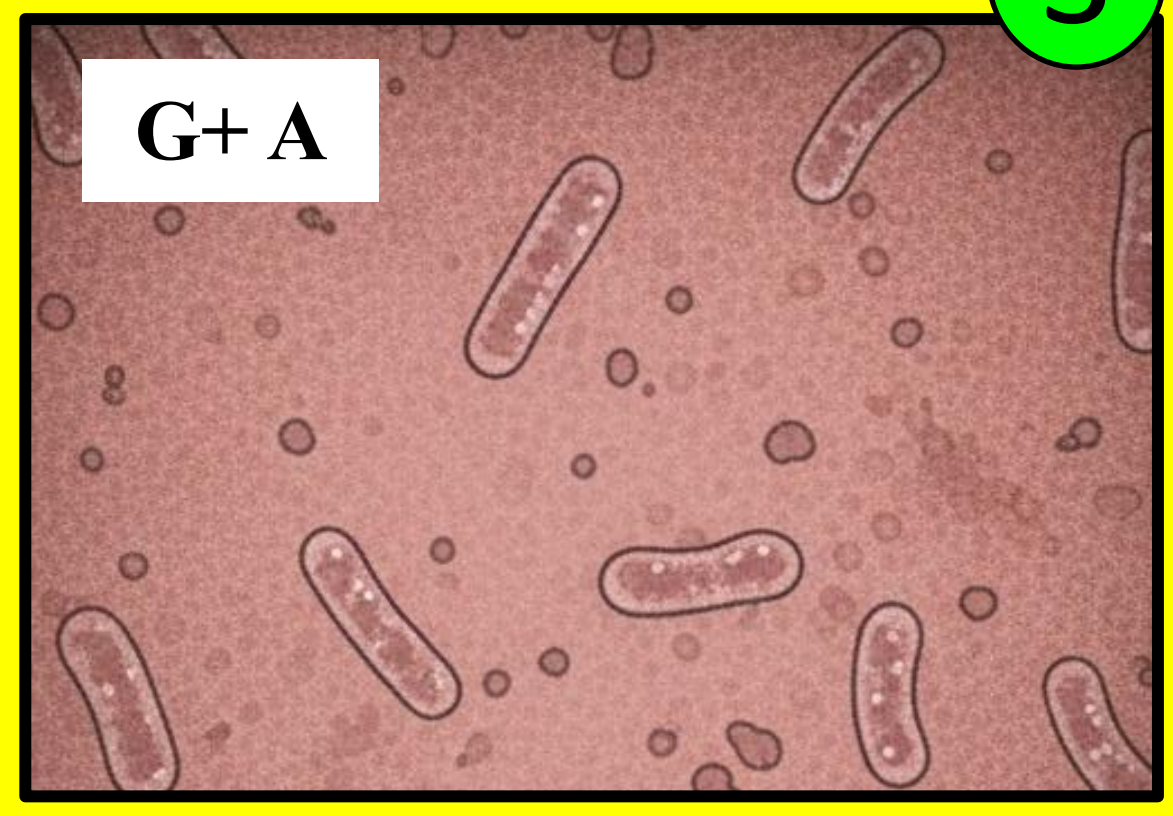

2

Targets lungs; defended by neutrophils or macrophages

Mycobacterium leprae 3

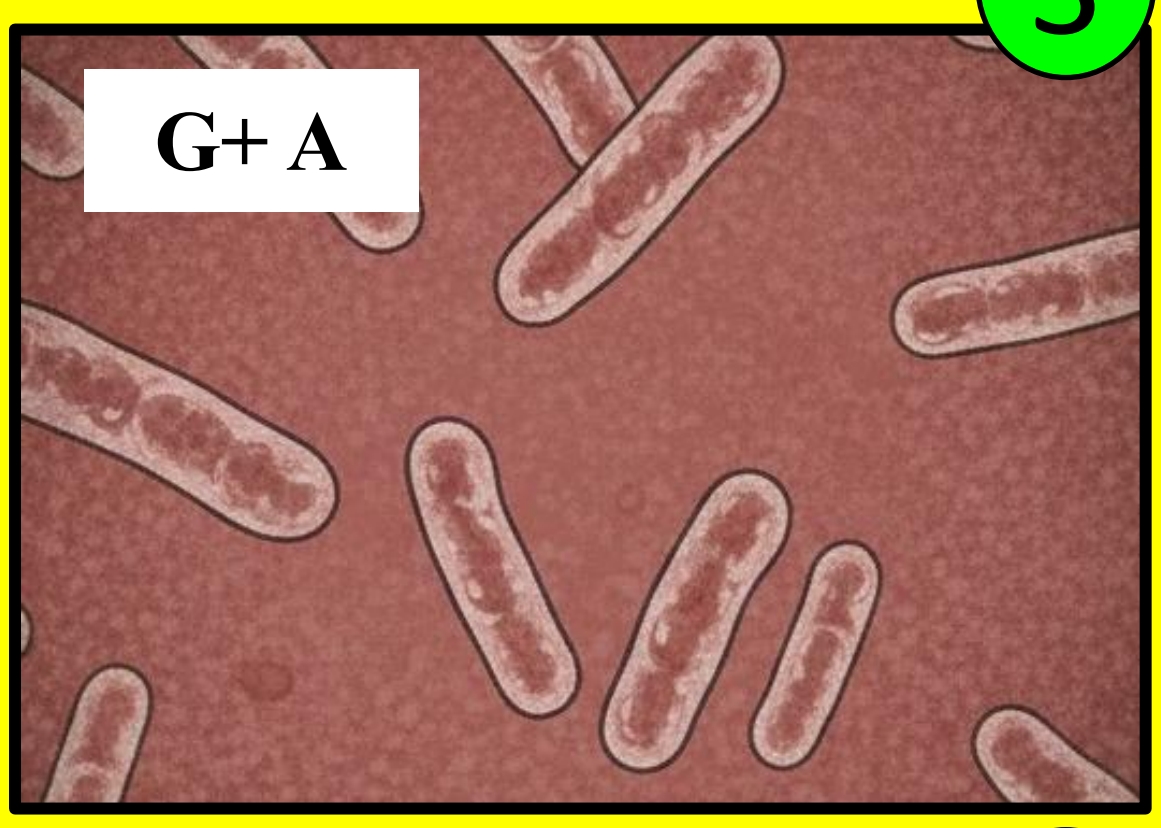

3

Entry via open wounds; chronic infection card.

Neisseria gonorrhoeae 1

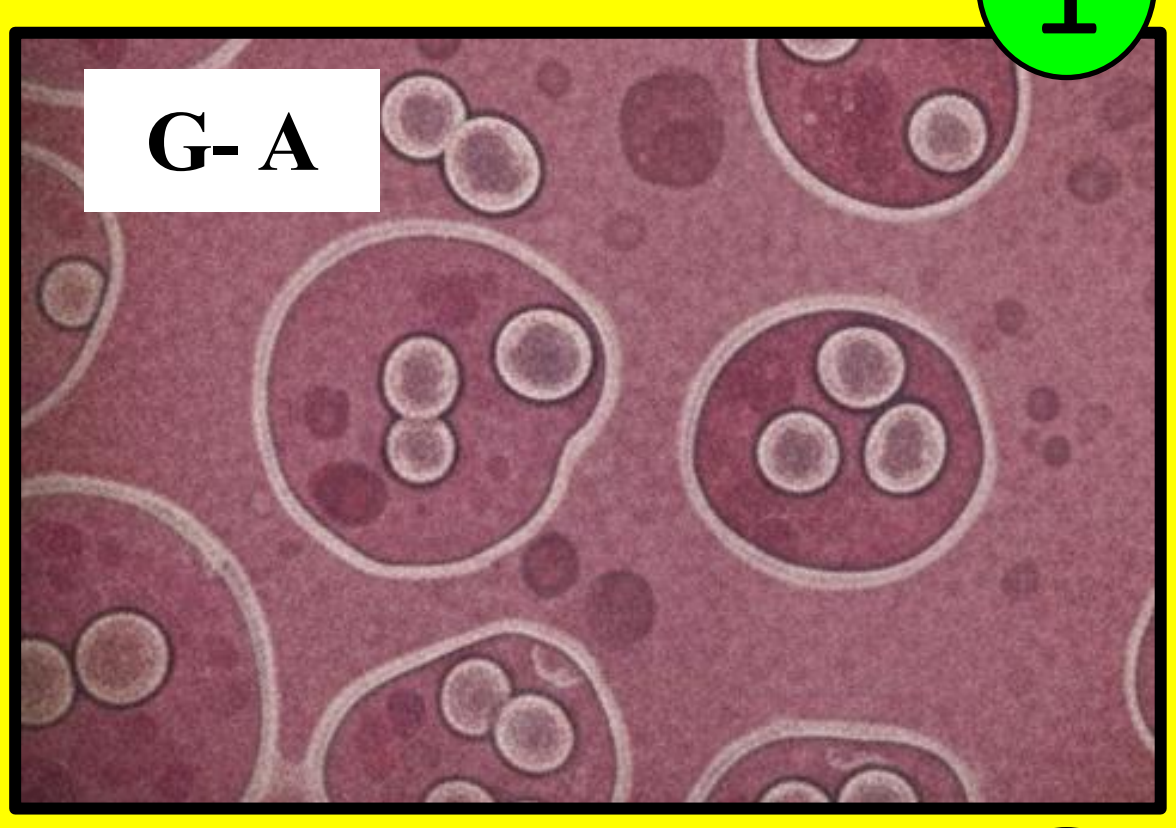

2

Sexual transmission; attack card.

Neisseria meningitidis 4

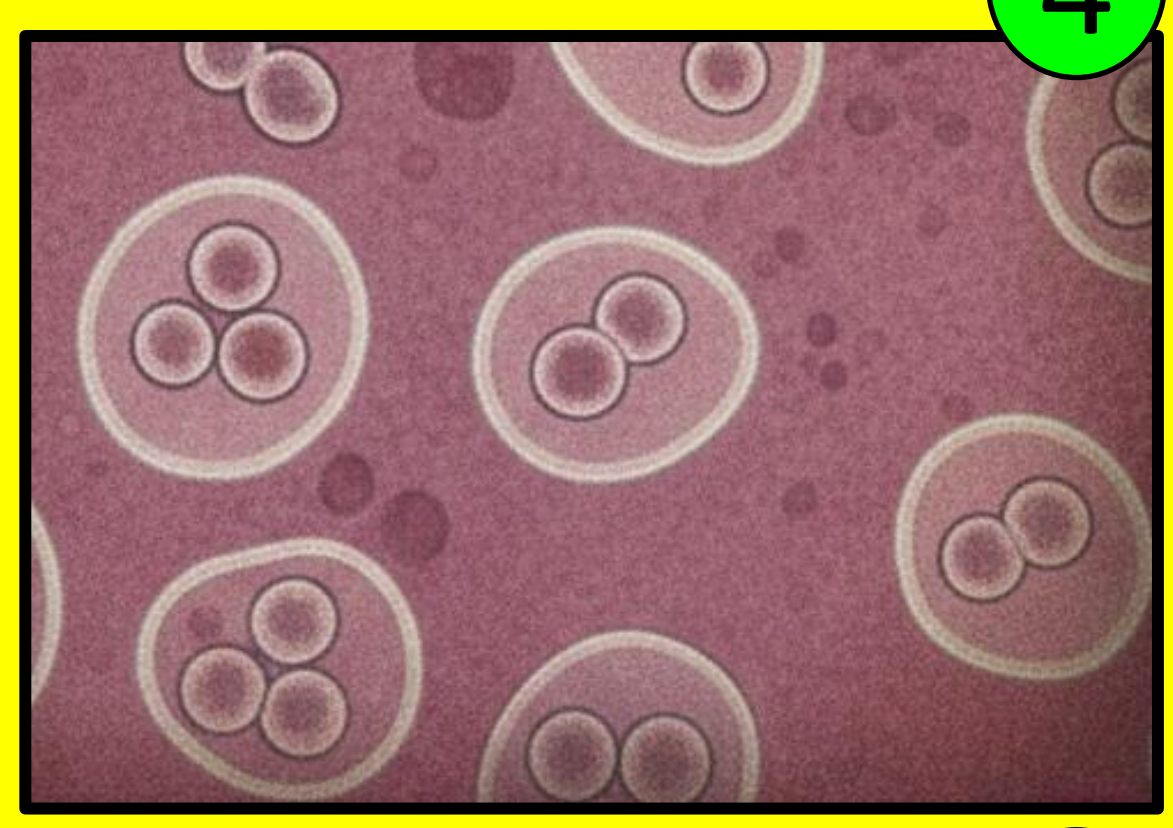

4

Spread via droplets; enhanced by "Spittle"

Rickettsia prowazekii 1

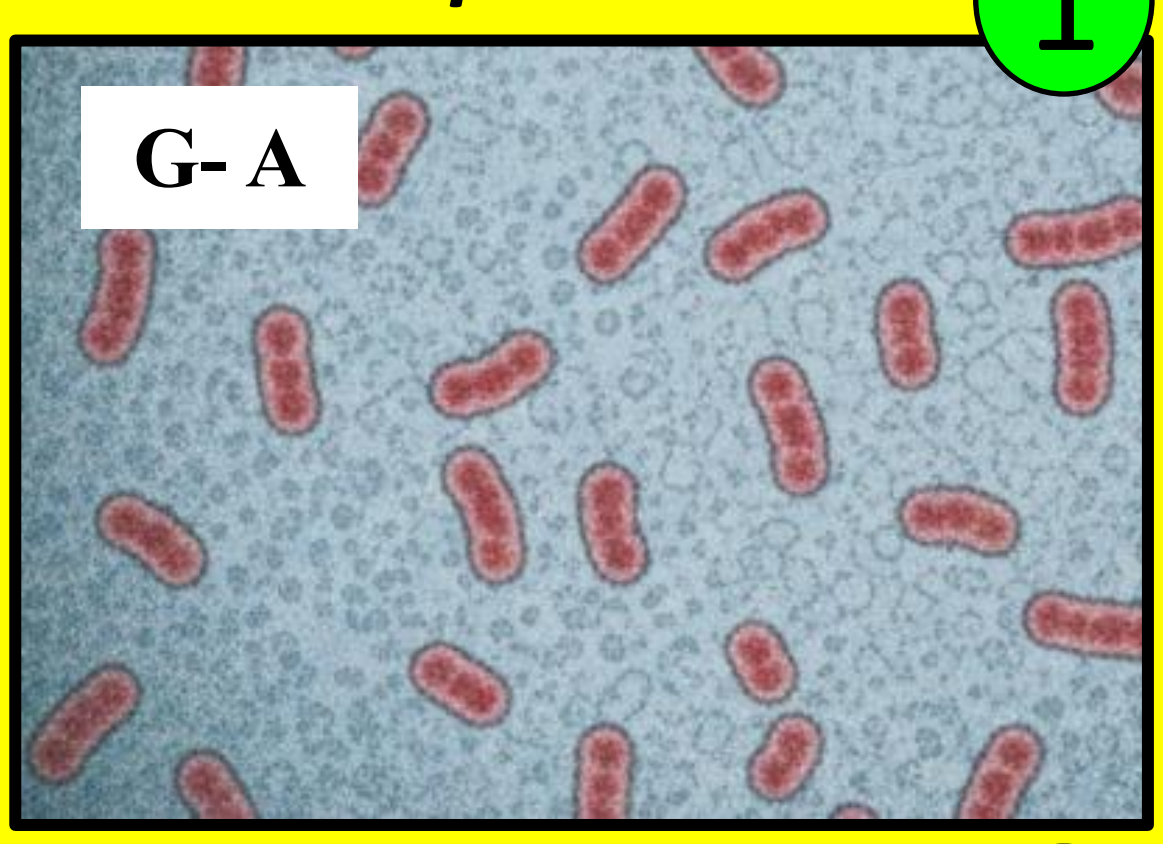

2

Vector-borne; transmitted by lice.

Rickettsia rickettsii 3

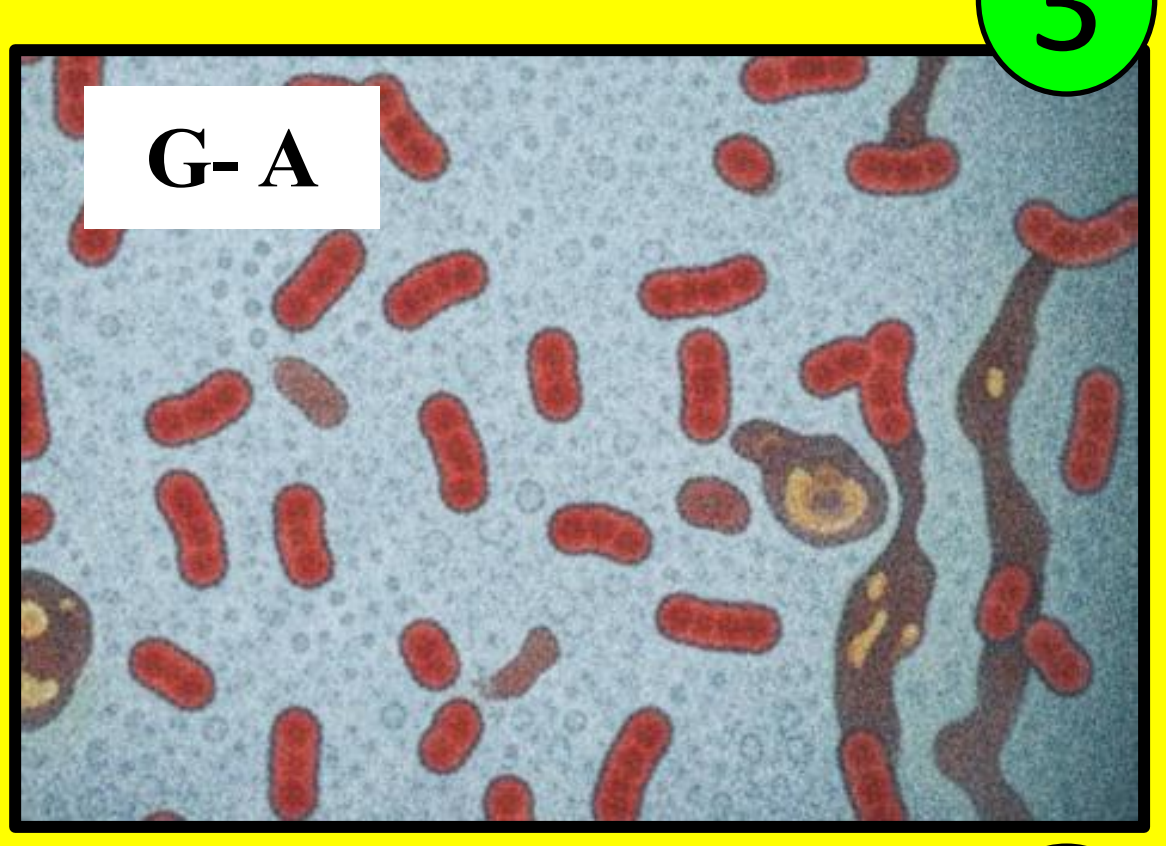

4

Vector-borne; transmitted by ticks.

Rickettsia typhi 2

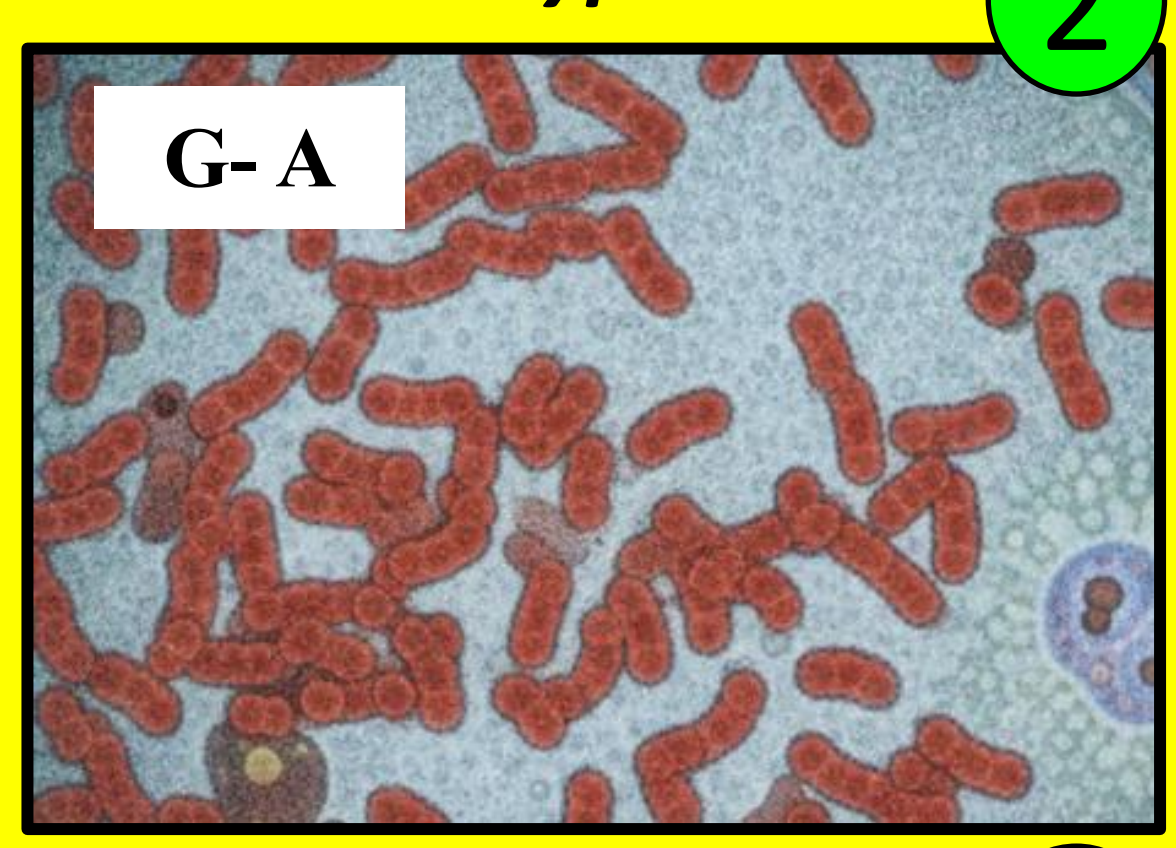

4

Transmitted by *Pediculus humanus*.

Salmonella 2

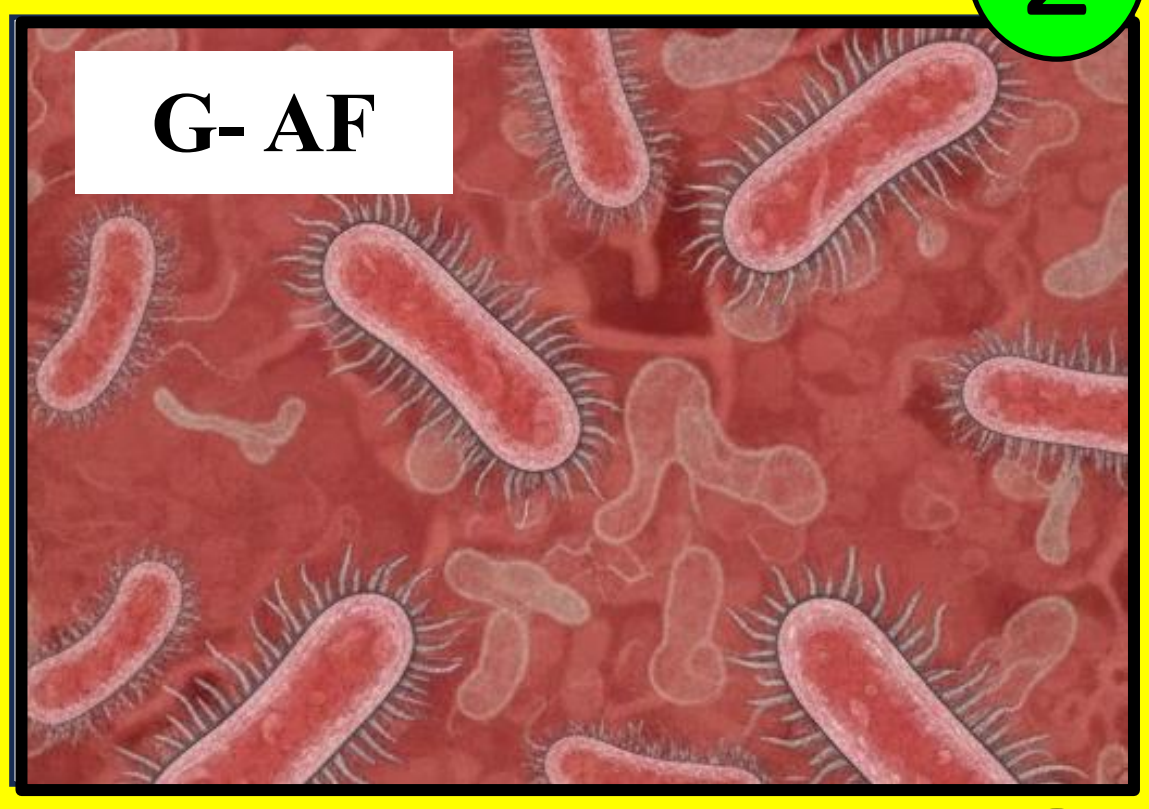

3

Food-borne; entry enhanced by "Contaminated meat" or "Unpasteurized milk".

Staphylococcus aureus 3

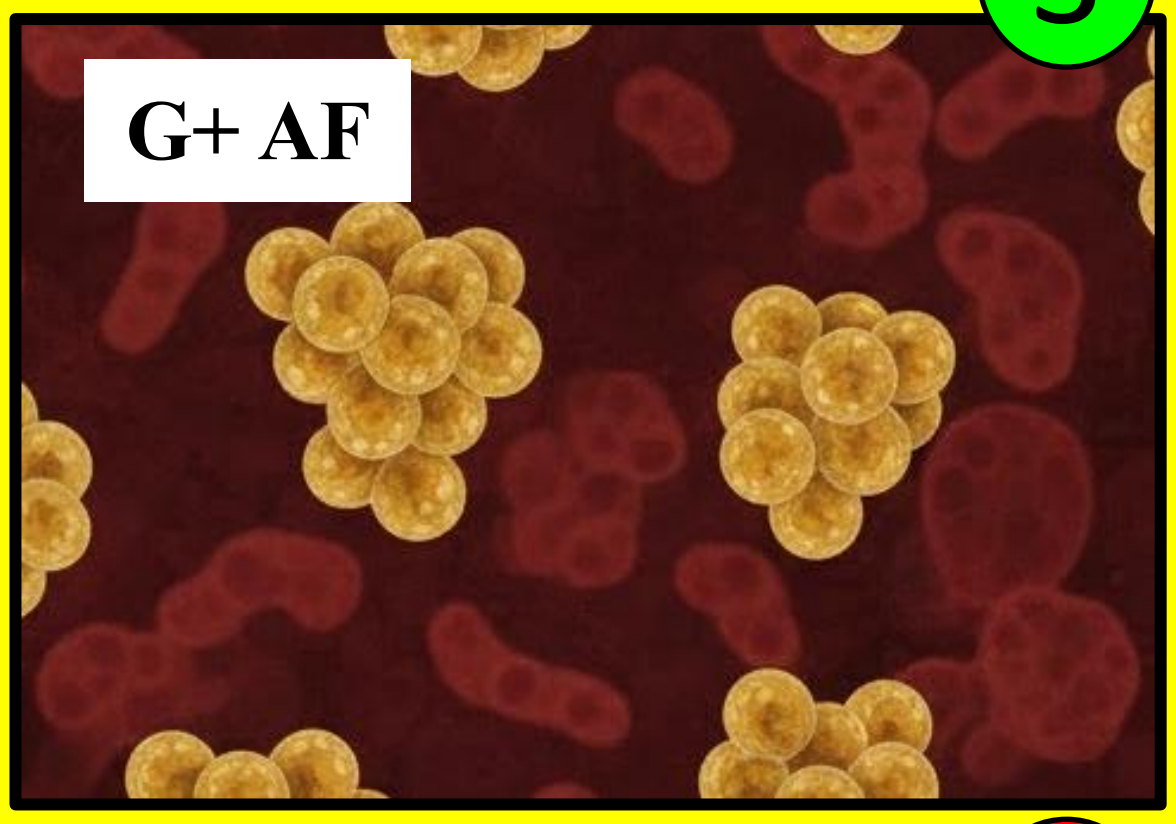

3

Entry via wounds; attack card.

Staphylococcus saprophyticus 1

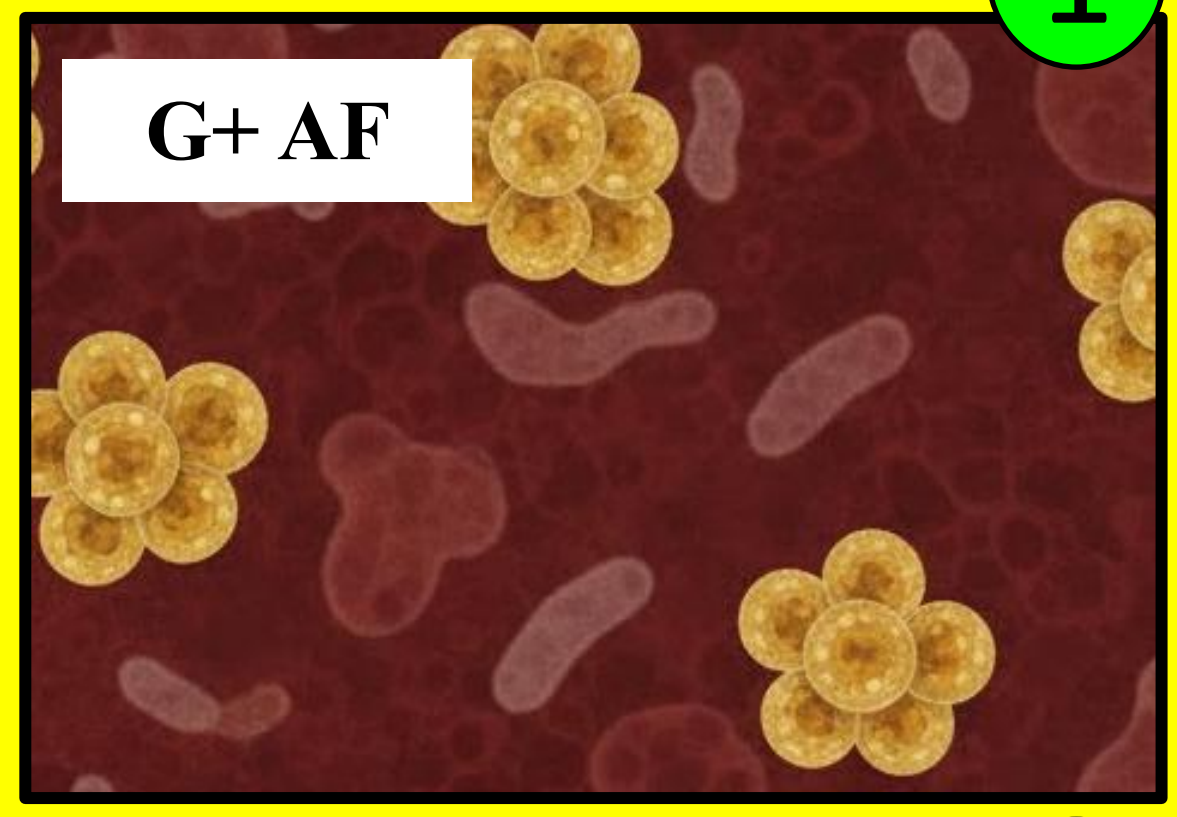

2

Sexual transmission; damage card.

Streptococcus pneumoniae 3

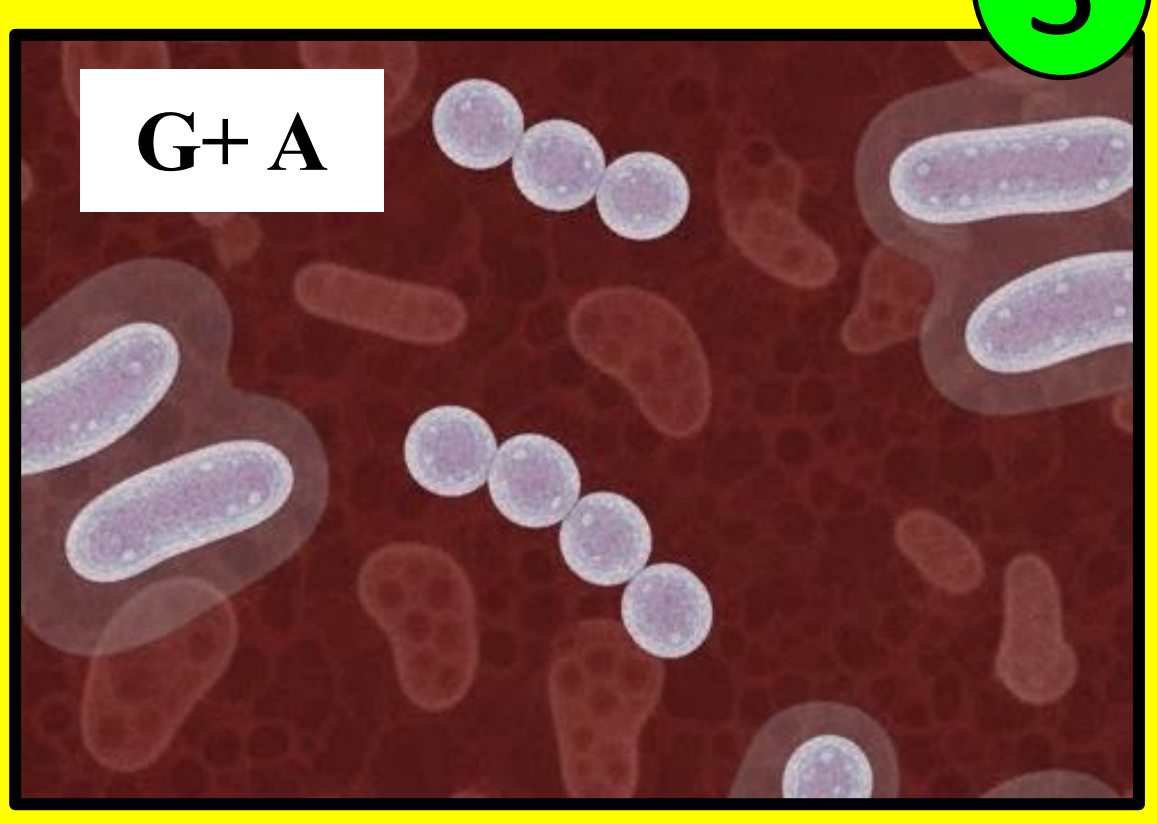

3

Respiratory pathogen; combined with droplets.

Streptococcus pyogenes 2

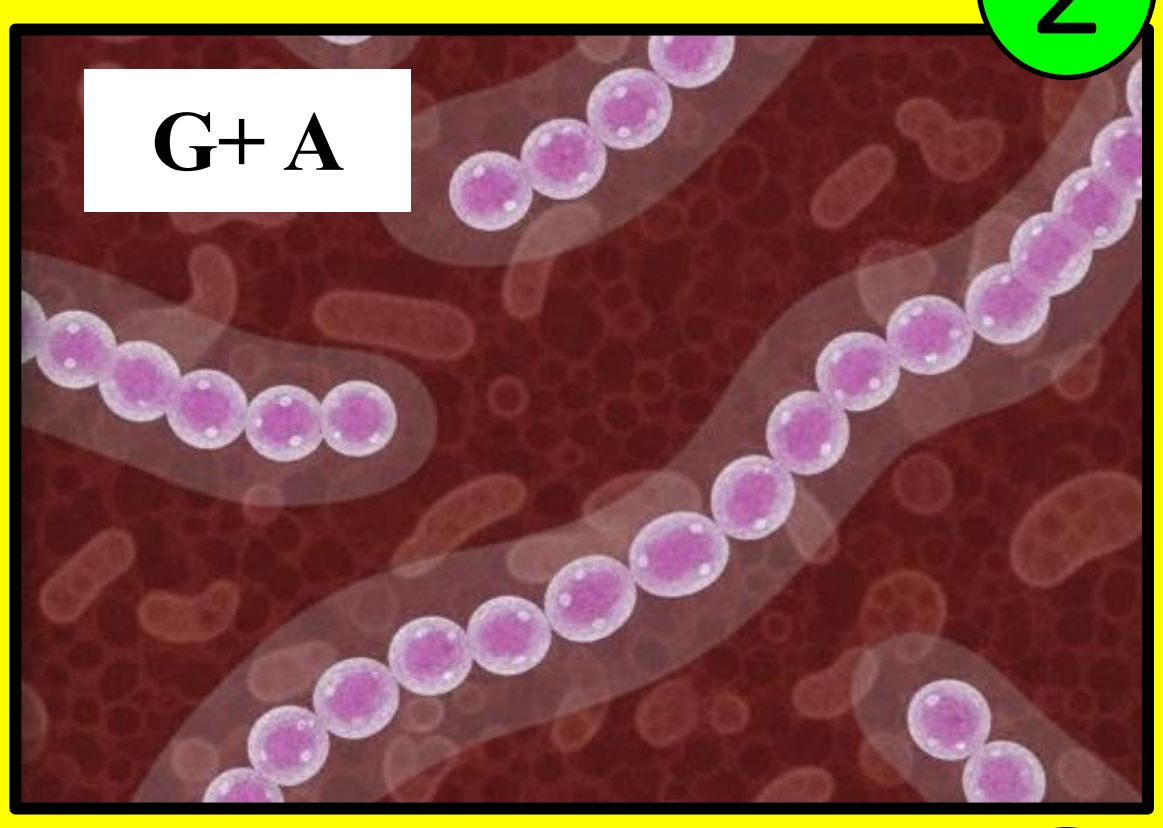

2

Entry via wounds; attack enhanced by throat infection.

Treponema pallidum 3

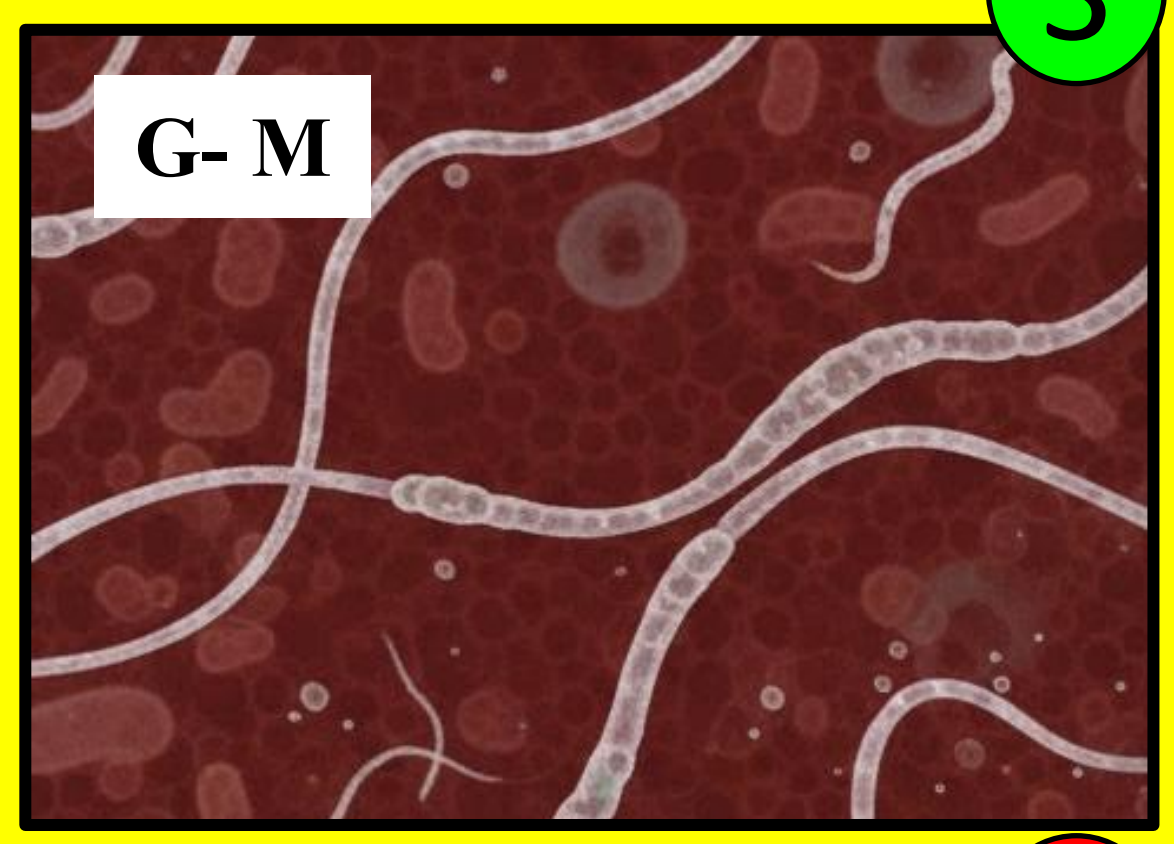

2

Sexual transmission; attack card.

Vibrio cholerae 1

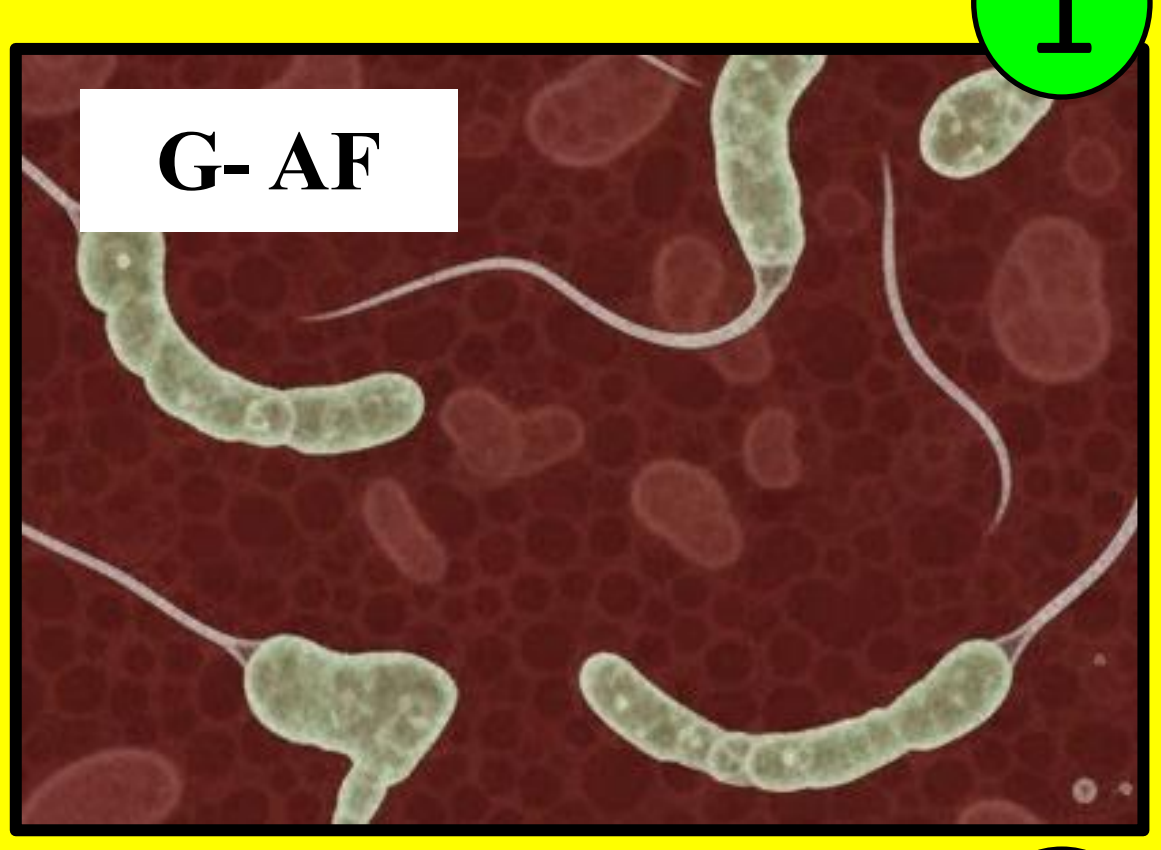

3

Food/water transmission; strong damage card.

Yersinia pestis 2

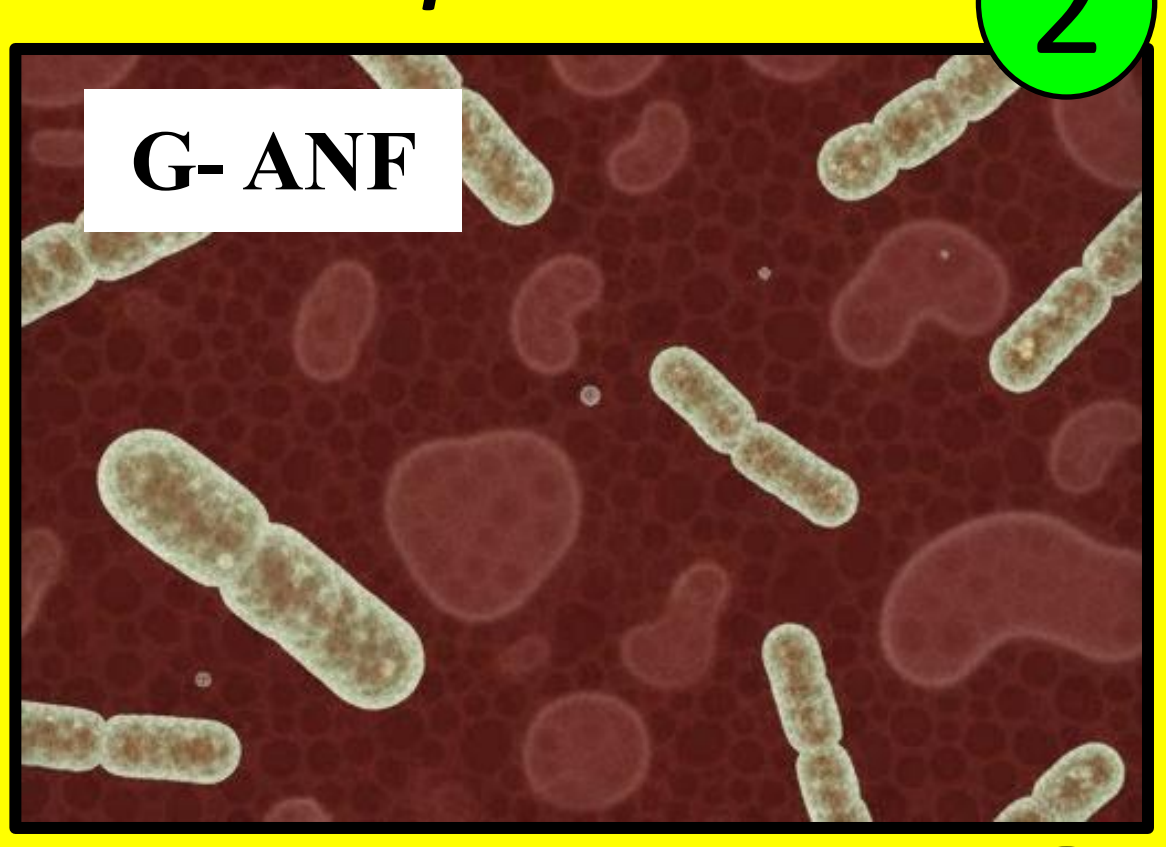

4

Vector-borne; enhanced via rat and flea cards

Anaerobic environment 2

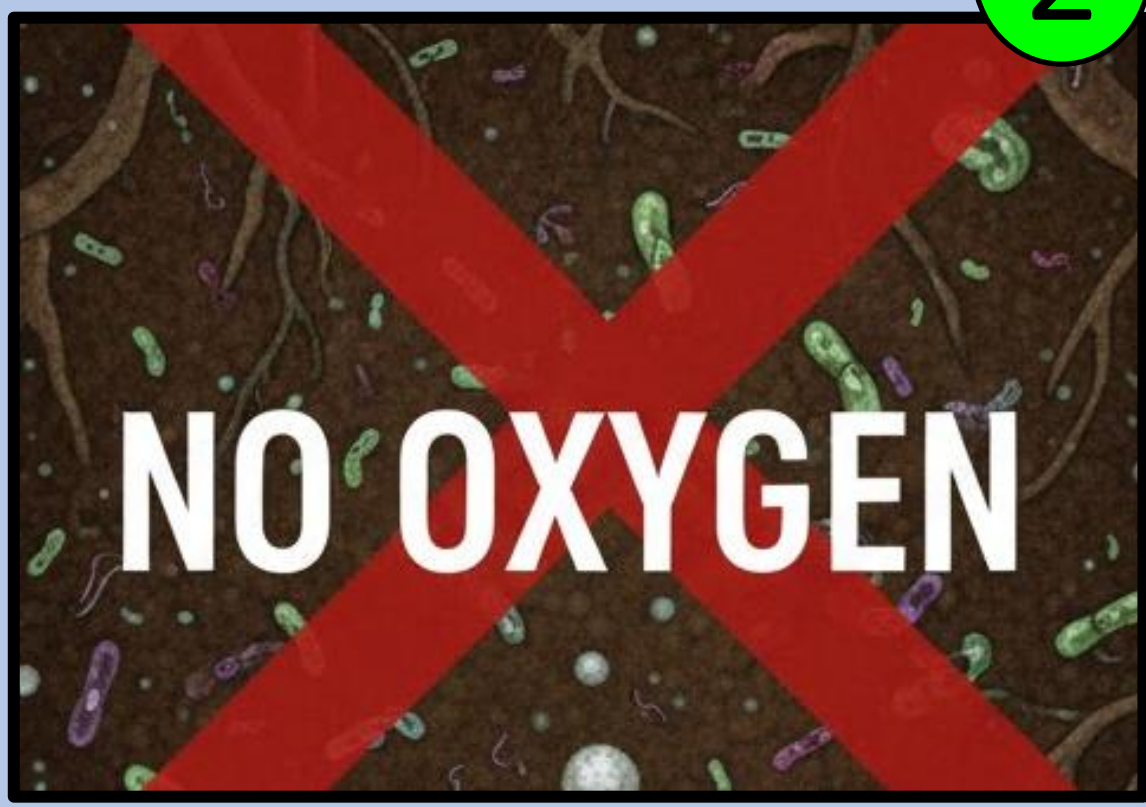

Aerobic bacteria in attack. Kills all aerobic bacteria; prevails over defense cards.

Aerobic environment 2

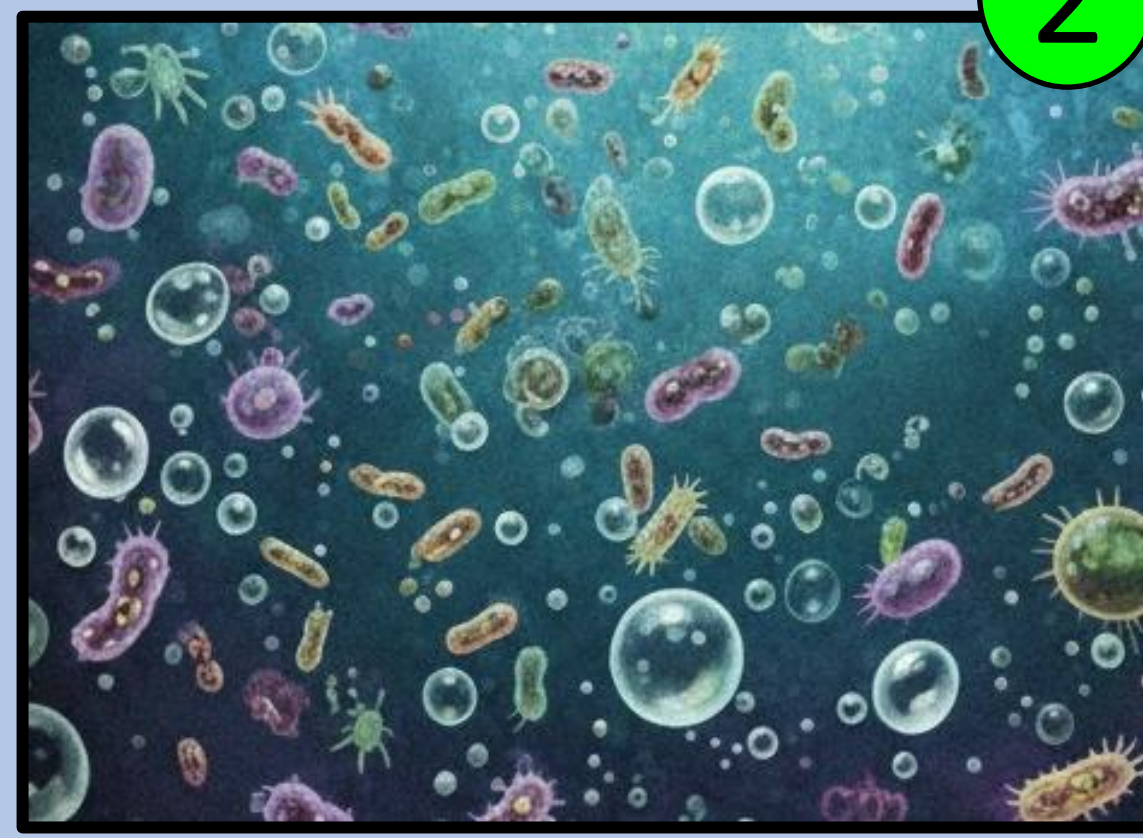

Anaerobic bacteria in attack. Kills all anaerobic bacteria; prevails over defense cards

Penicillin 2

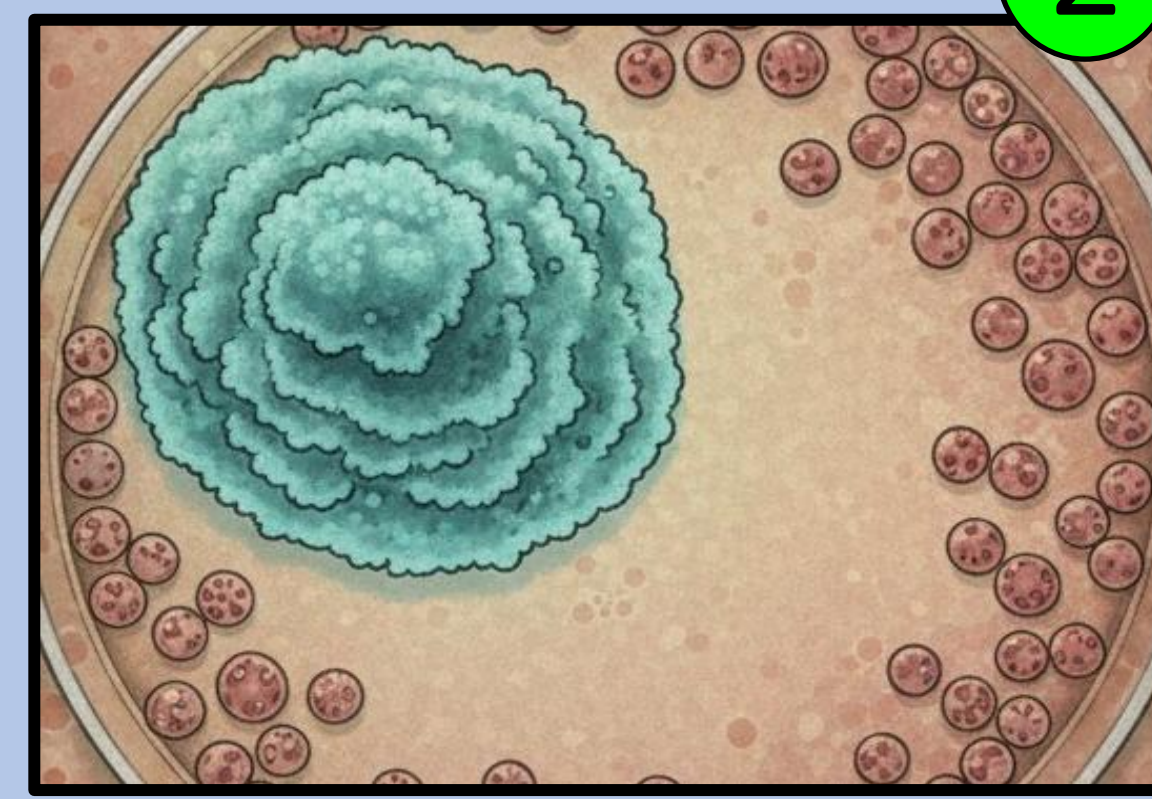

Treponema pallidum and *Streptococcus pyogenes*. Destroys bacterial colonies of target pathogens

Phenol (carbolic acid) 2

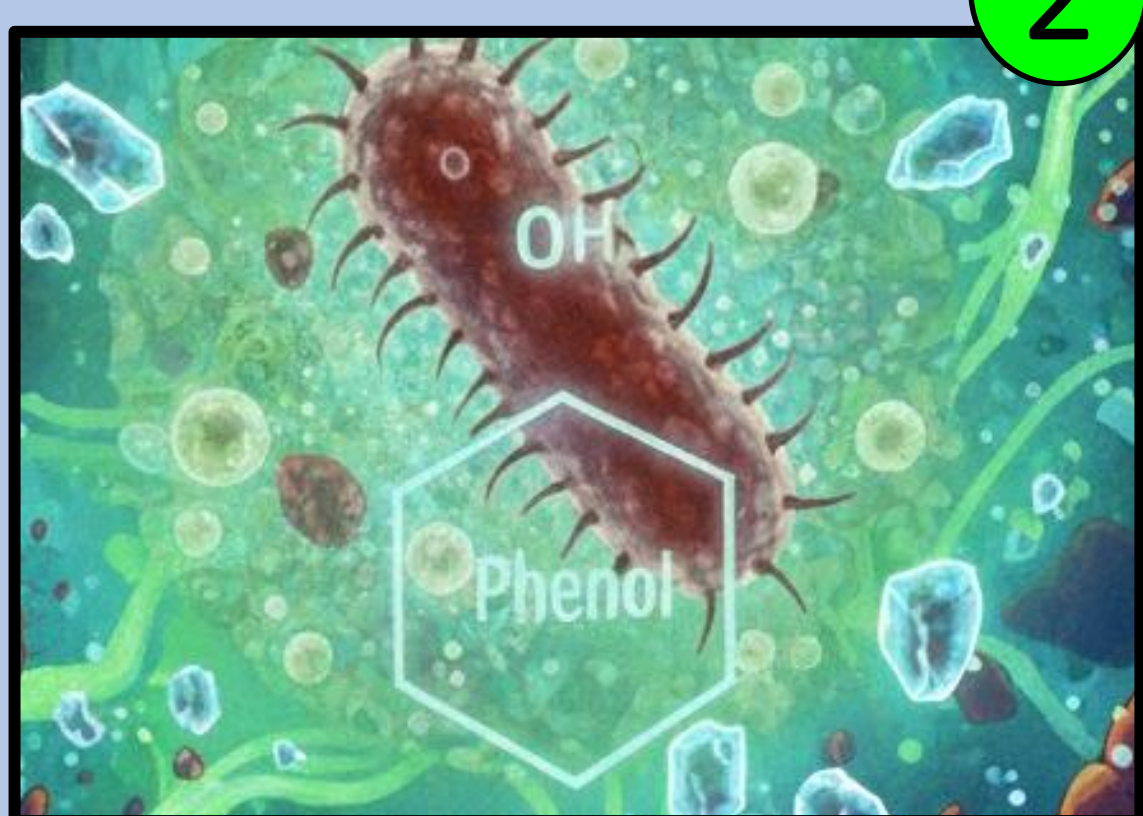

Mycobacterium leprae and *Mycobacterium tuberculosis*. Used with "1%" card to neutralize attacking bacteria

Biphenols (hexachlorophene) 1

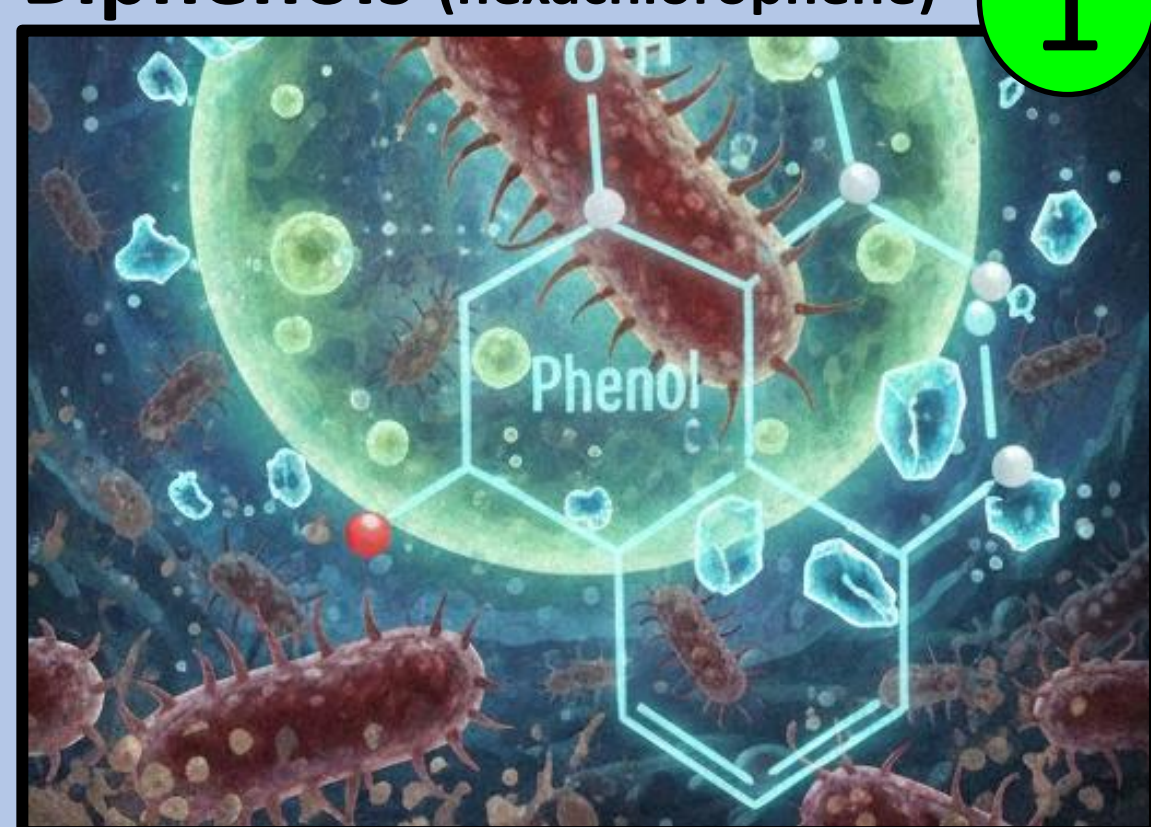

Gram-positive (*Streptococcus* and *Staphylococcus*). Kills targeted bacteria on attack

Triclosan 2

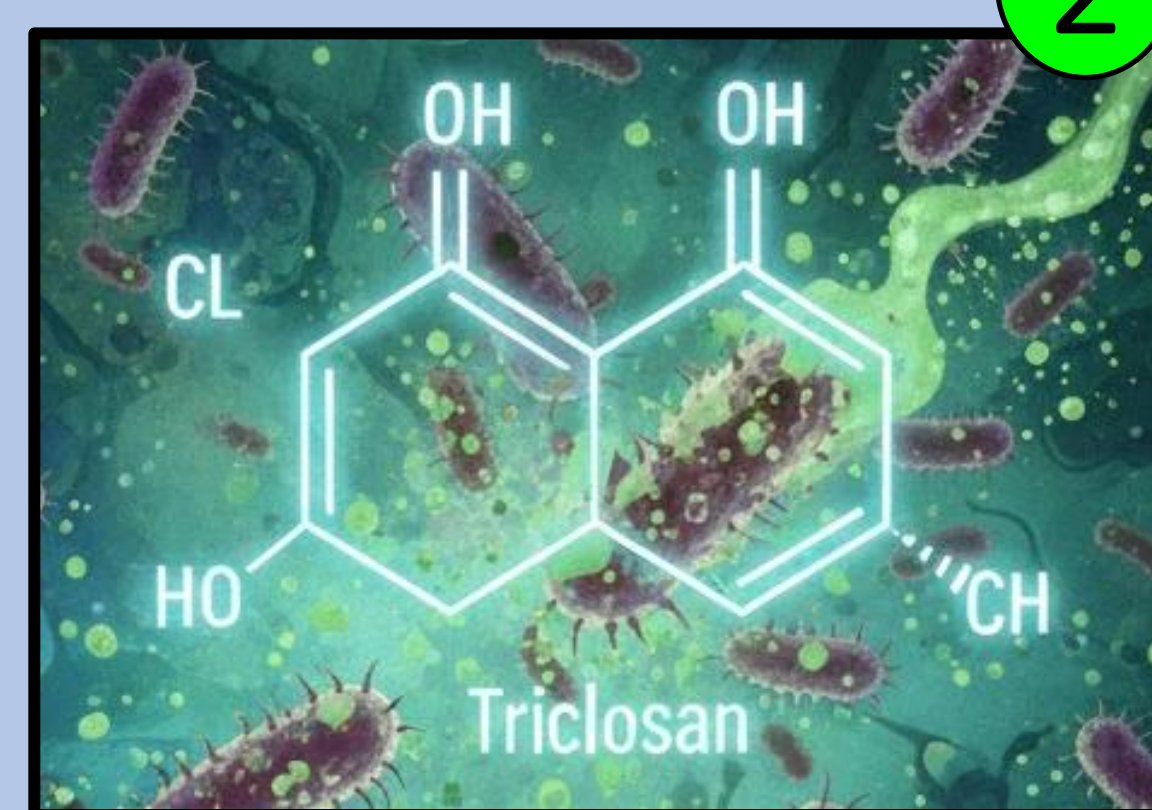

Gram-positive bacteria. Prevents attack for 2 rounds.

Halogens (iodine) 2

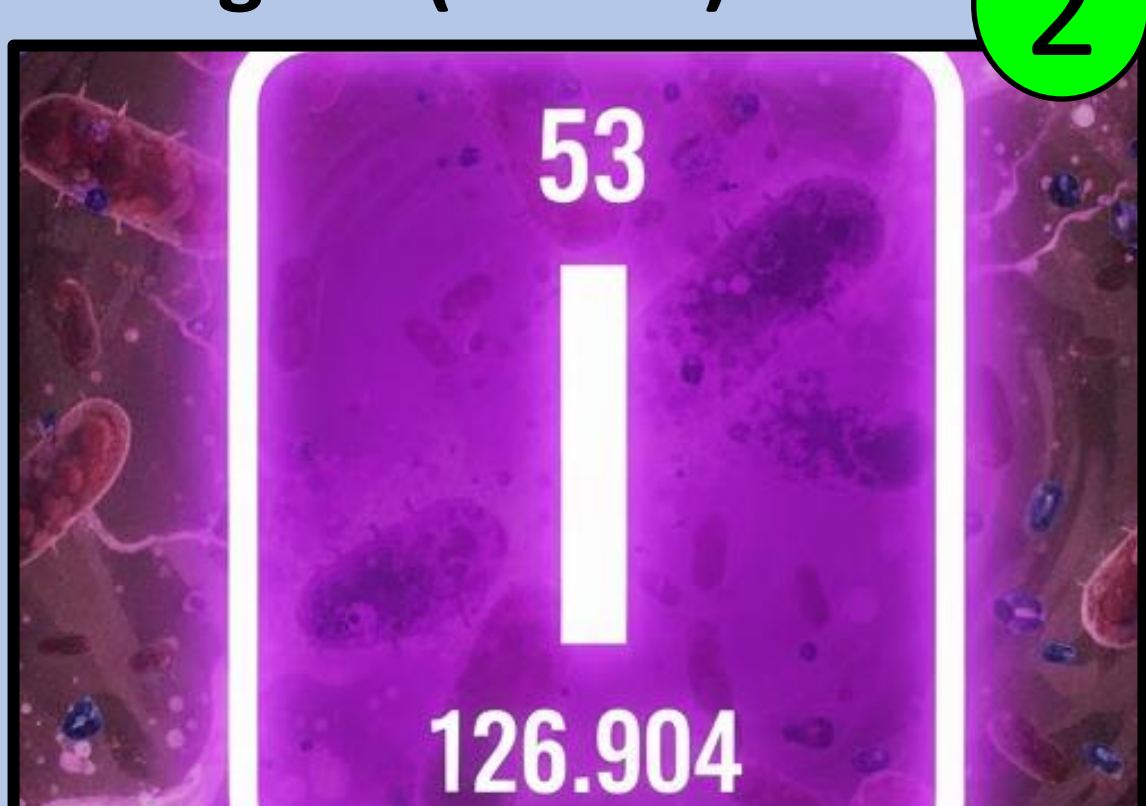

Open wound / Deep wound pathogens. Prevents pathogen entry for 1 round.

Healing ointment 1

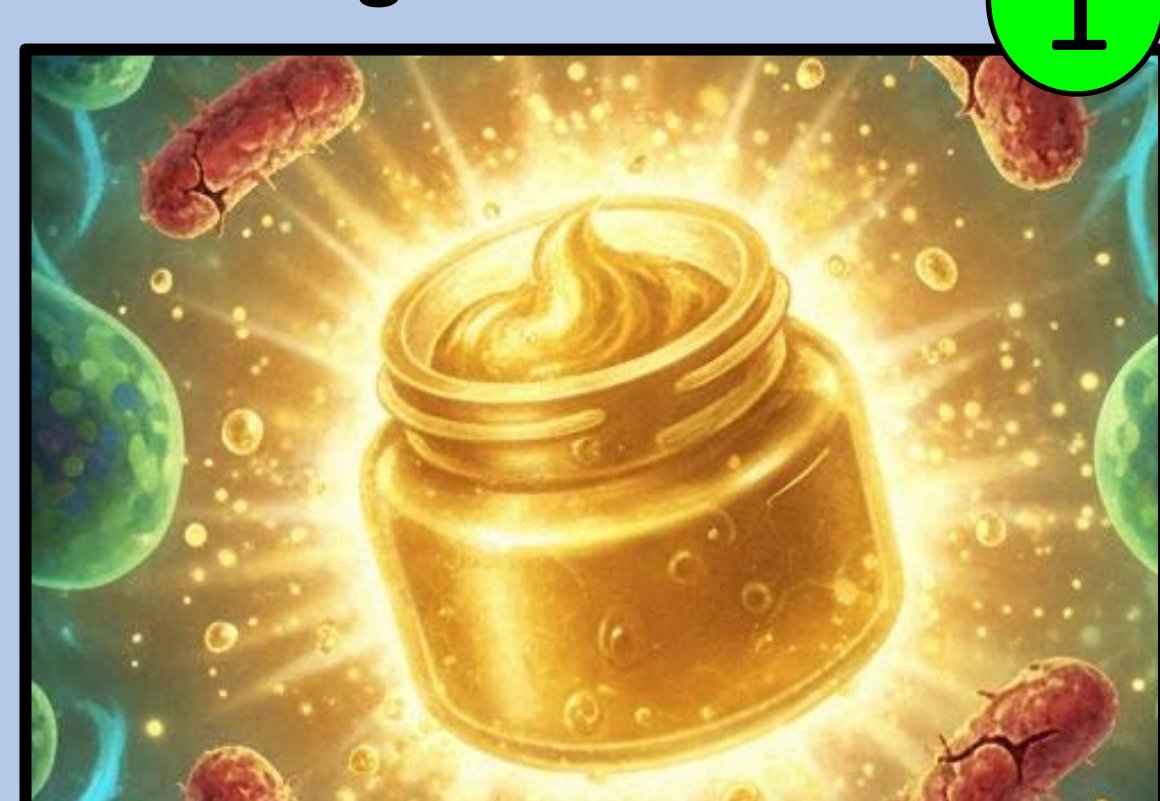

Promotes recovery; assists immune defense

Platelets 1

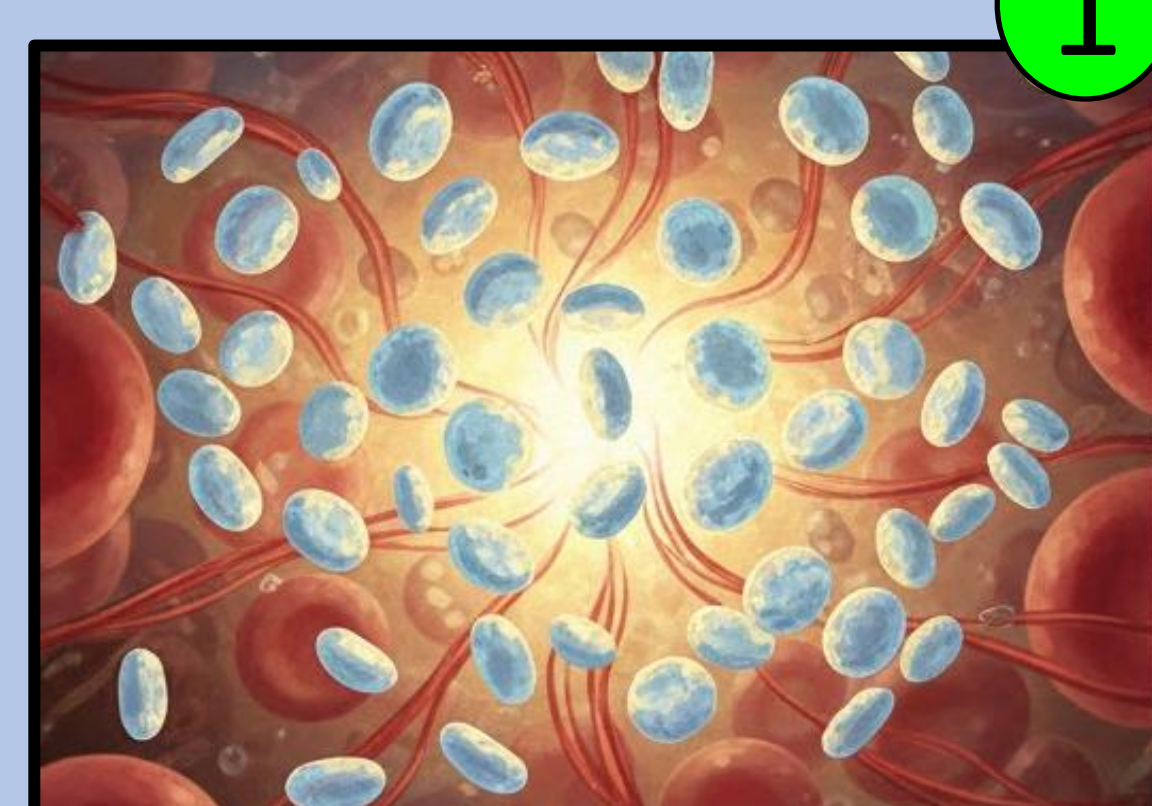

Supports defense against attacking bacteria

Chlorine 3

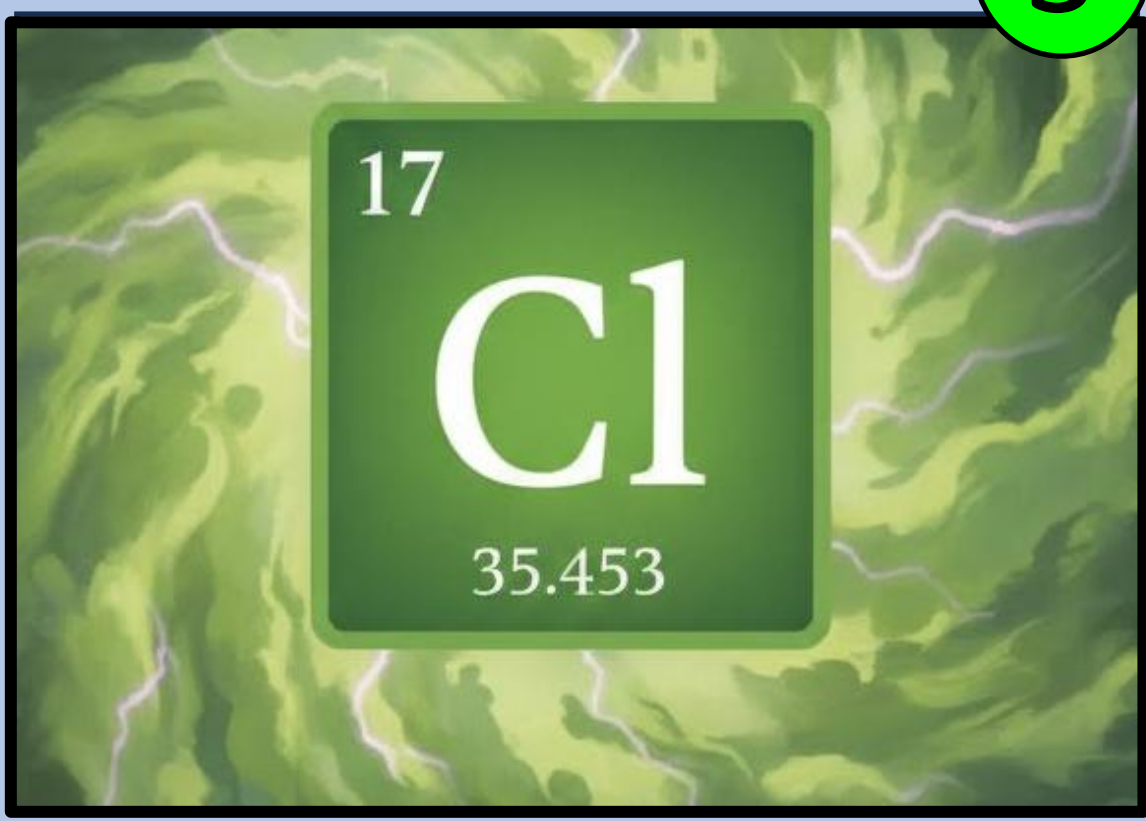

Water-borne bacteria. Protects individual for 2 rounds when combined with “Water” card.

Alcohol 70% 2

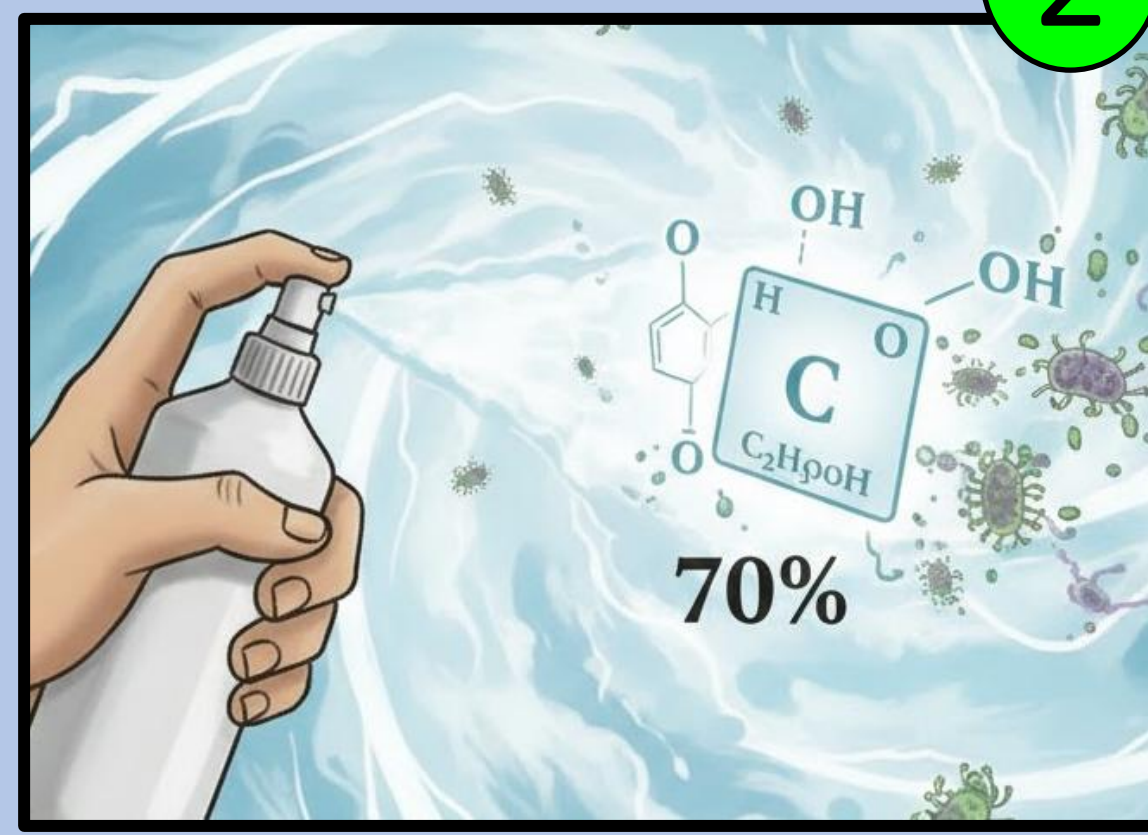

All bacteria. Protects individual for 1 round.

Sodium nitrite 2

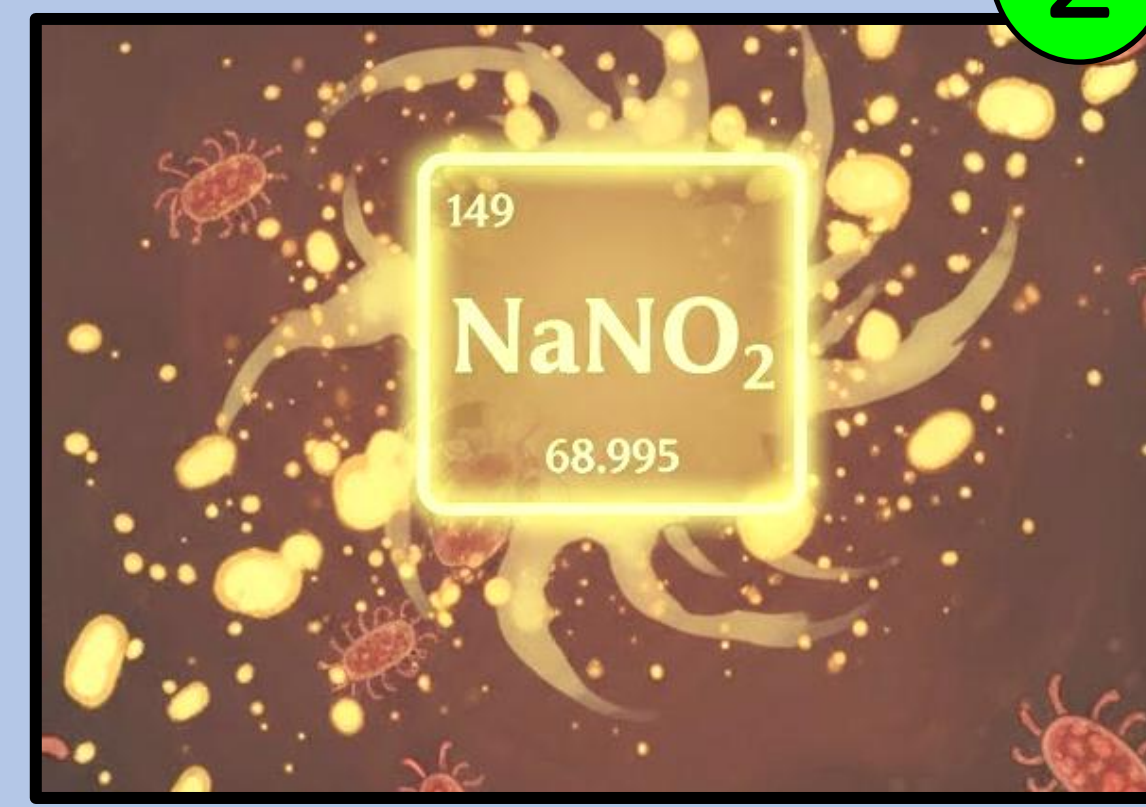

Bacteria in “Contaminated meat”. Neutralizes attack; bacteria return to deck.

Research and treatment 1

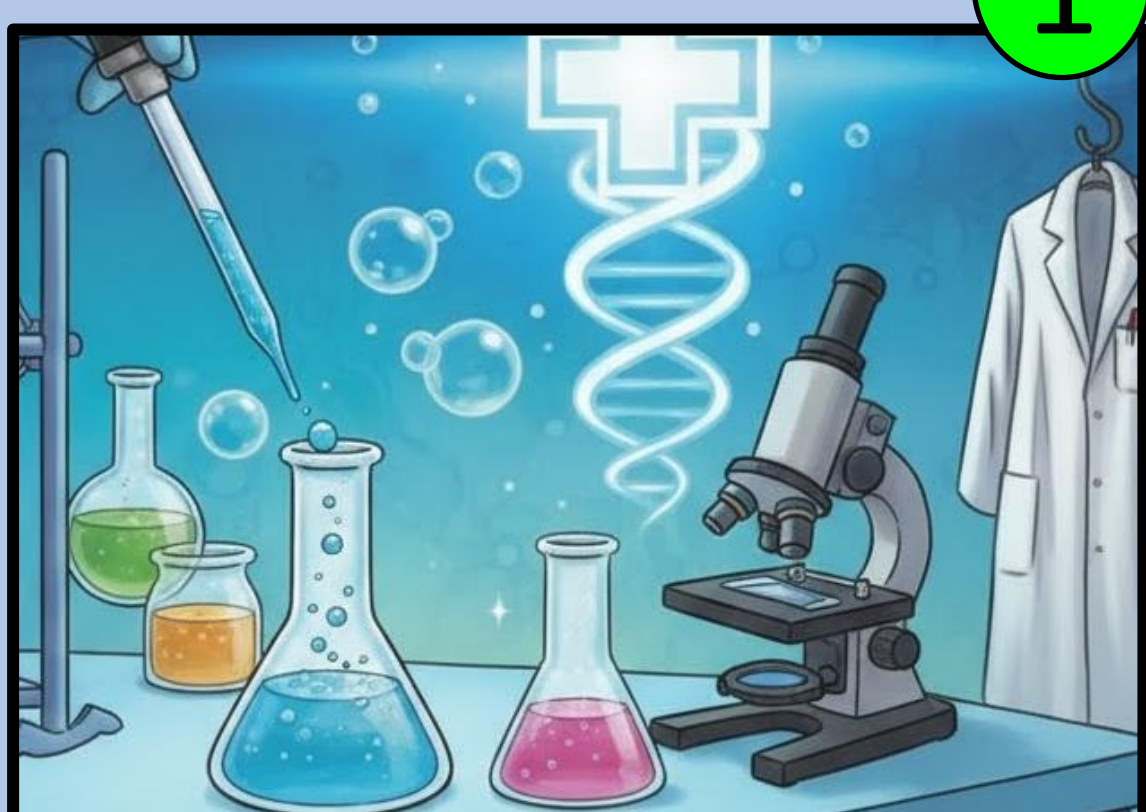

Attacking bacteria. Paralyzes attack for 2 rounds.

Quaternary ammonium compounds 2

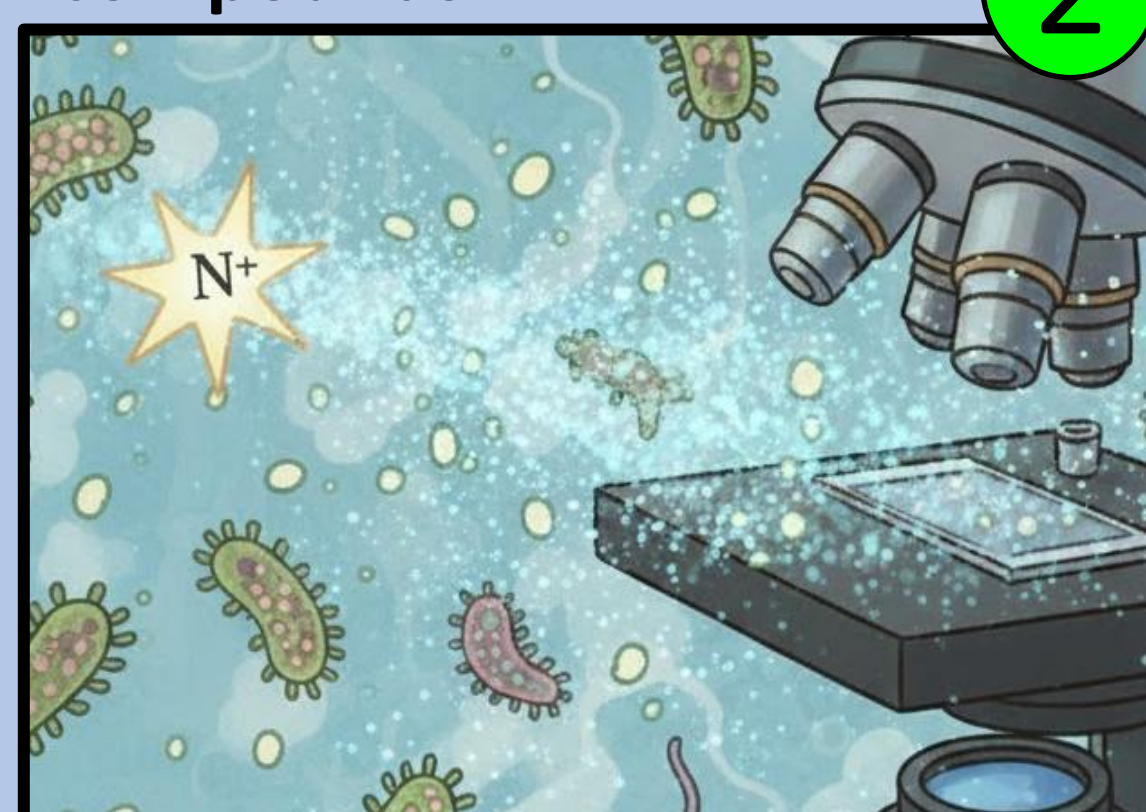

Throat infection bacteria (except *Pseudomonas*). Neutralizes attacks for 2 rounds; less effective in organic matter.

Slow freezing 1

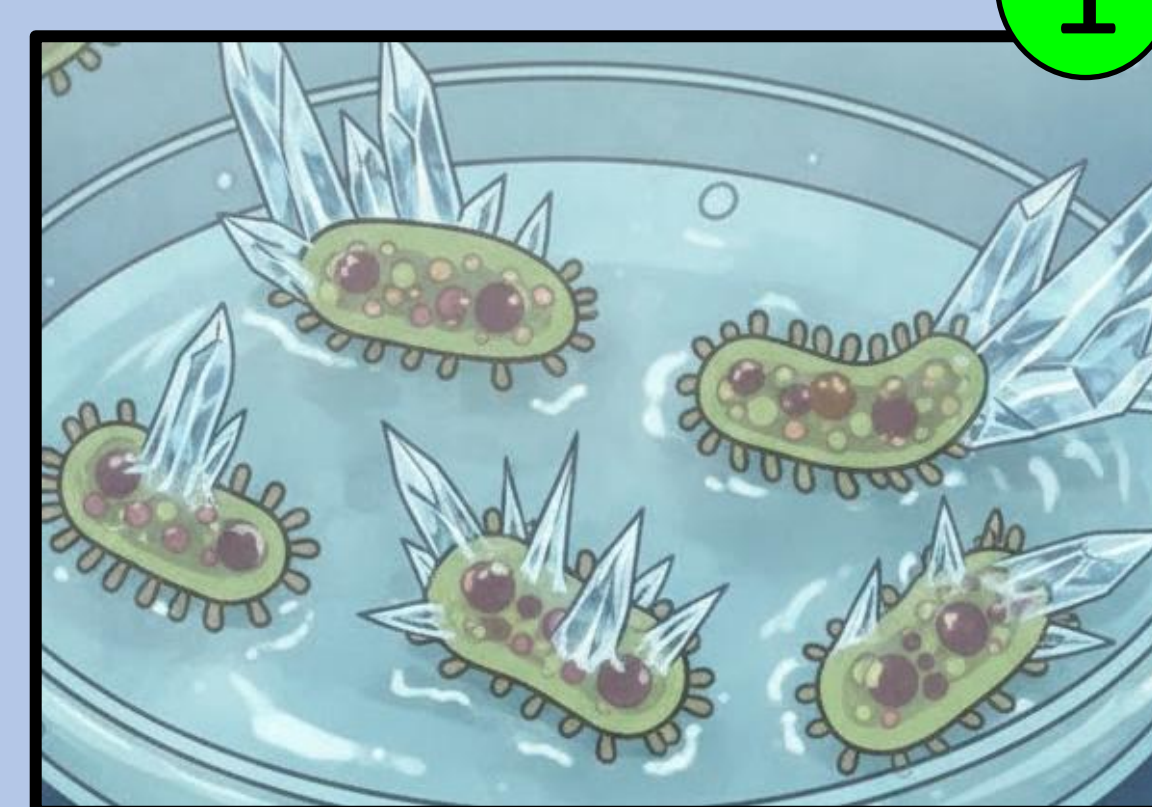

Attacking bacteria. Kills bacteria after 3 rounds via crystal formation.

Fast freezing 1

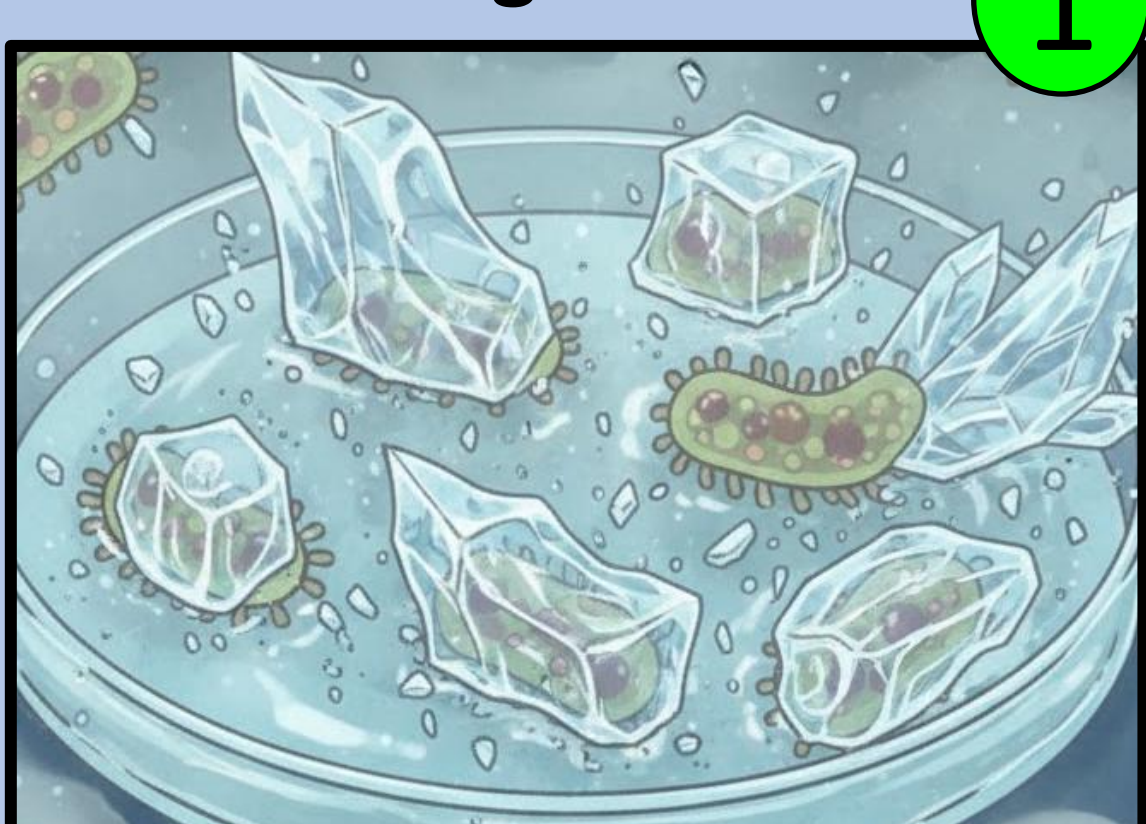

Attacking bacteria. Paralyzes attack for 2 rounds; does not kill.

Autoclave 2

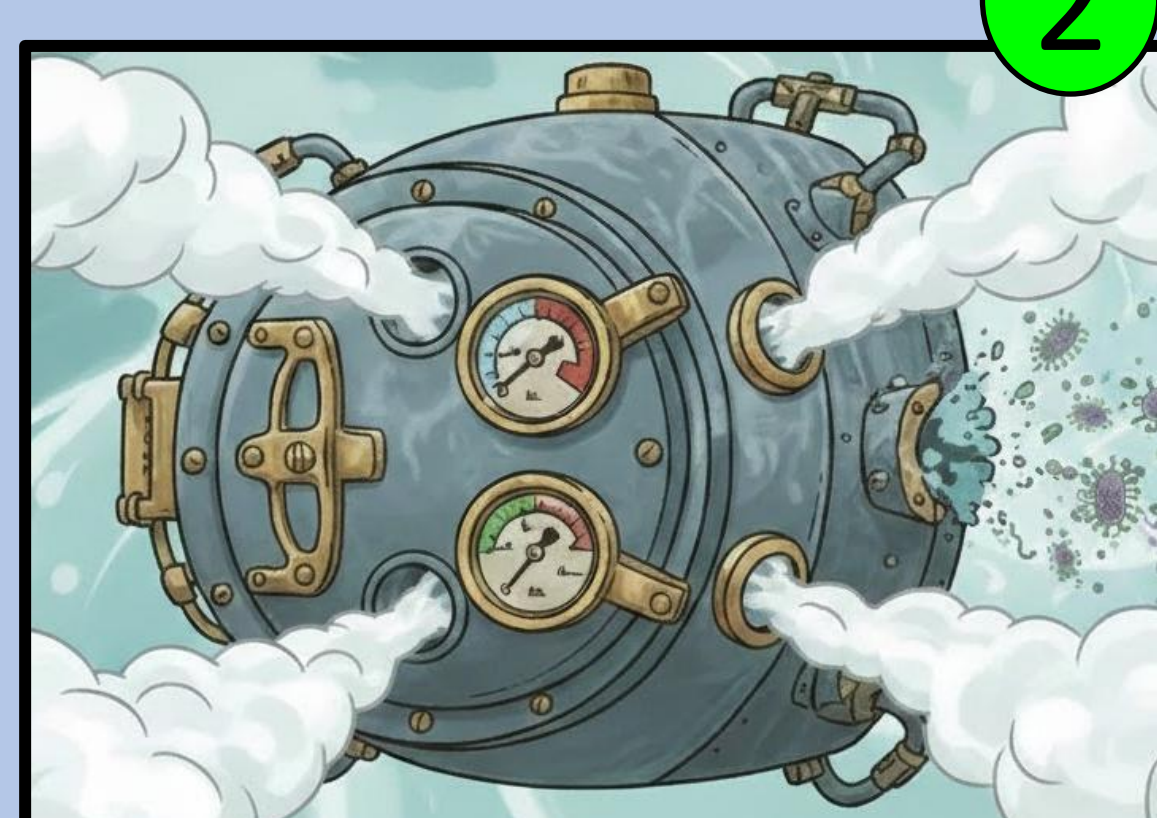

All attacking bacteria. Kills all bacteria; requires “15 min” card.

Pasteurization 5

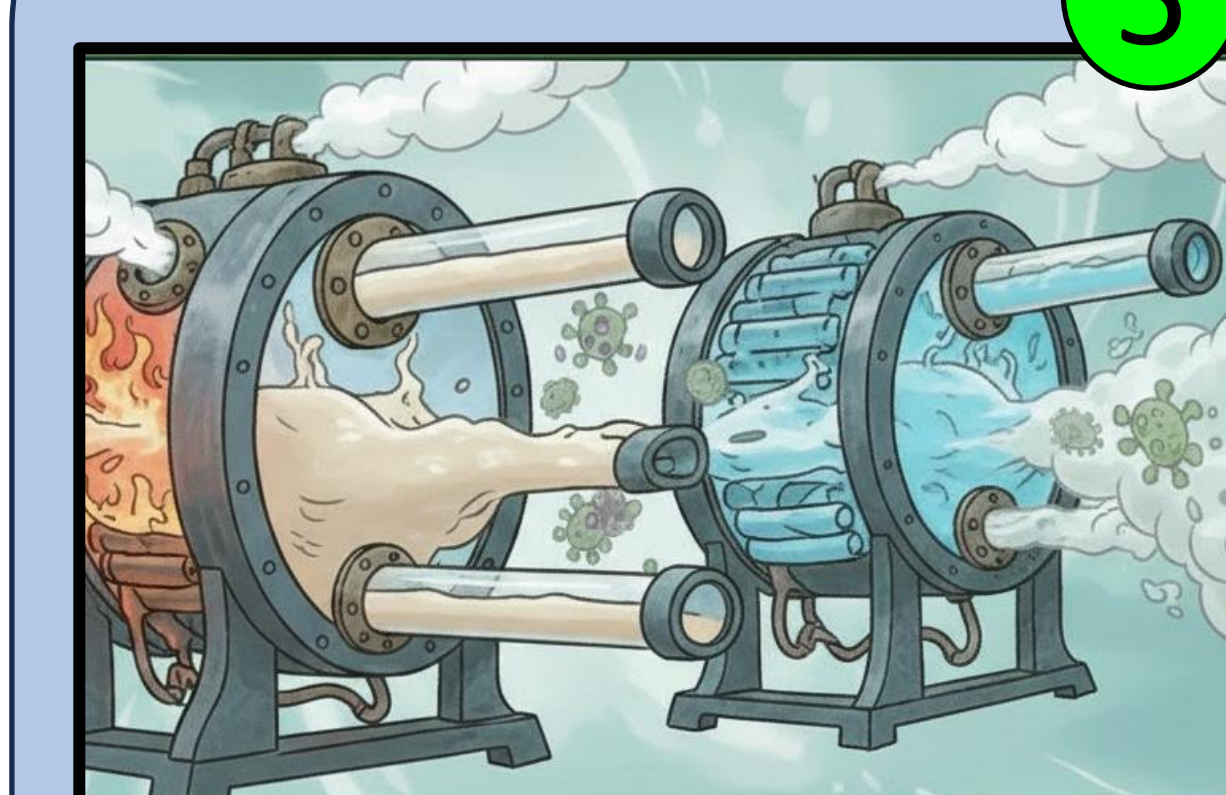

All attacking bacteria. Kills bacteria from attacking opponent.

Vitamin C 2

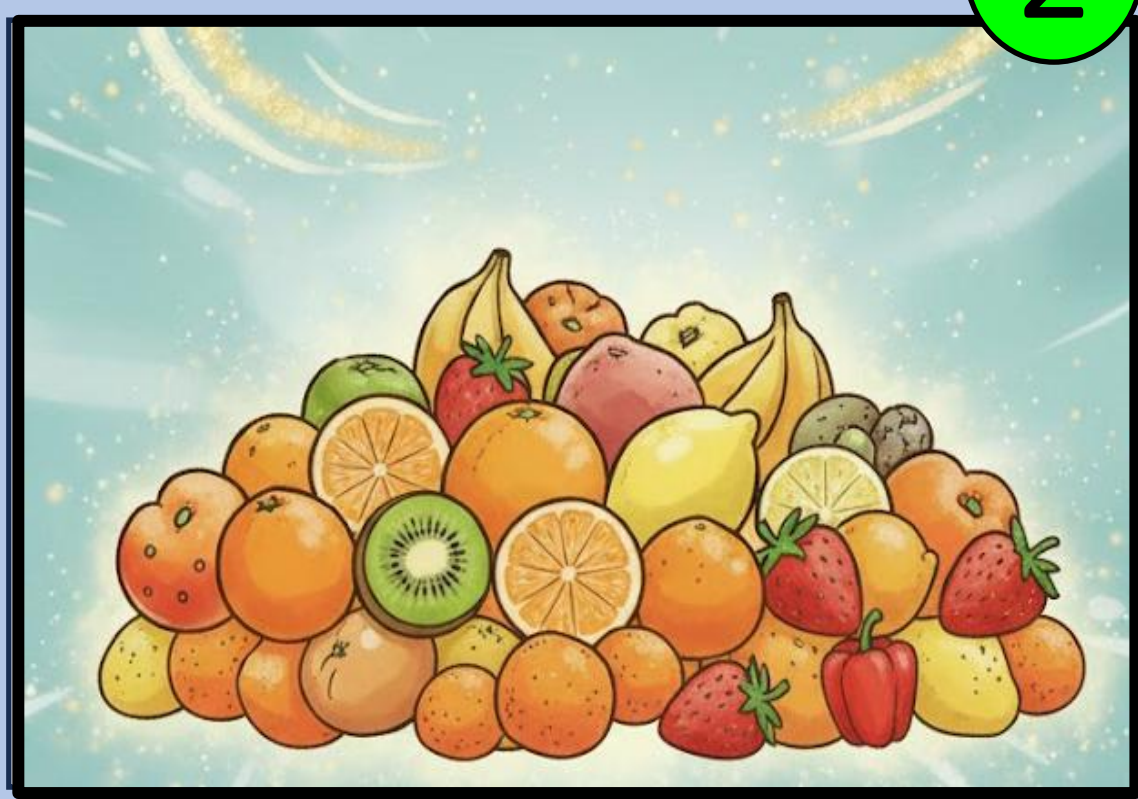

Macrophages / Neutrophils. Increases destructive power against 2 bacteria.

Neutrophil 2

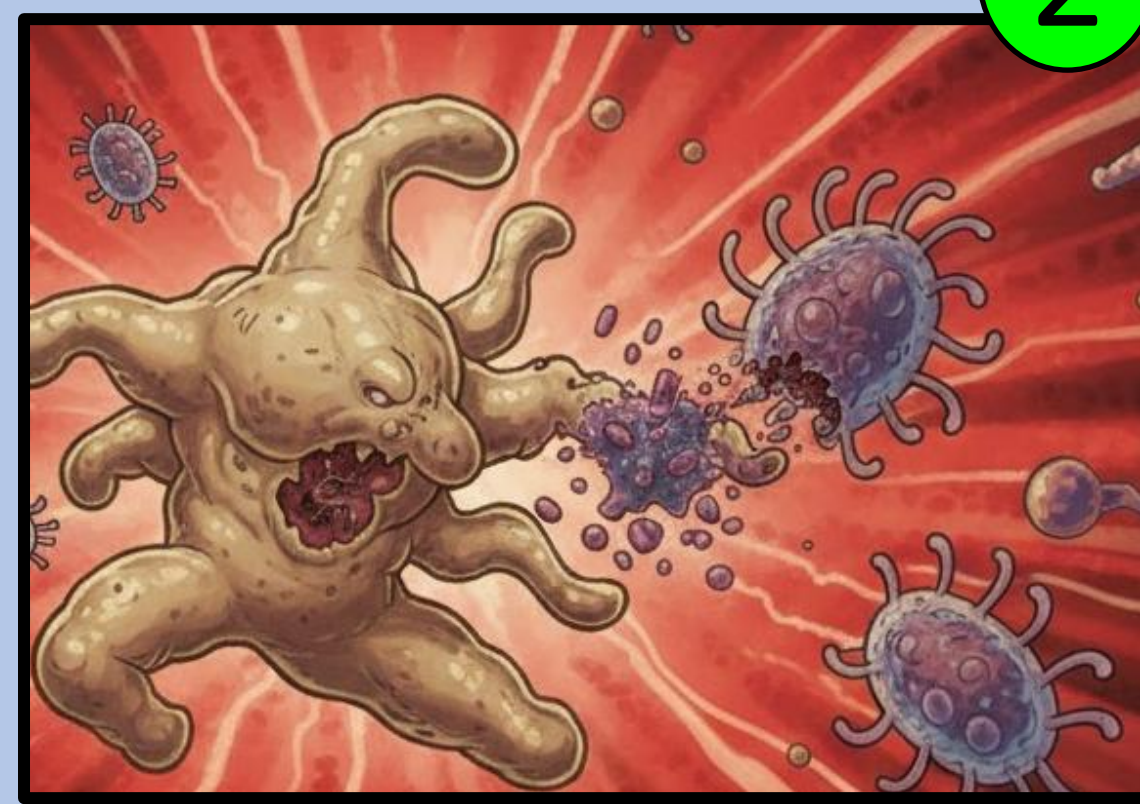

1 bacterium in attack. Kills bacteria; enhanced with Vitamin C / T4 lymphocytes.

Macrophage 4

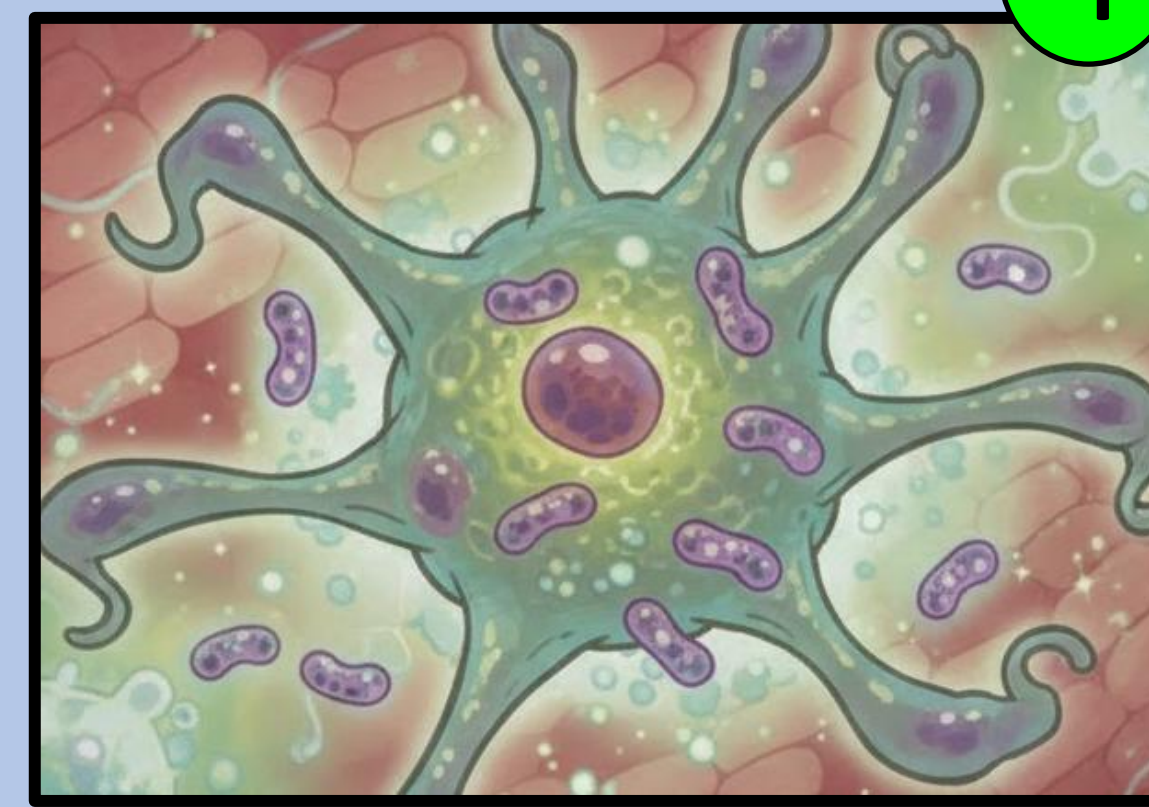

1 bacterium in attack. Kills bacteria; enhanced with Vitamin C / T4 lymphocytes.

Aniquilating Serpent 2

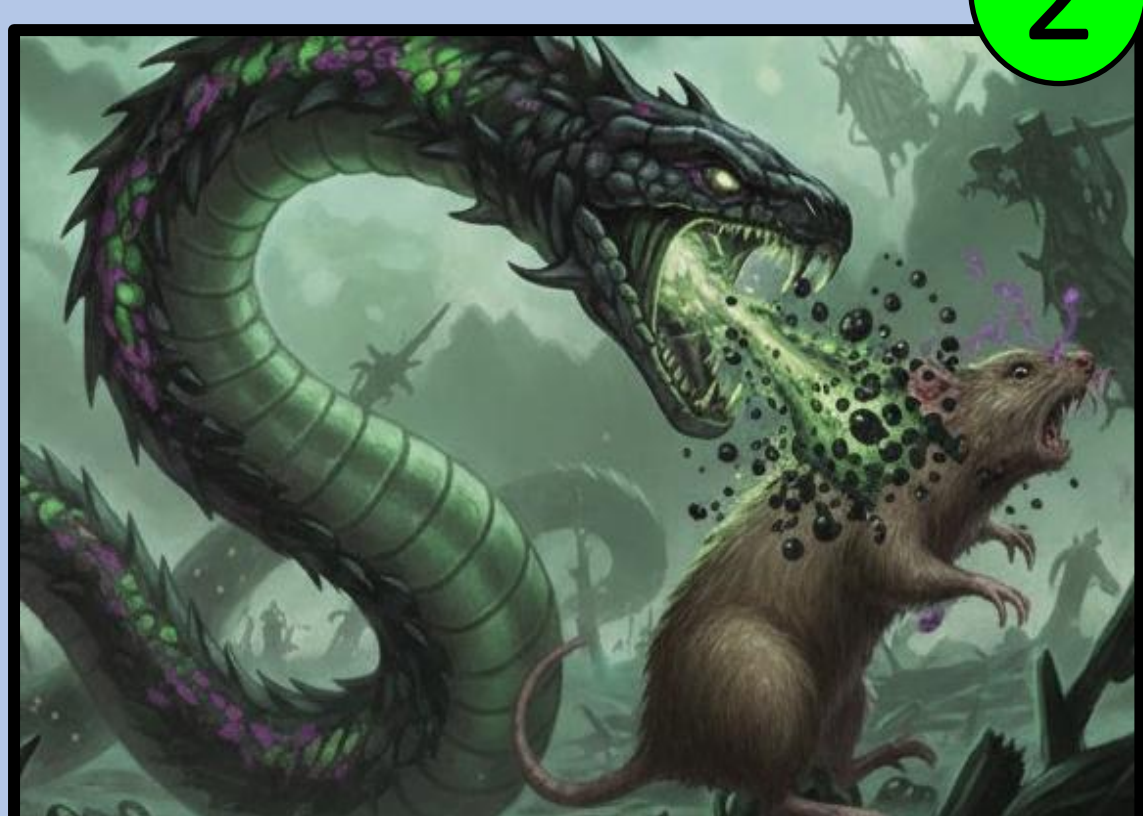

Rats transmitting plague. Removes rat cards from play; returns to deck.

Poison against lice and ticks 3

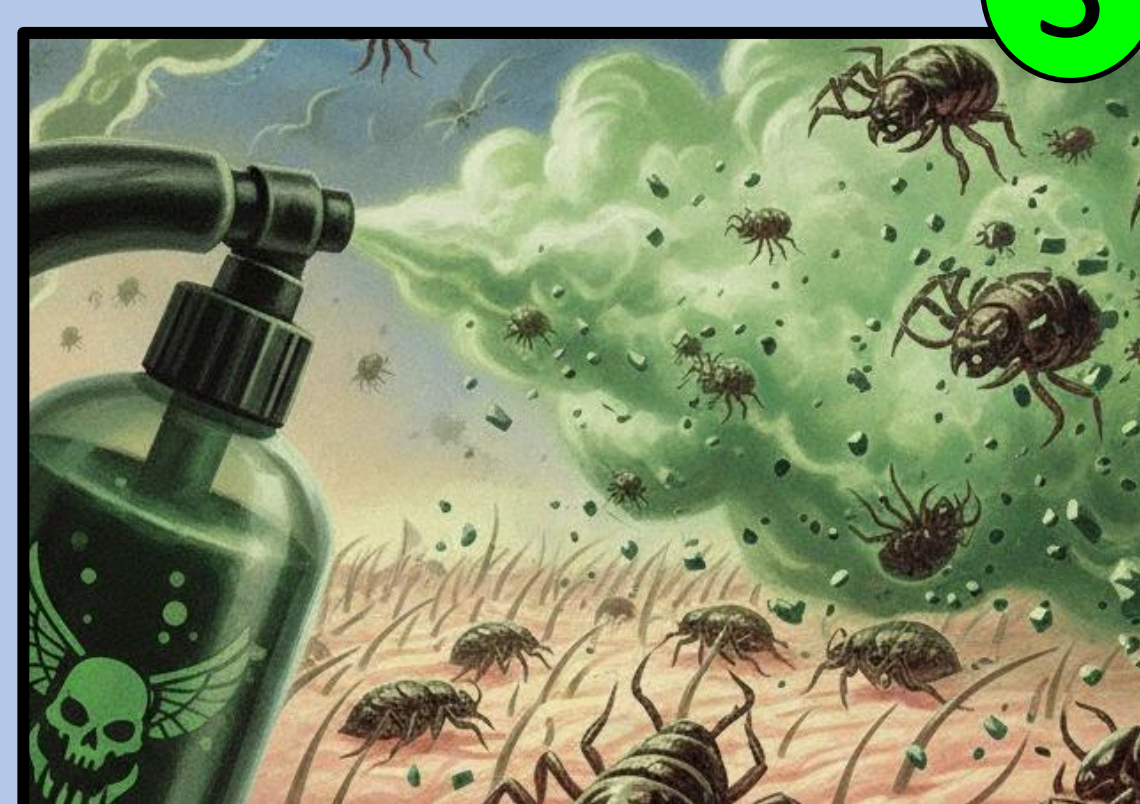

Lice / ticks. Eliminates vector cards; returns them to deck.

Care in sexual relations 1

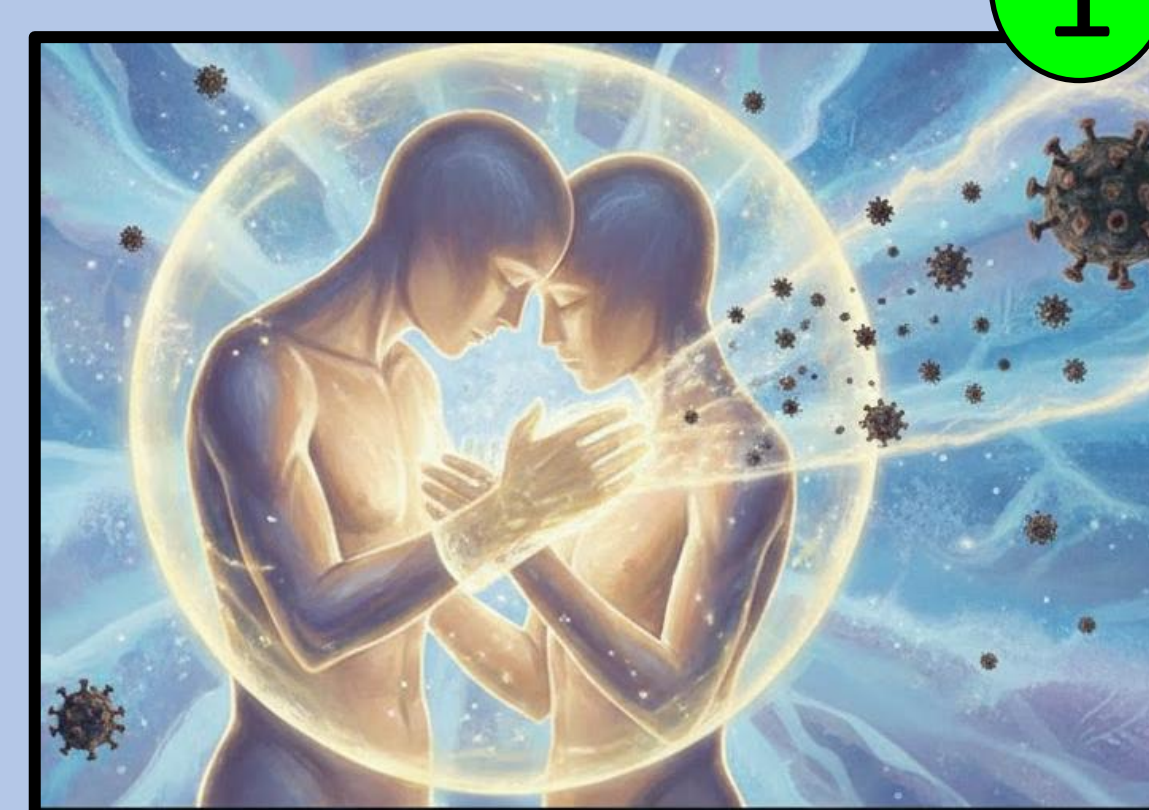

Sexually transmitted pathogens. Protects for 2 rounds.

Decomposer bacteria 2

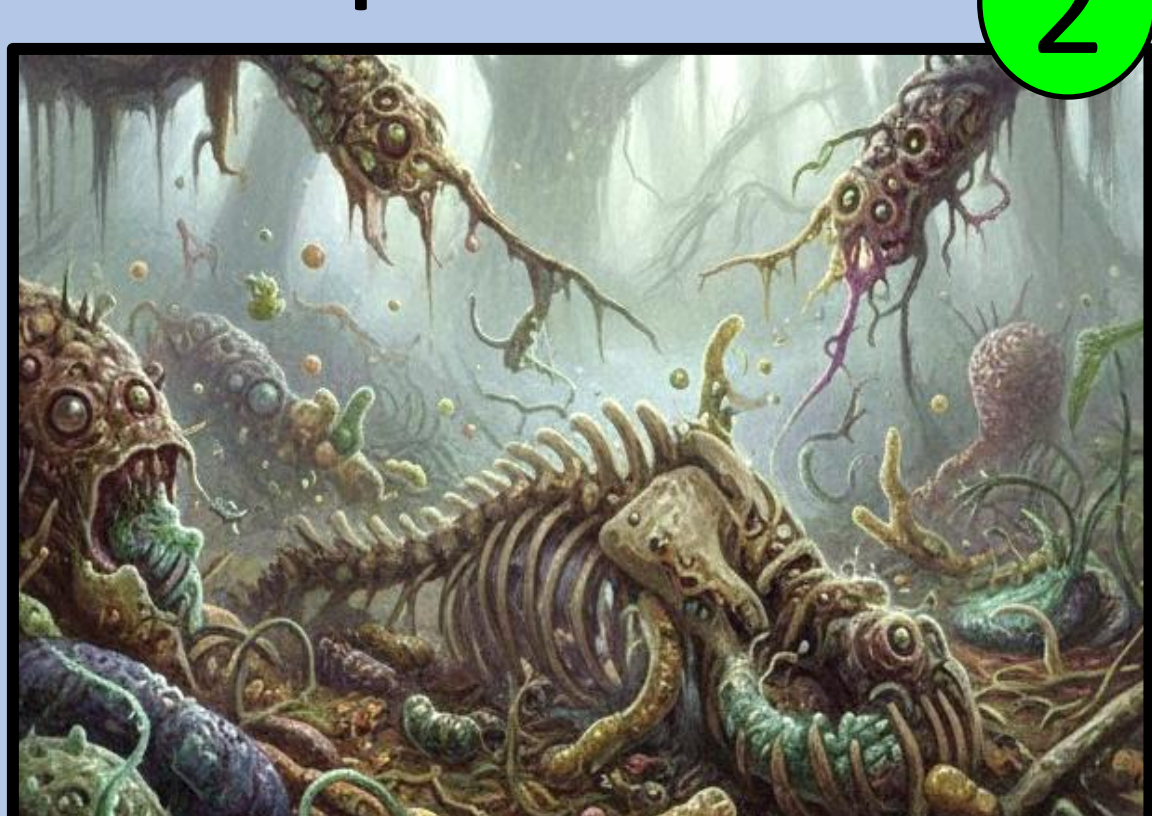

Food-borne bacteria. Removes multiplying bacteria from table.

Health surveillance 0

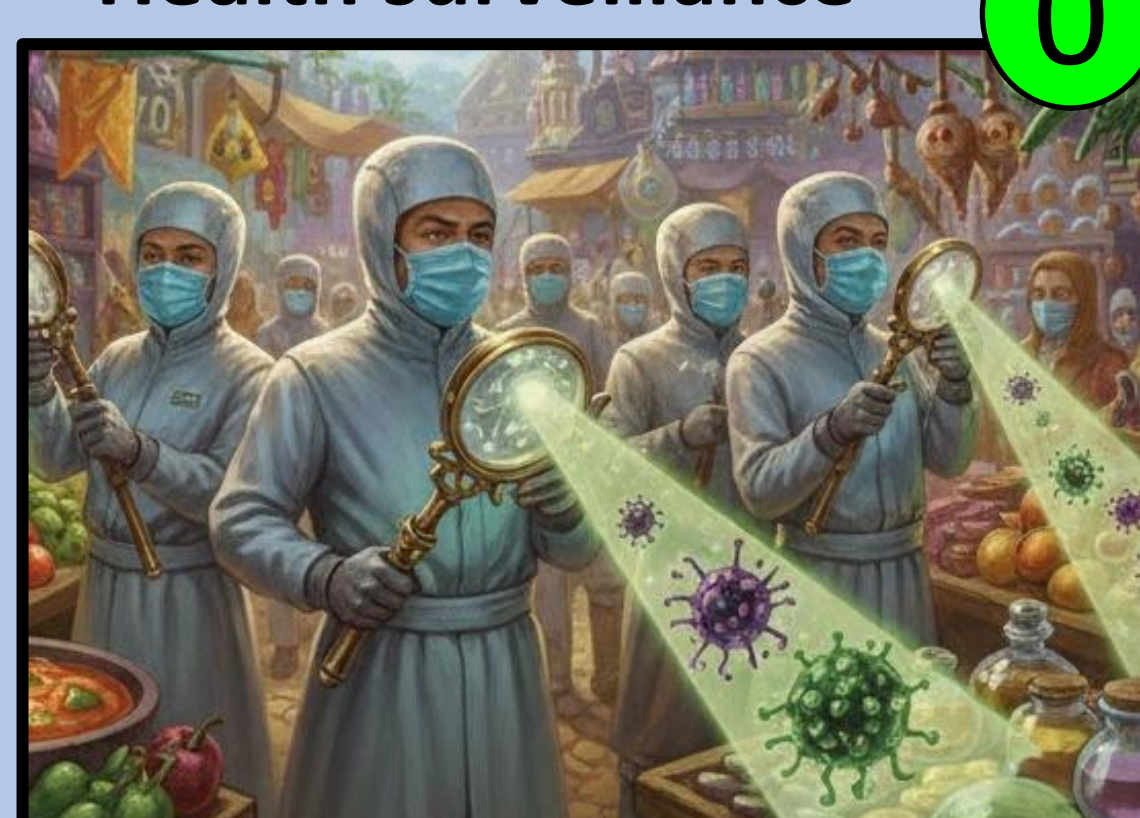

Food-borne bacteria. Eliminates pathogens causing foodborne illness.

Physical exercises 2

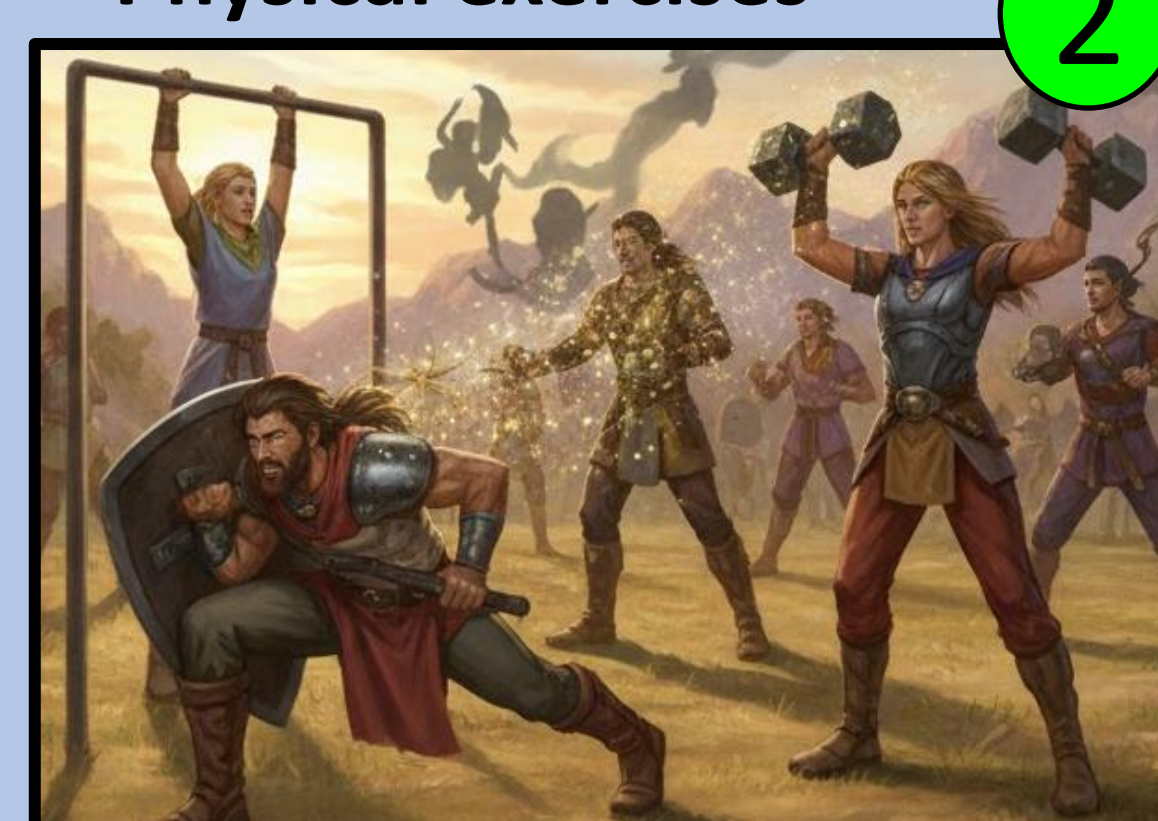

1 bacterium. Increases immune cell attack power.

Keep calm 1

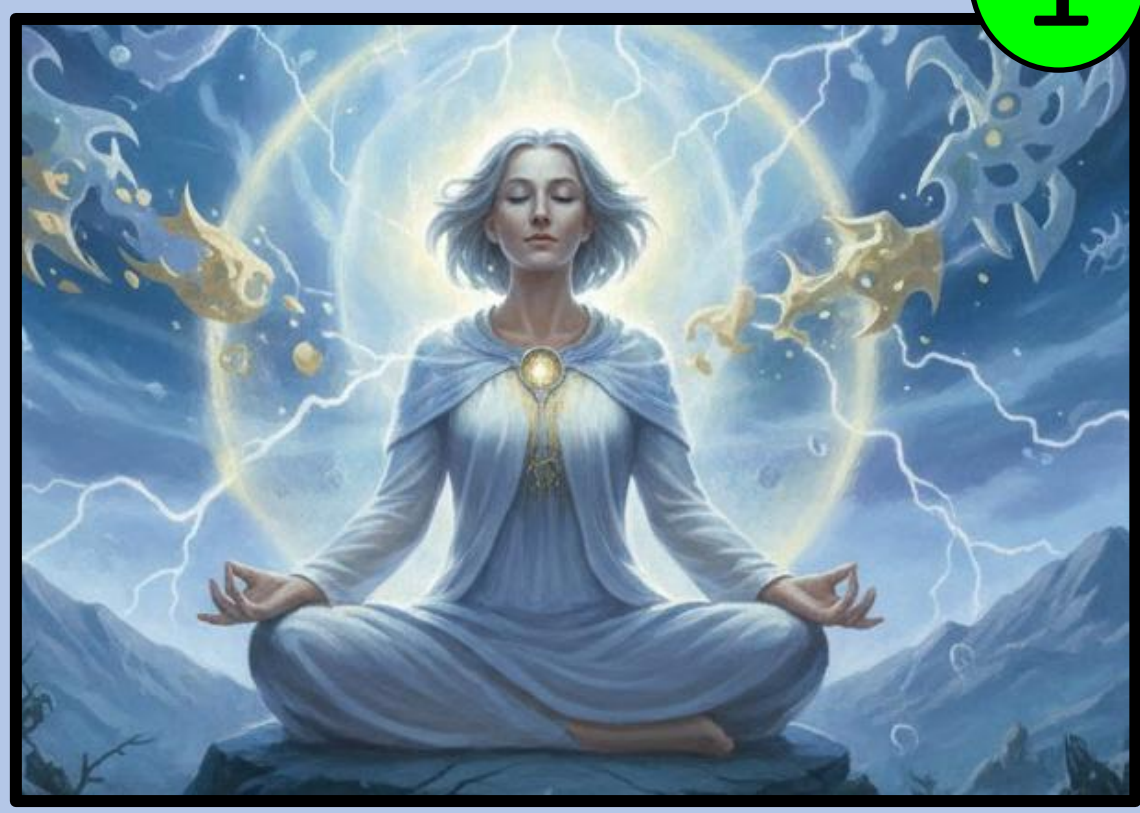

Stress card. Neutralizes "Stress" effect.

Bedellovibrio 1

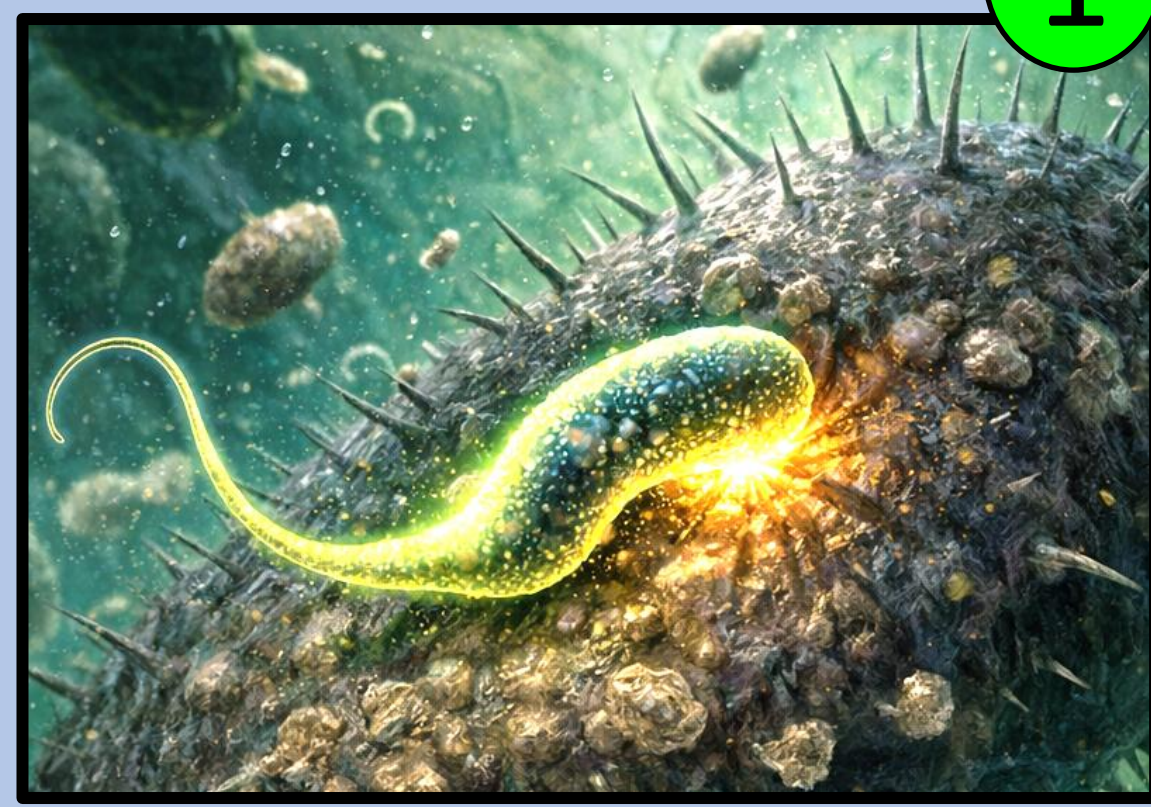

1 Gram-negative bacterium. Kills targeted bacterium.

Medical prescription 2

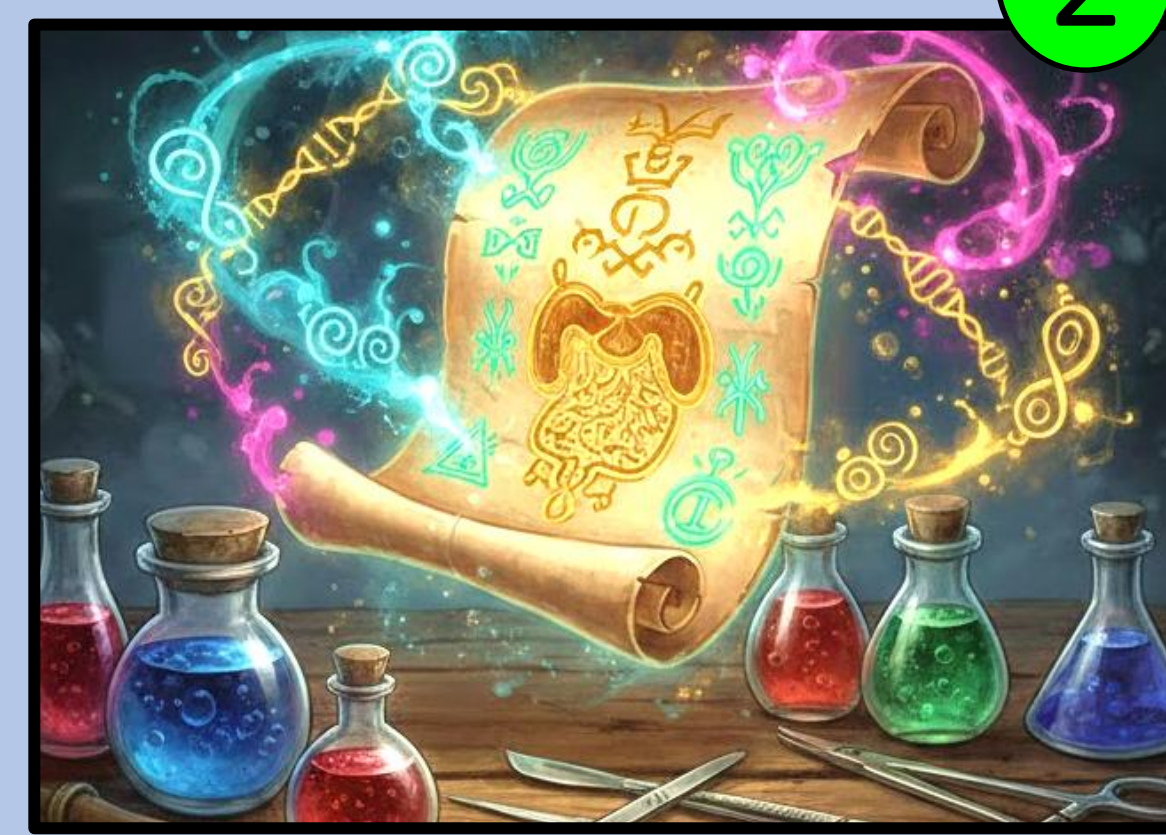

Enhances action of antibiotics cards.

DPT vaccine 0

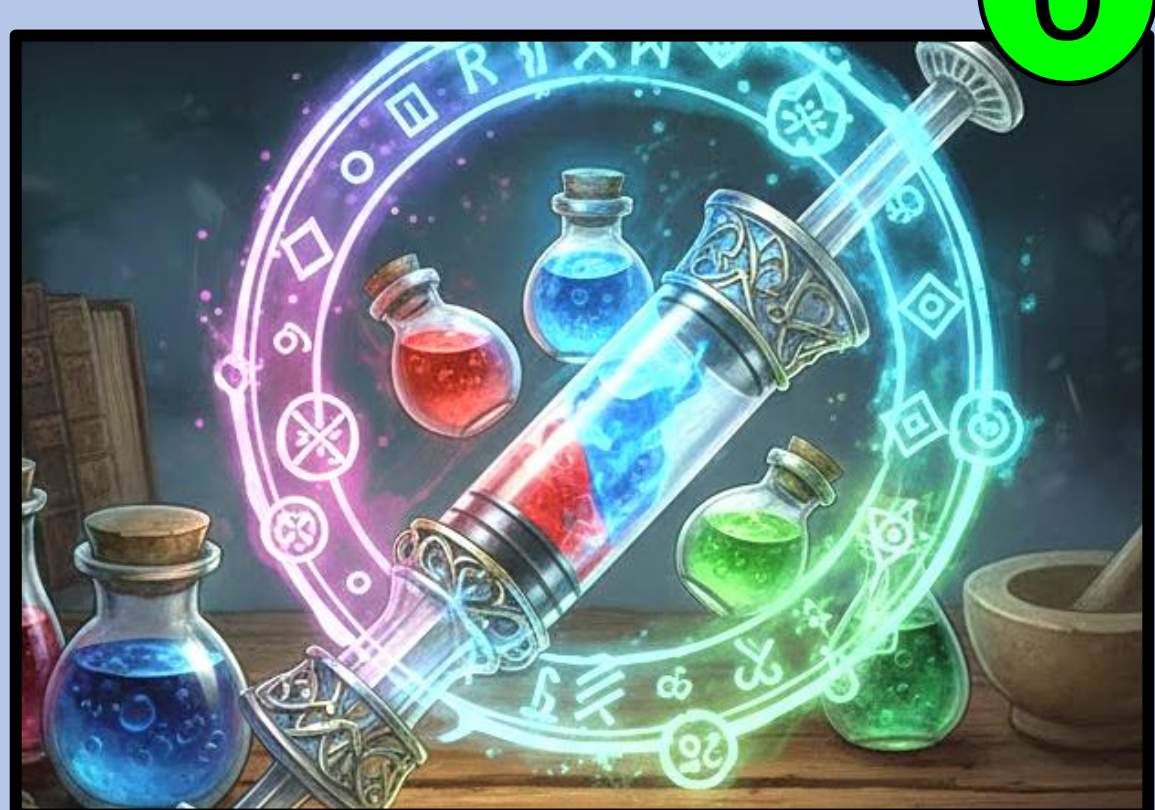

Diphtheria, Pertussis, Tetanus bacteria. Provides immunity to targeted pathogens.

Water 1

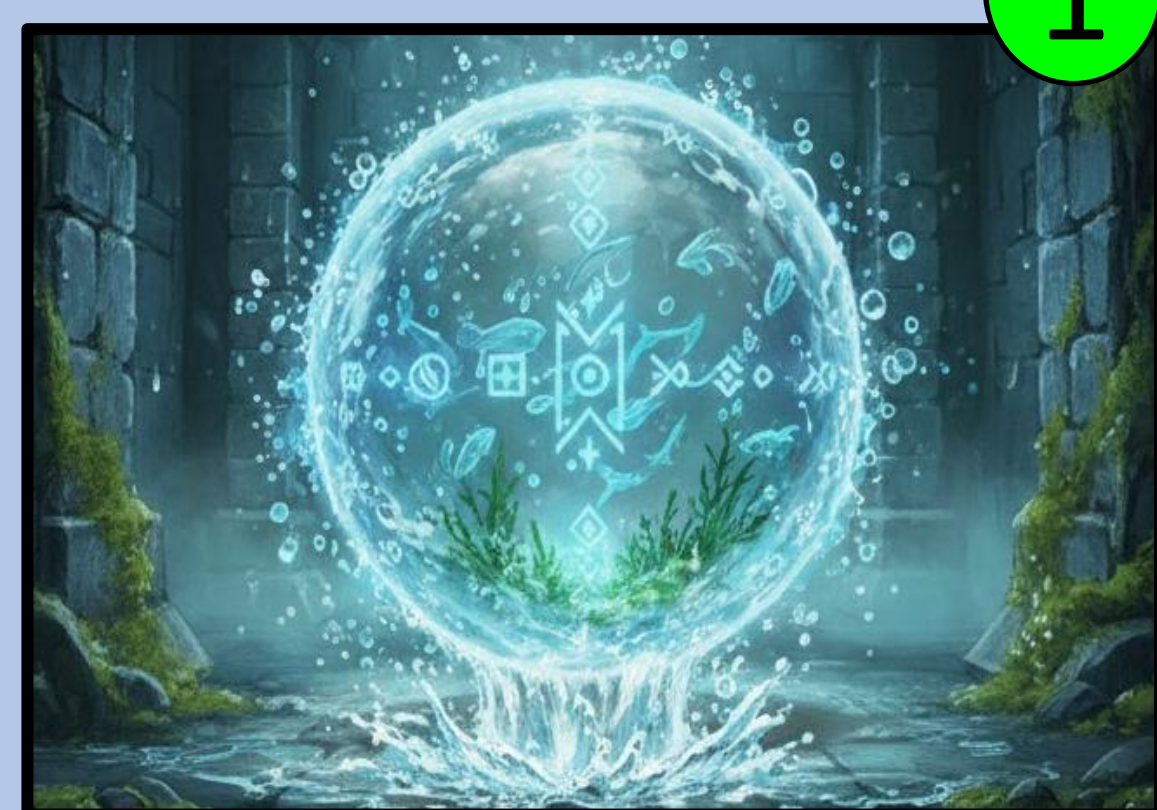

Chlorine card. Activates chlorine effectiveness against bacteria.

Surfactant 4

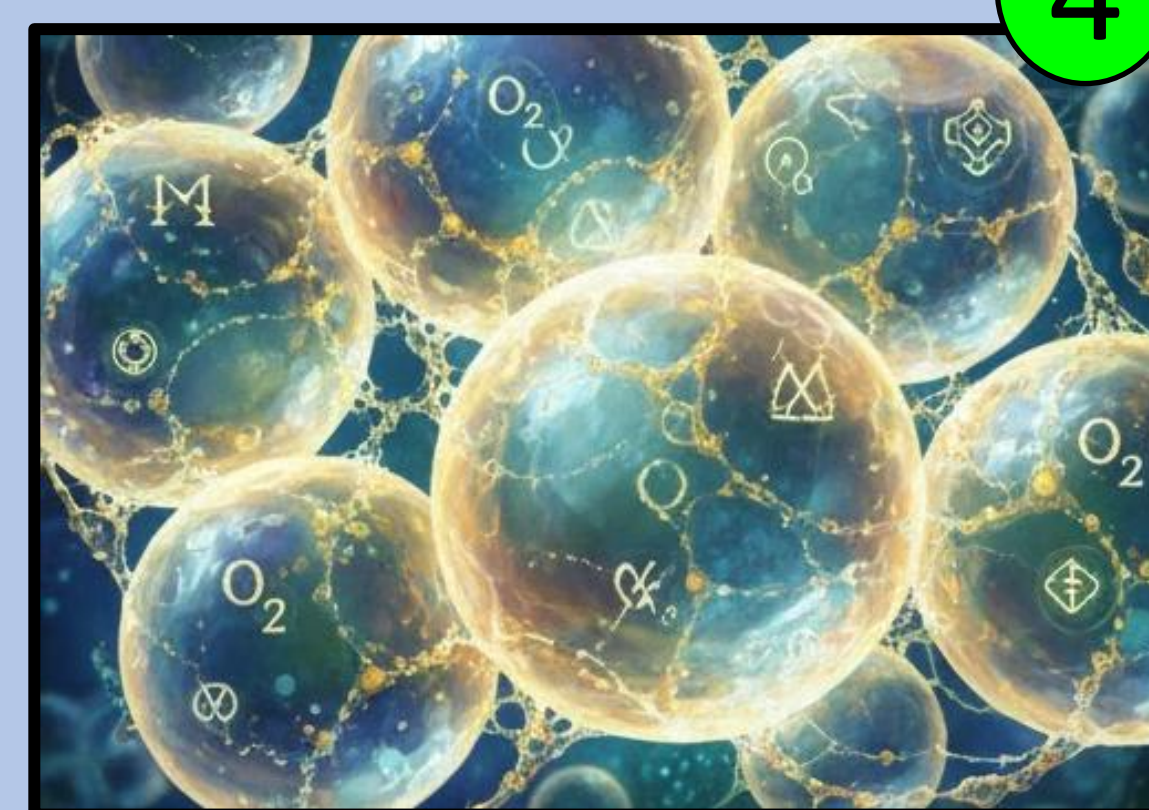

Surface pathogens / biofilms. Reduces pathogen adhesion; blocks attack for 1 round.

Bacterial transformation 2

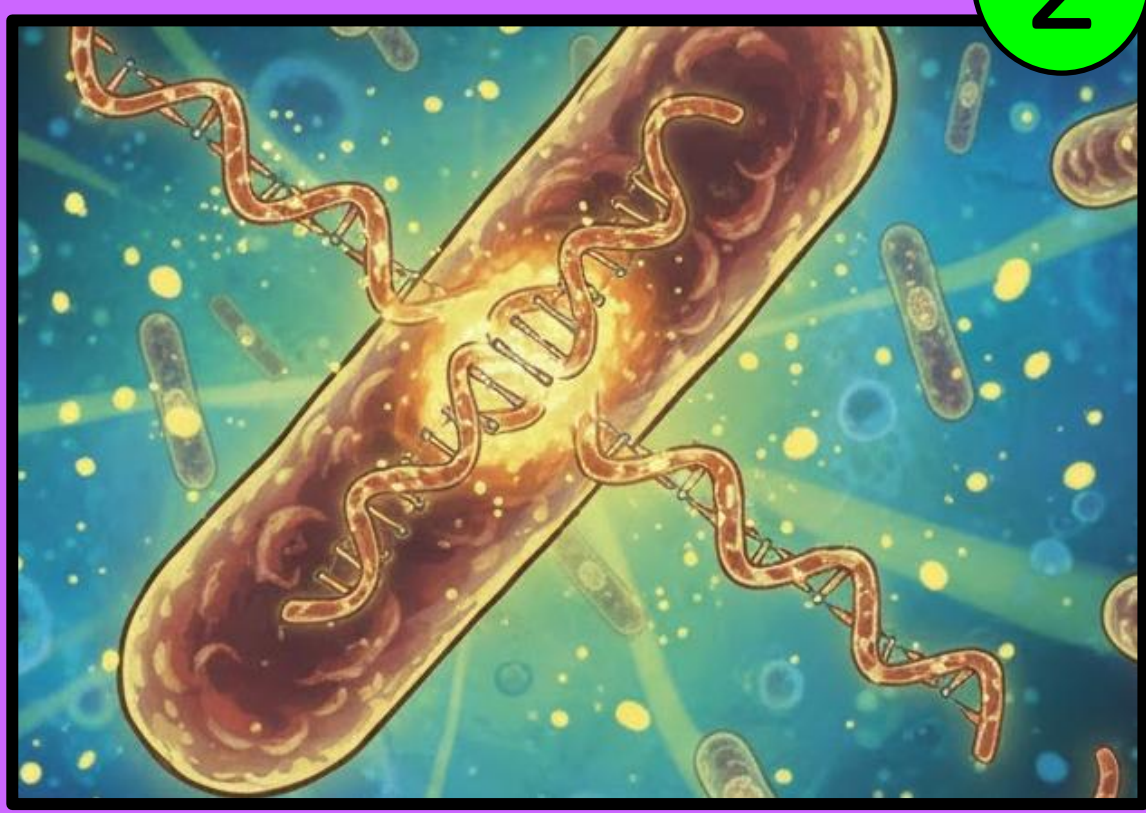

Attacking bacteria. Increases genetic variability; evades antibiotic action.

Bad eating habits 1

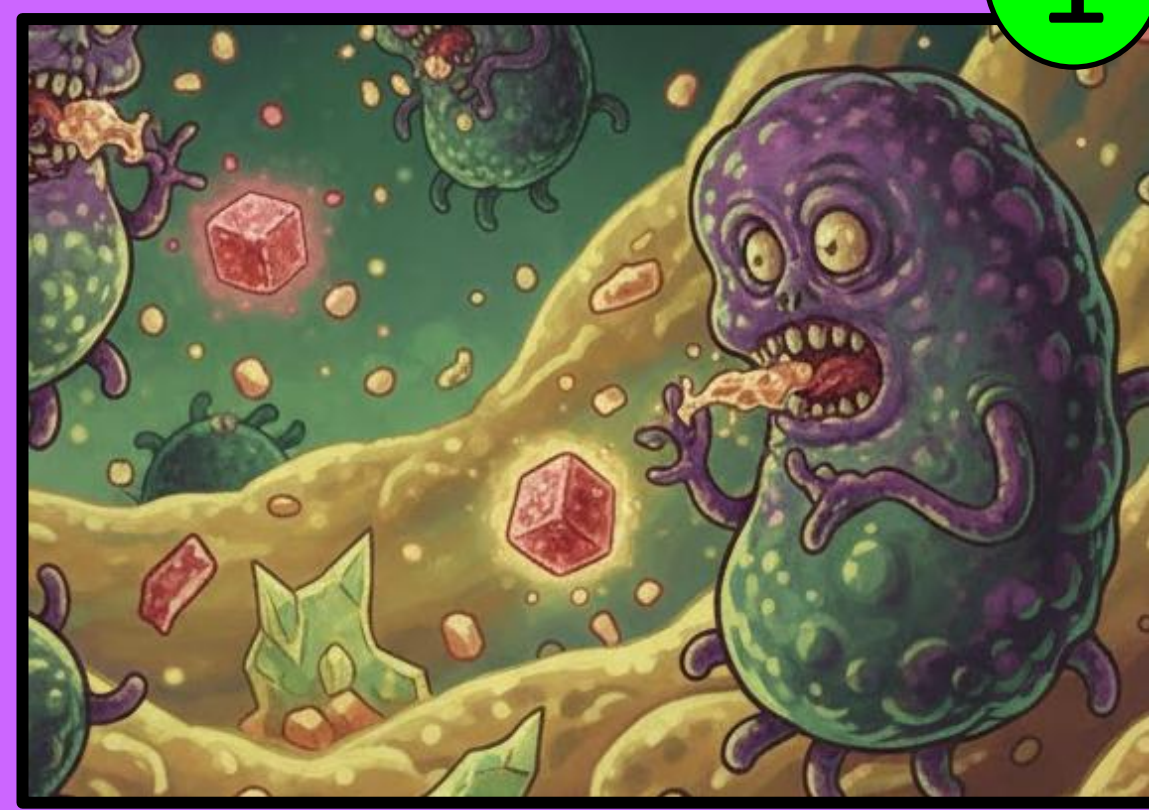

Host immune cells. Reduces macrophage and neutrophil efficiency.

Biological risk 2

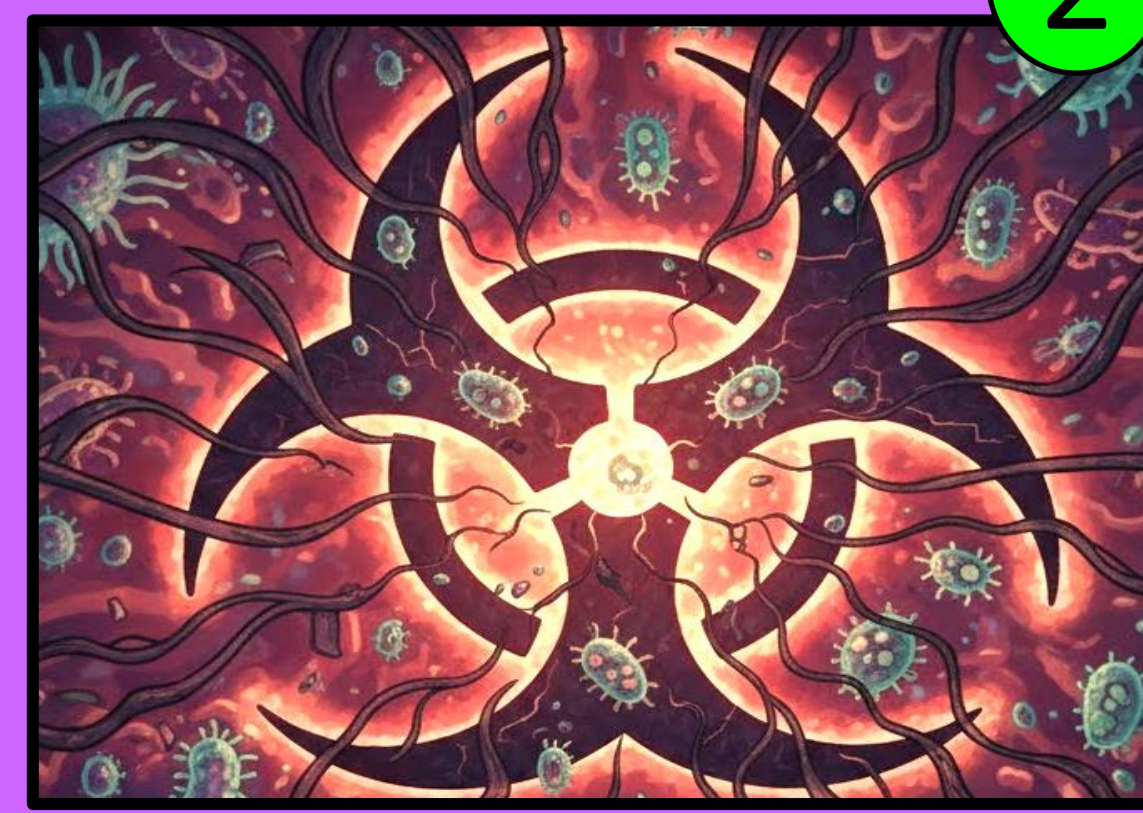

Commensal bacteria. Converts commensals into pathogens.

Blood flow 0

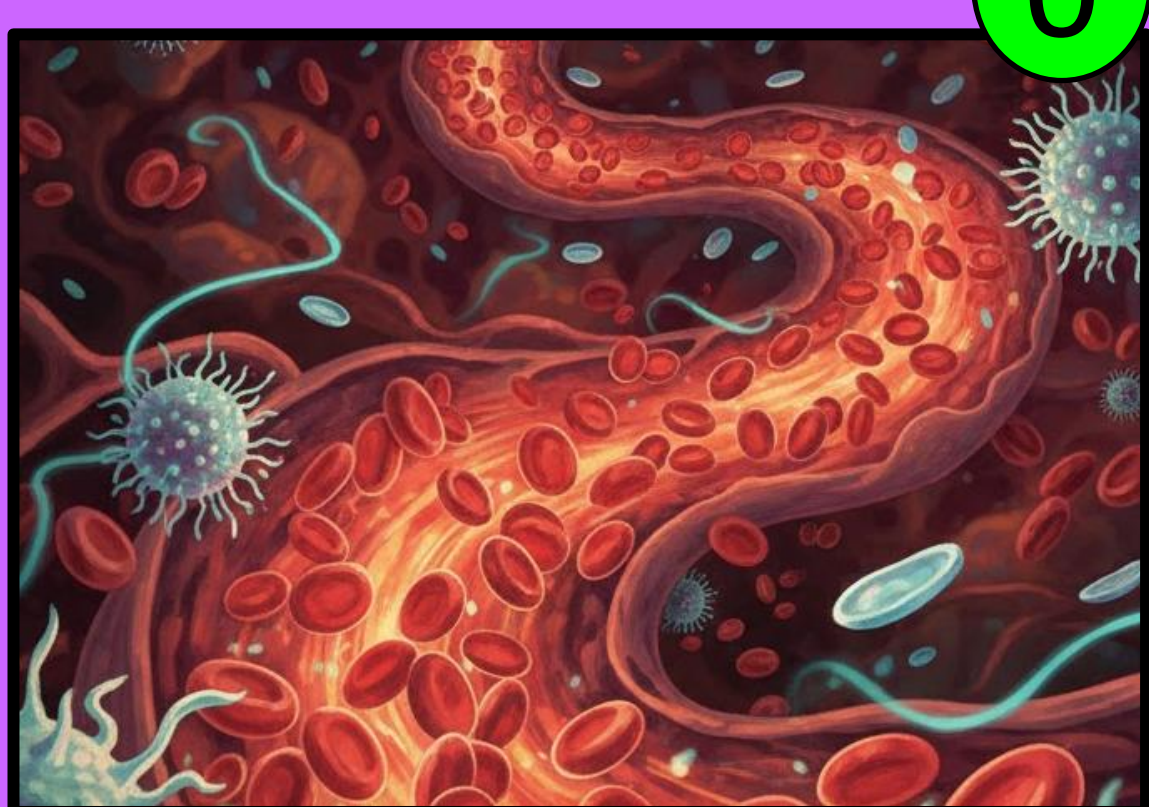

Parasites / vectors. Allows direct entry of blood-borne pathogens.

Conjugation 2

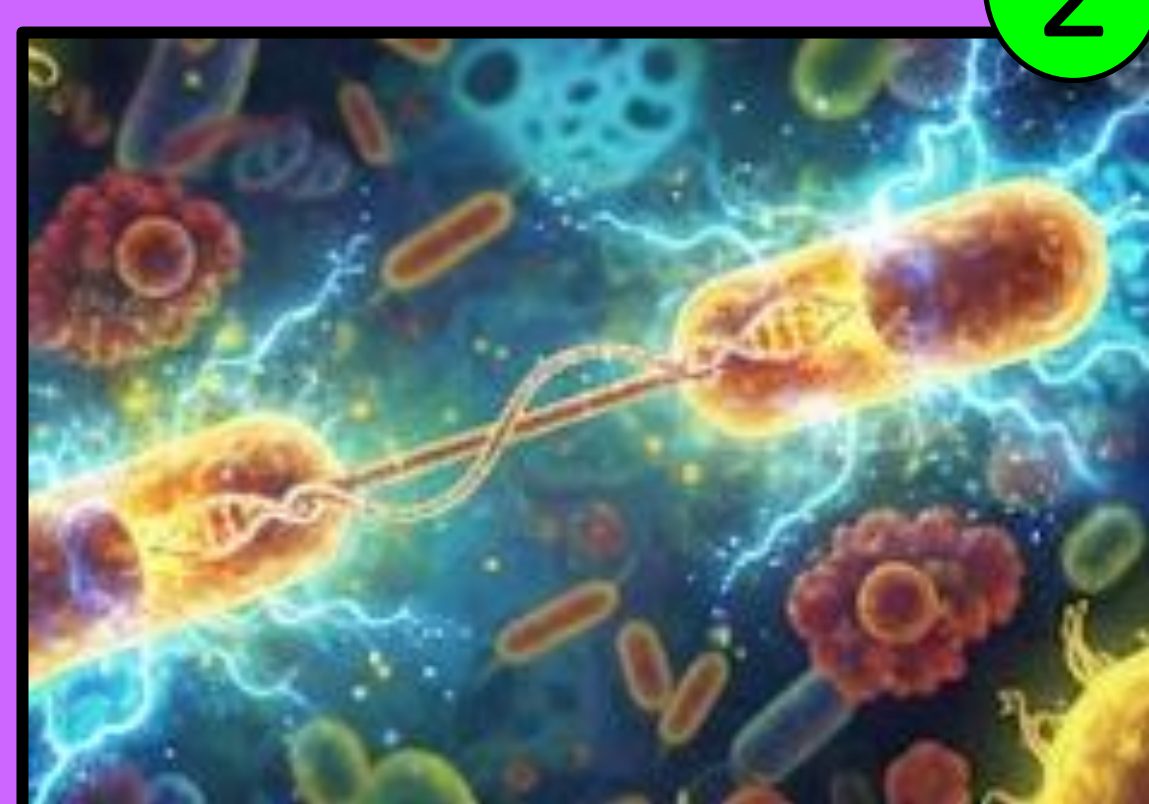

Attacking bacteria. Enhances variability; avoids antibiotics (except autoclave, pasteurization and surfactant).

Contaminated meat 1

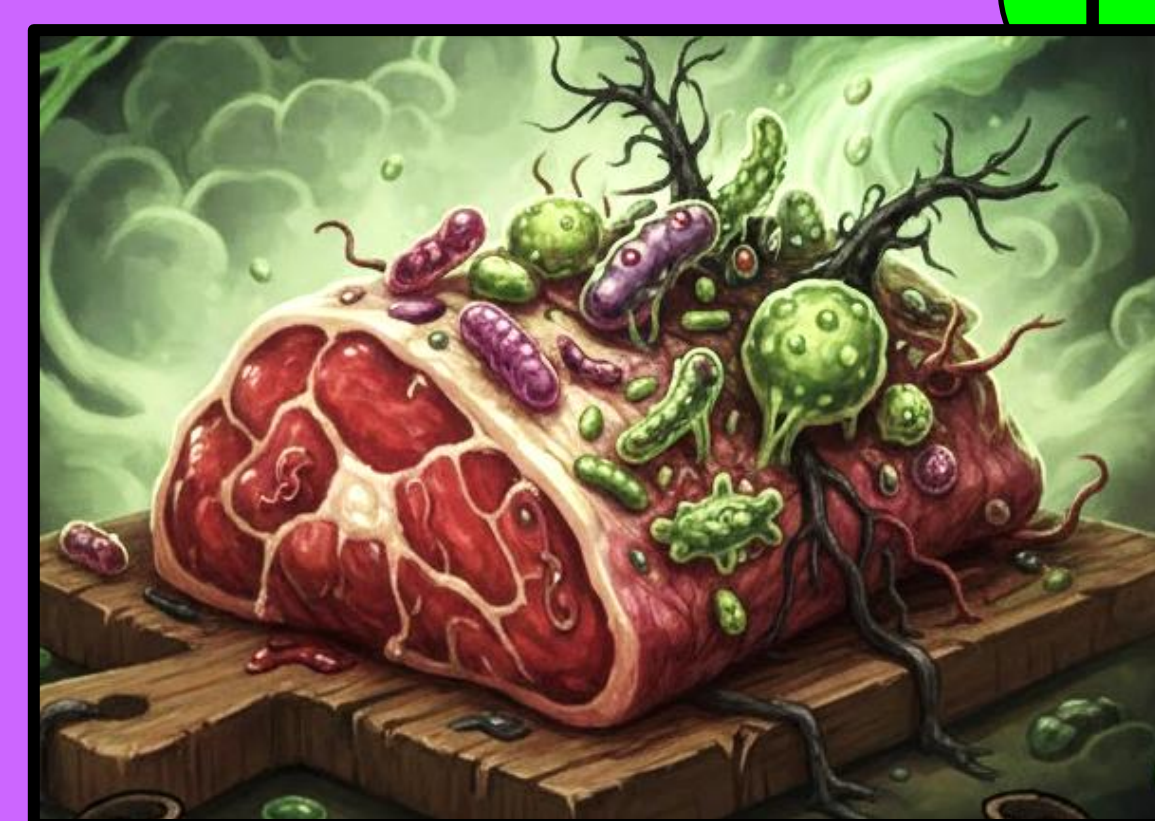

Food-borne pathogens. Carries multiple pathogens; enables transmission.

Deep wounds 2

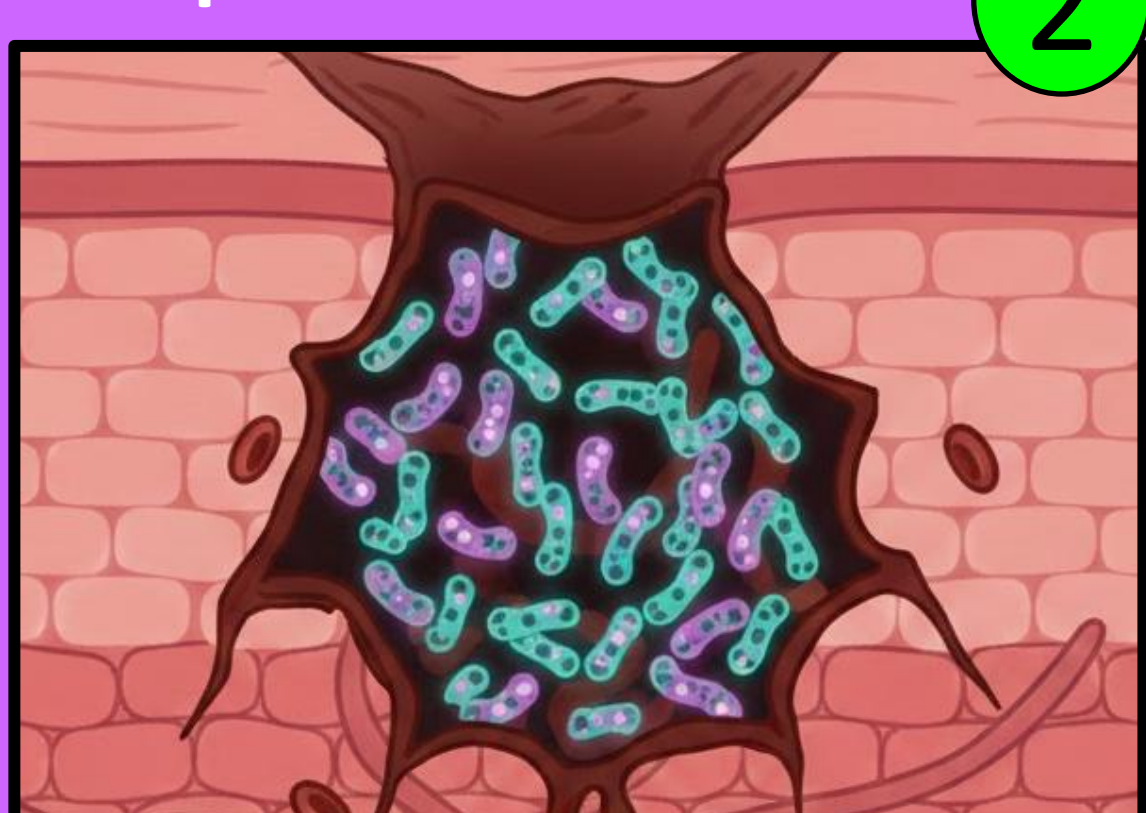

Severe bacterial pathogens. Enables high-damage infections.

Fibrin 2

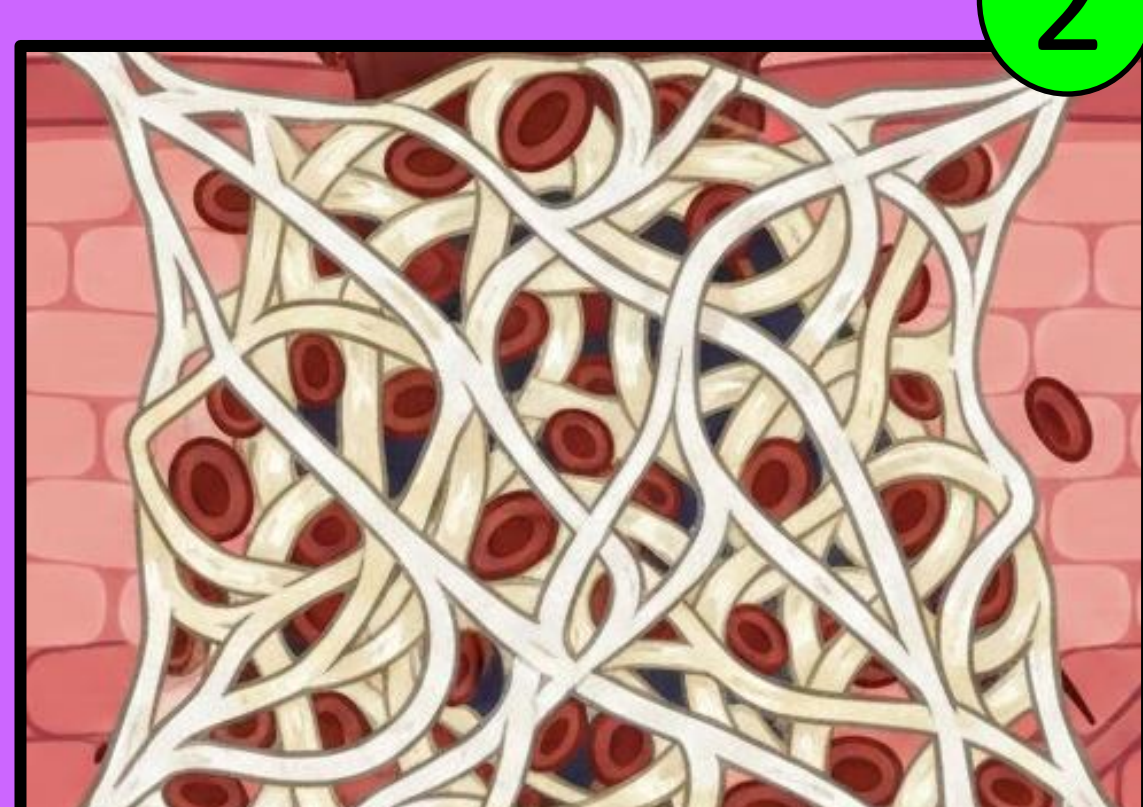

All bacteria. Increases pathogen damage.

Inhalation of spores 0

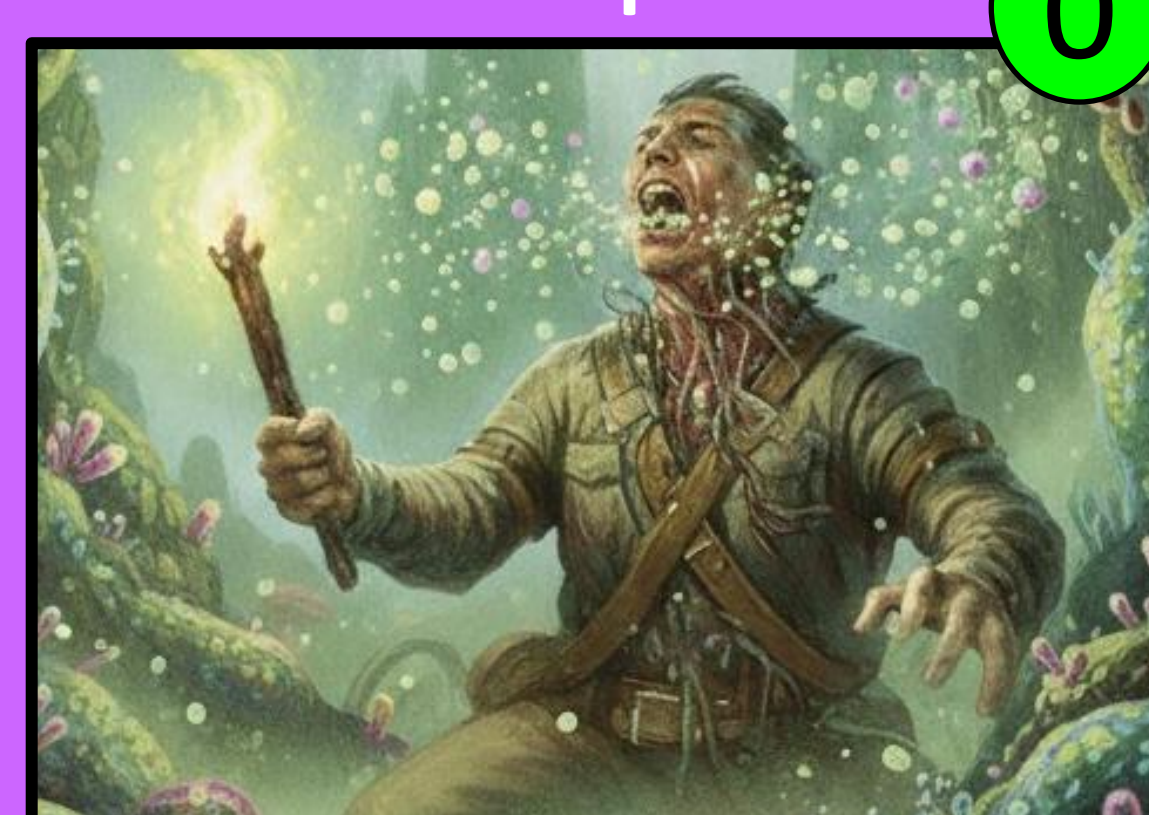

Bacillus anthracis. Enables infection via respiratory route.

Kidney dysfunction 0

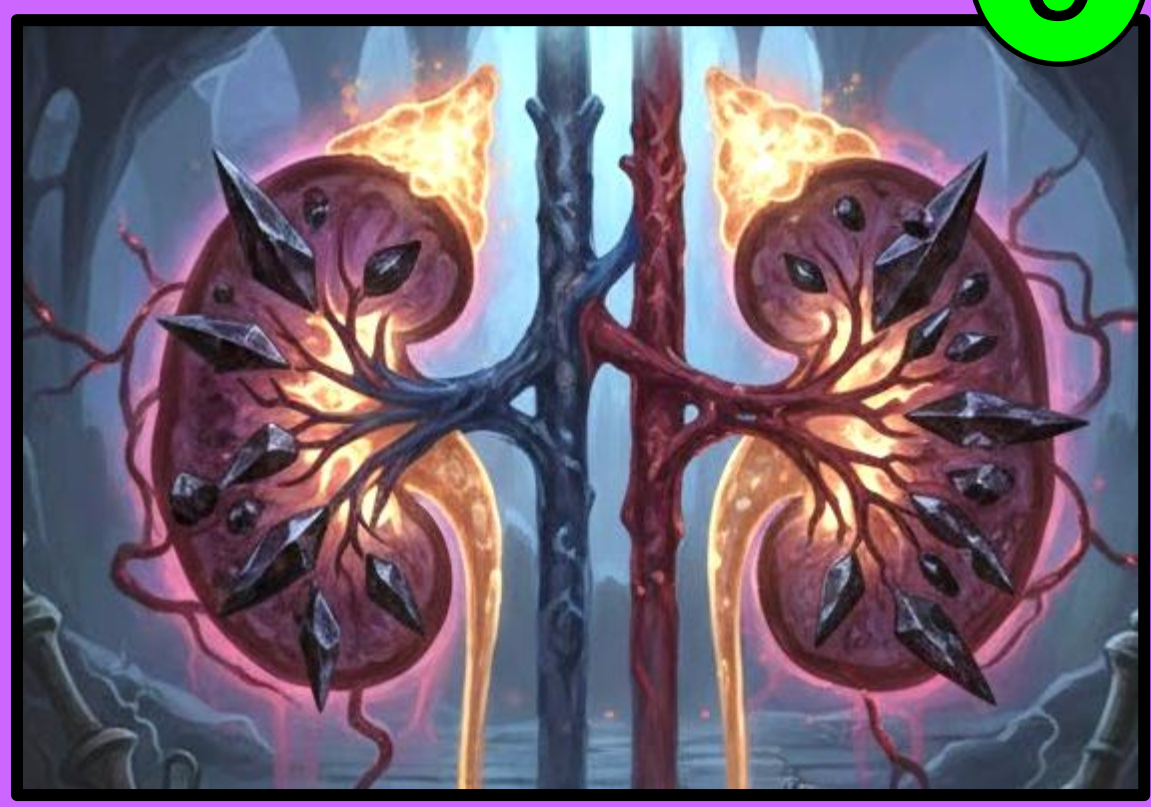

Streptococcus. Induces nephritis if combined.

Lysogenic phage 2

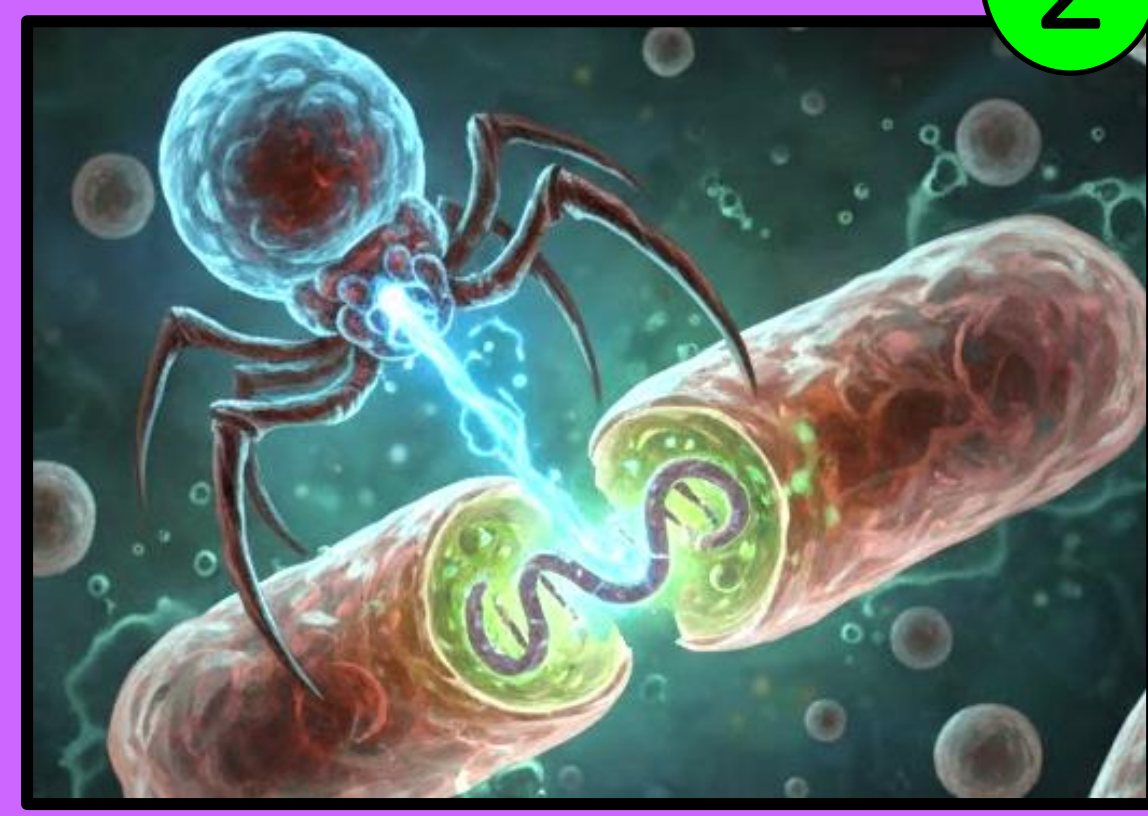

Corynebacterium. Potentiates bacterial attack.

Open wounds 2

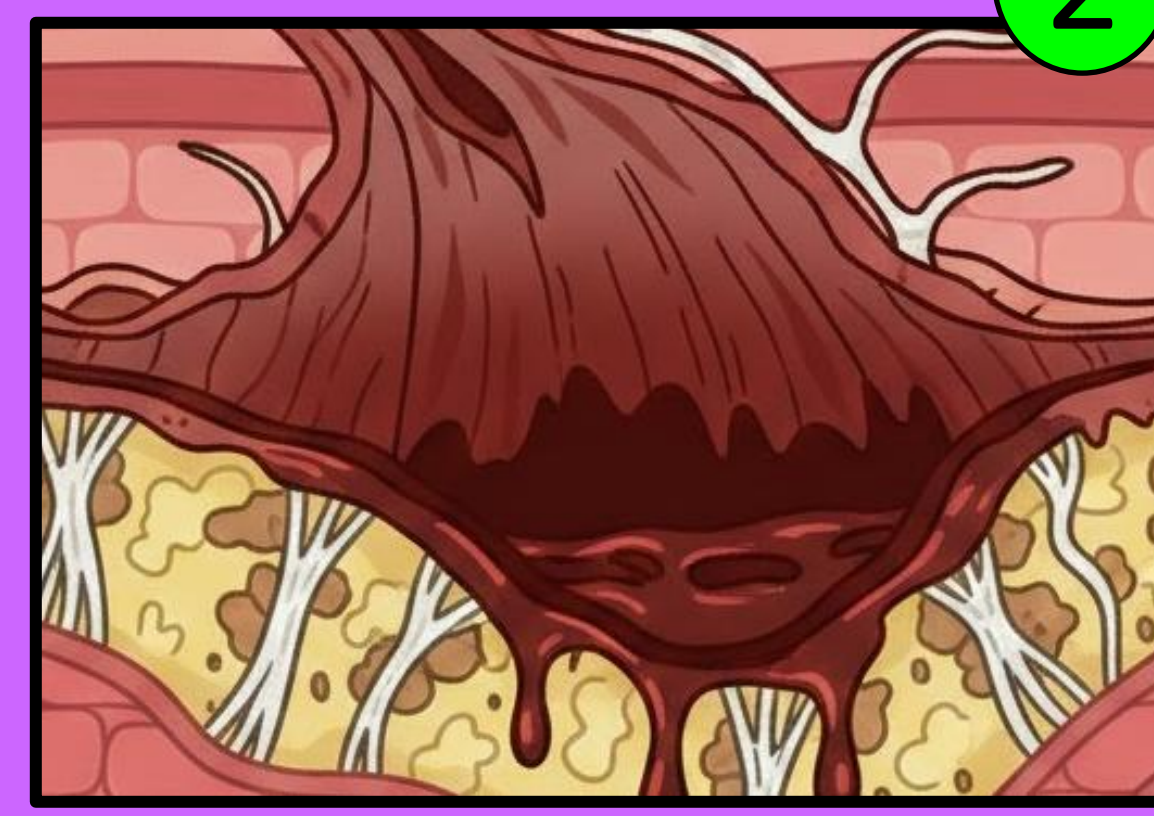

Various bacteria. Enables pathogen entry.

Pediculus humanus 0

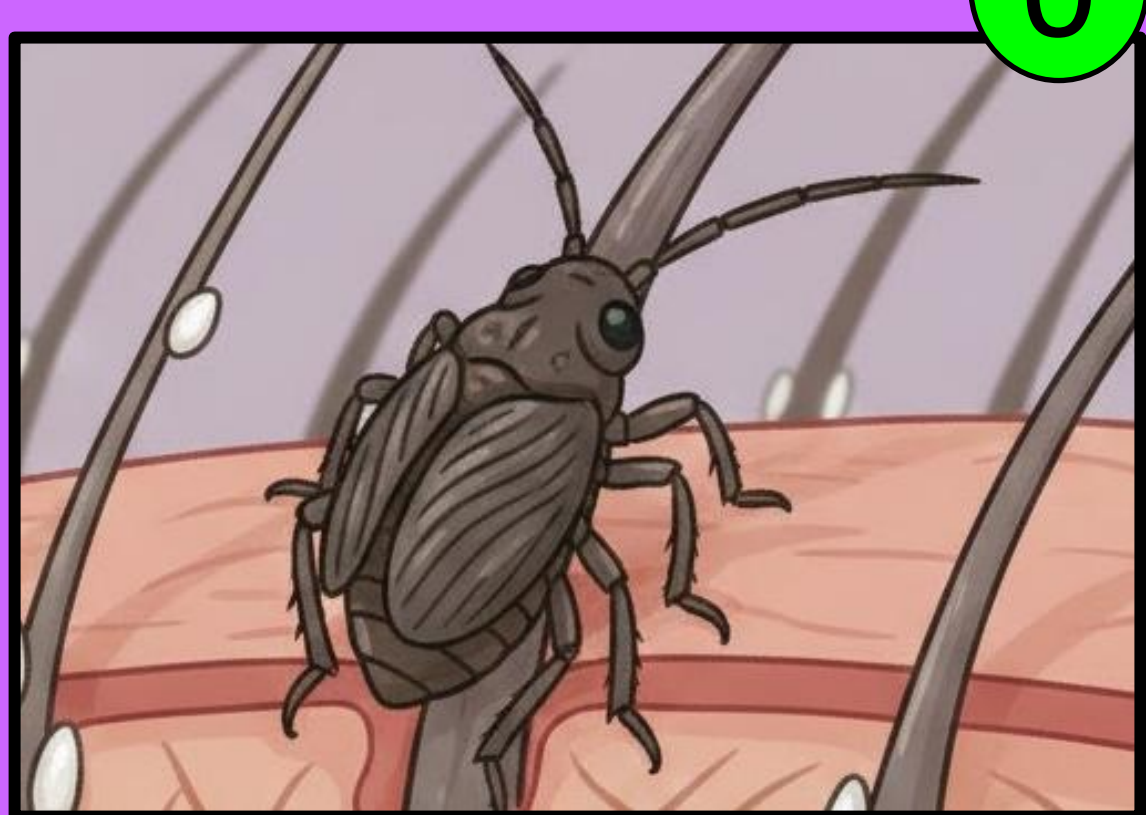

Rickettsia typhi. Facilitates endemic typhus transmission.

Pregnancy 1

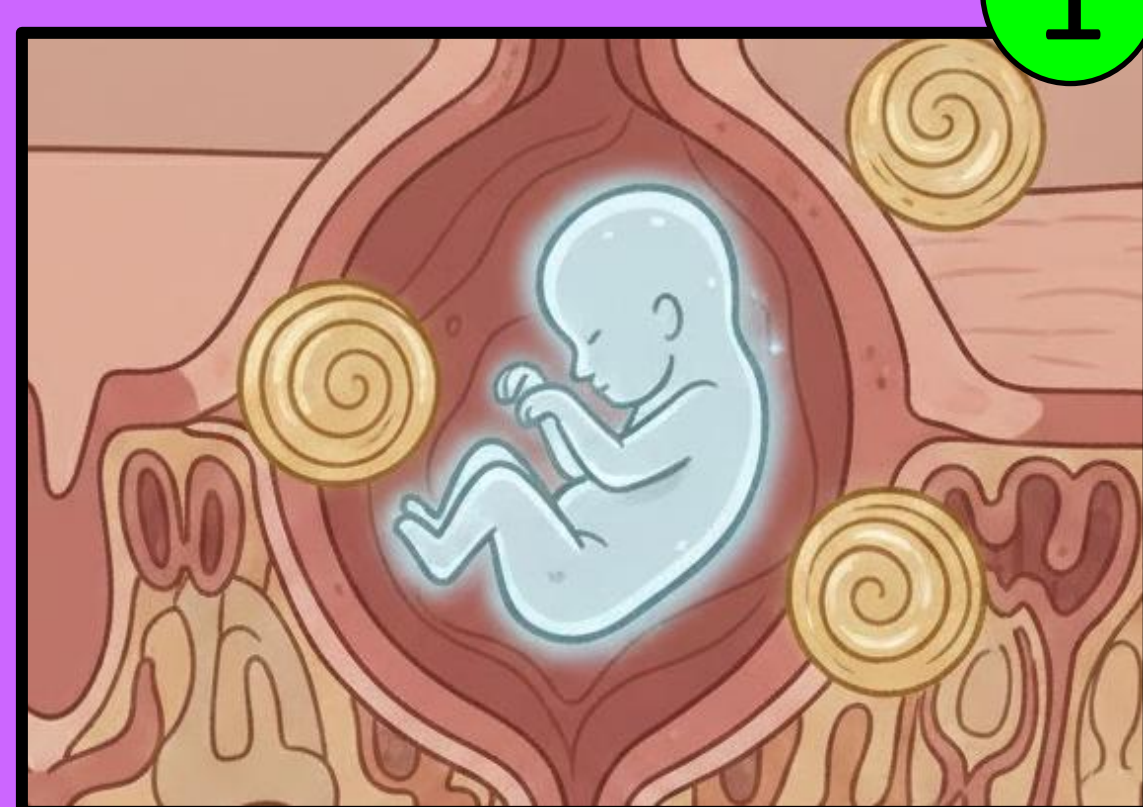

Listeria monocytogenes and *Neisseria meningitidis.* Increases damage when associated.

Rheumatic fever
Valve stenosis 0

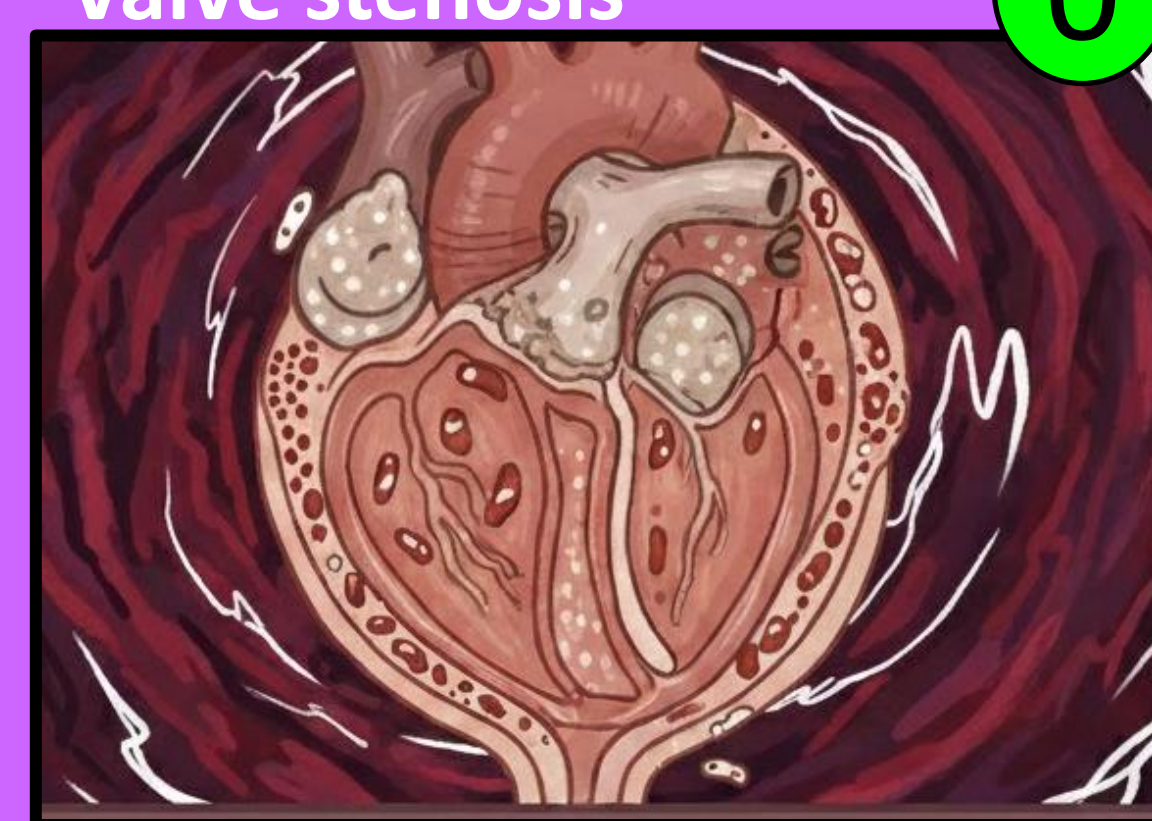

Streptococcus. Causes damage points depending on combination.

Spittle 1

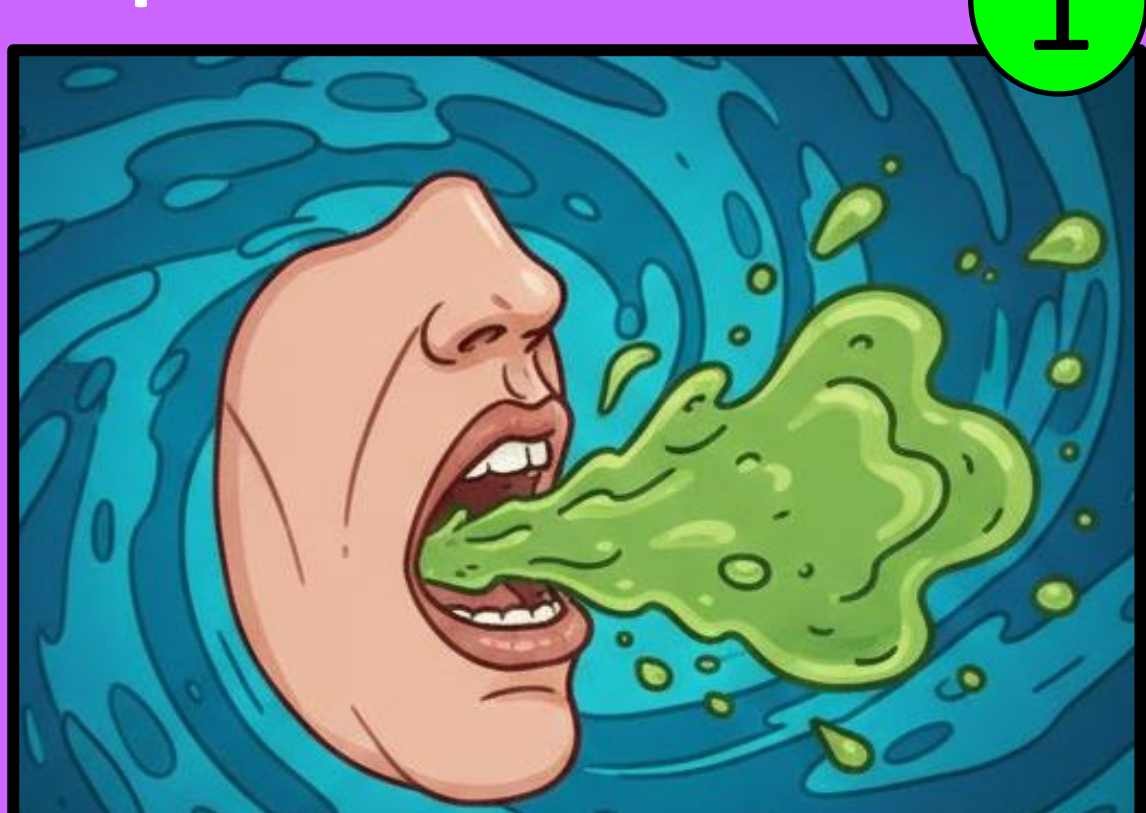

Respiratory pathogens. Enhances pathogen spread.

Spore intake 1

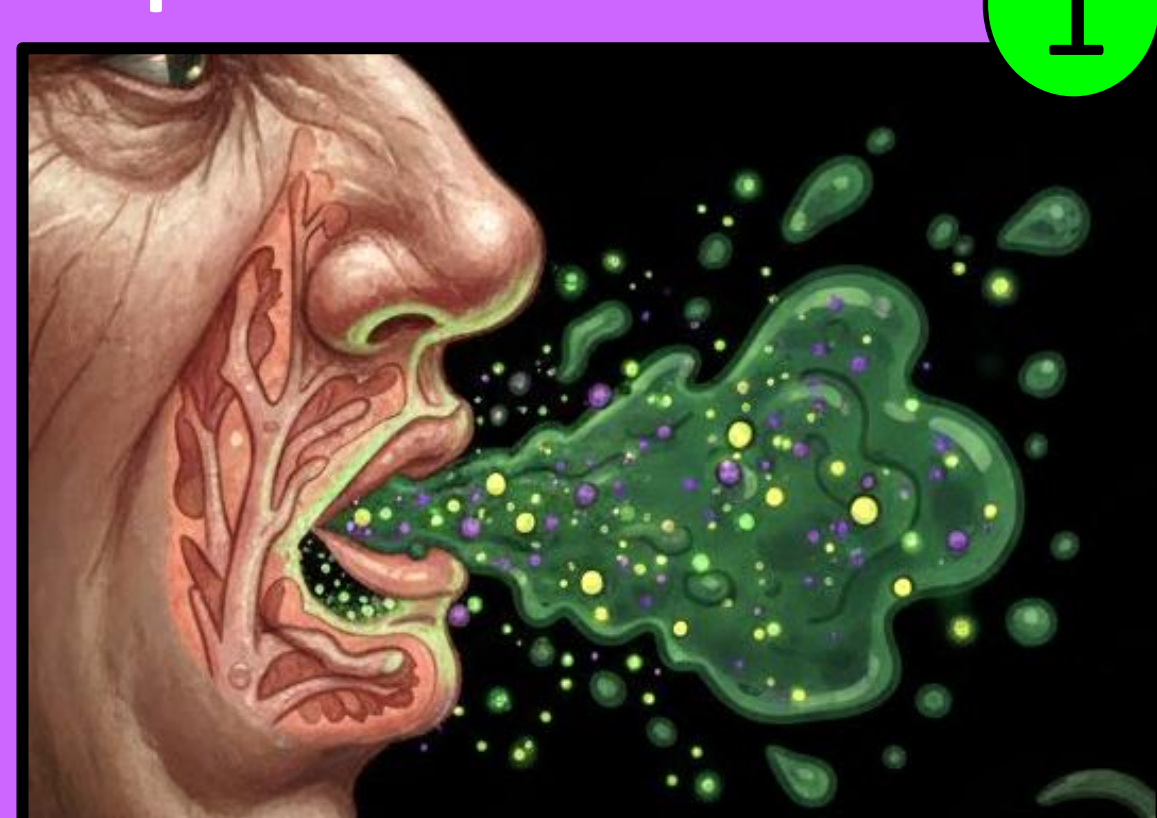

Bacillus anthracis and *Salmonella* spp. Reintroduces bacteria; simulates rapid proliferation.

Star tick bite 0

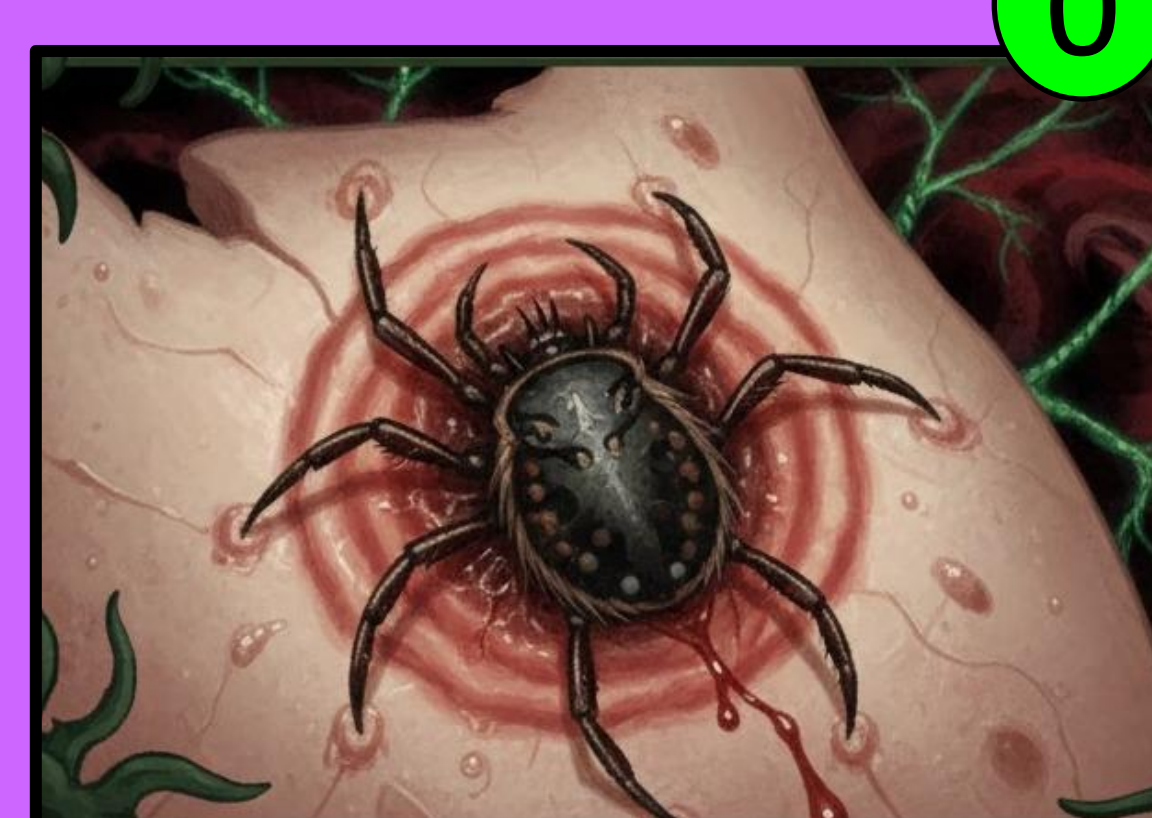

Rickettsia rickettsii. Facilitates Rocky Mountain spotted fever transmission.

Stress 1

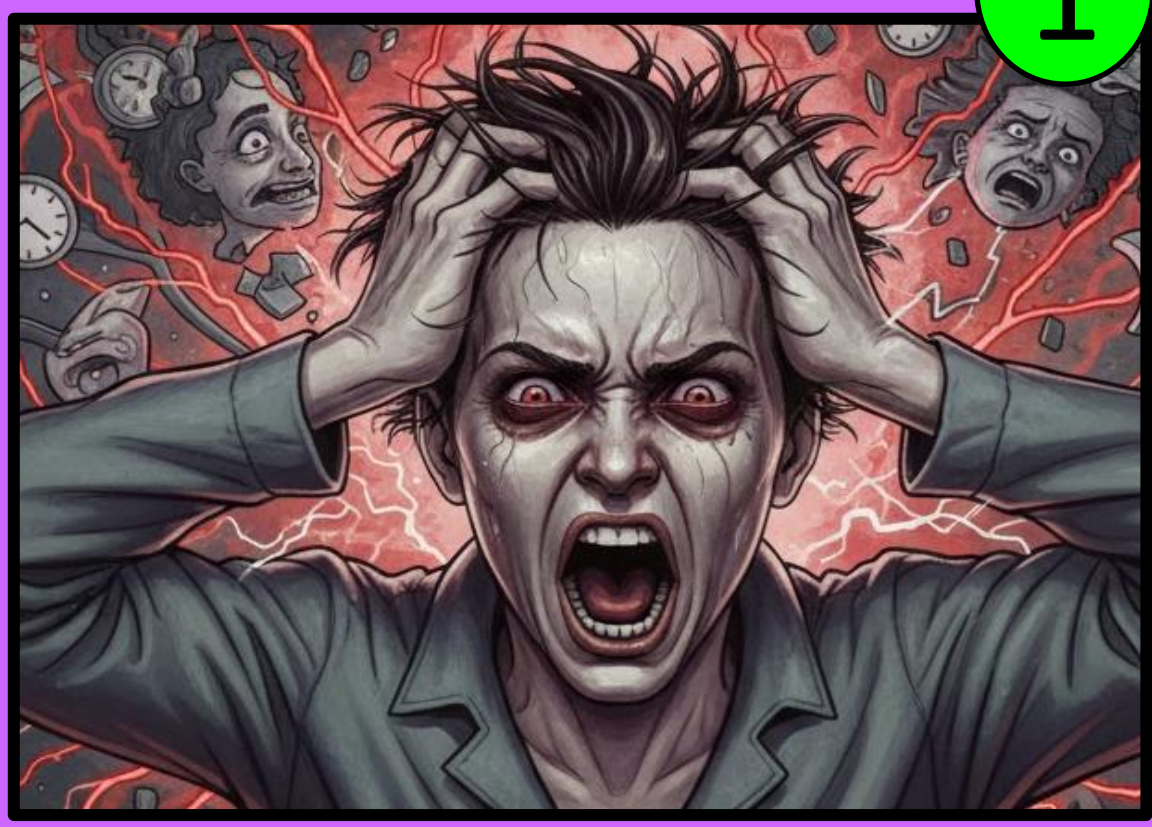

Pathogens. Increases damage caused by pathogens.

Throat infection 1

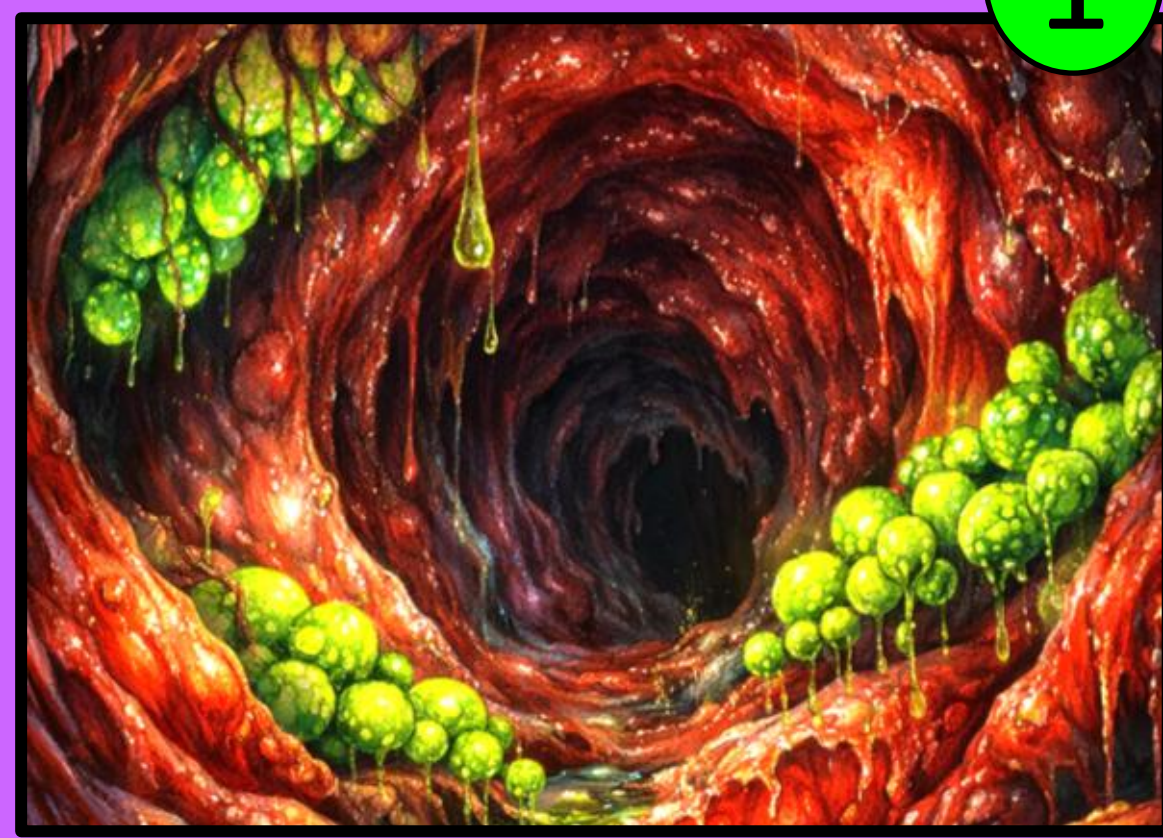

Streptococcus. Boosts attack if combined with relevant cards.

Unpasteurized milk 2

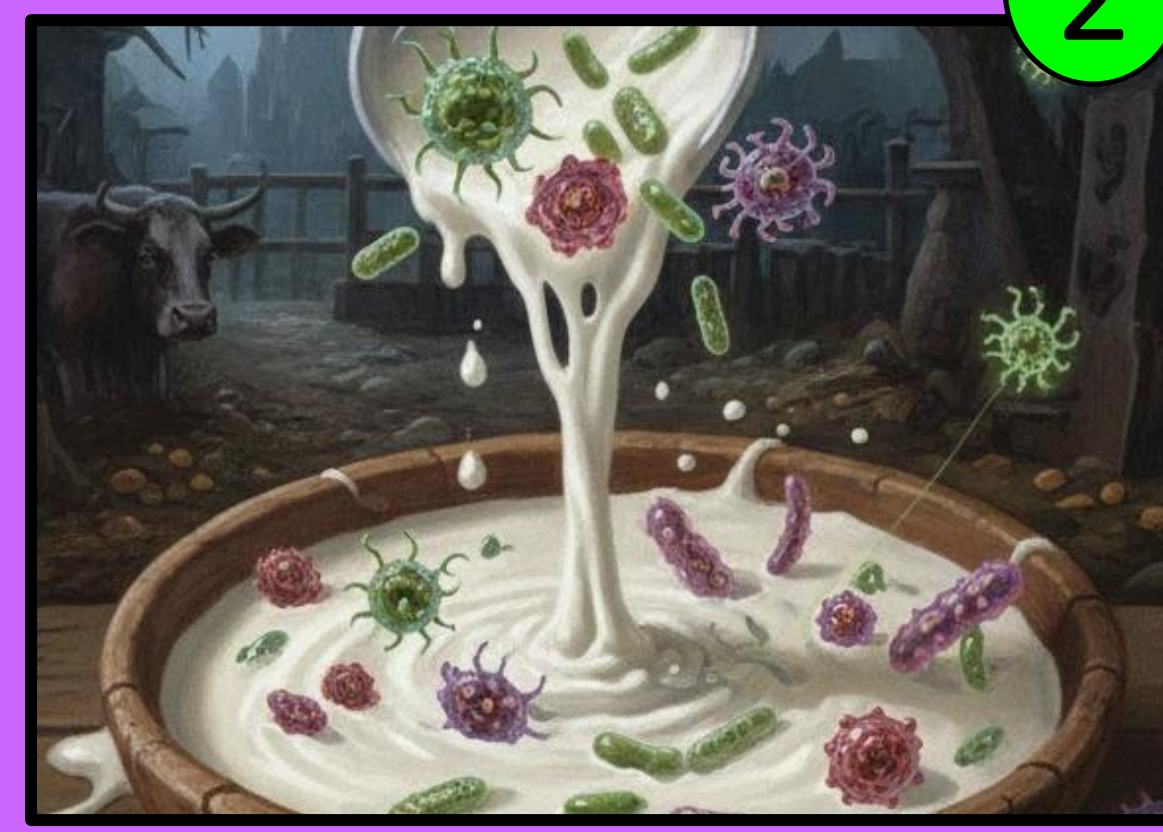

Food-borne bacteria. Reintroduces bacteria up to 3 times; simulates rapid proliferation.

***Xenopsylla cheopis* + Sewer rat** 0

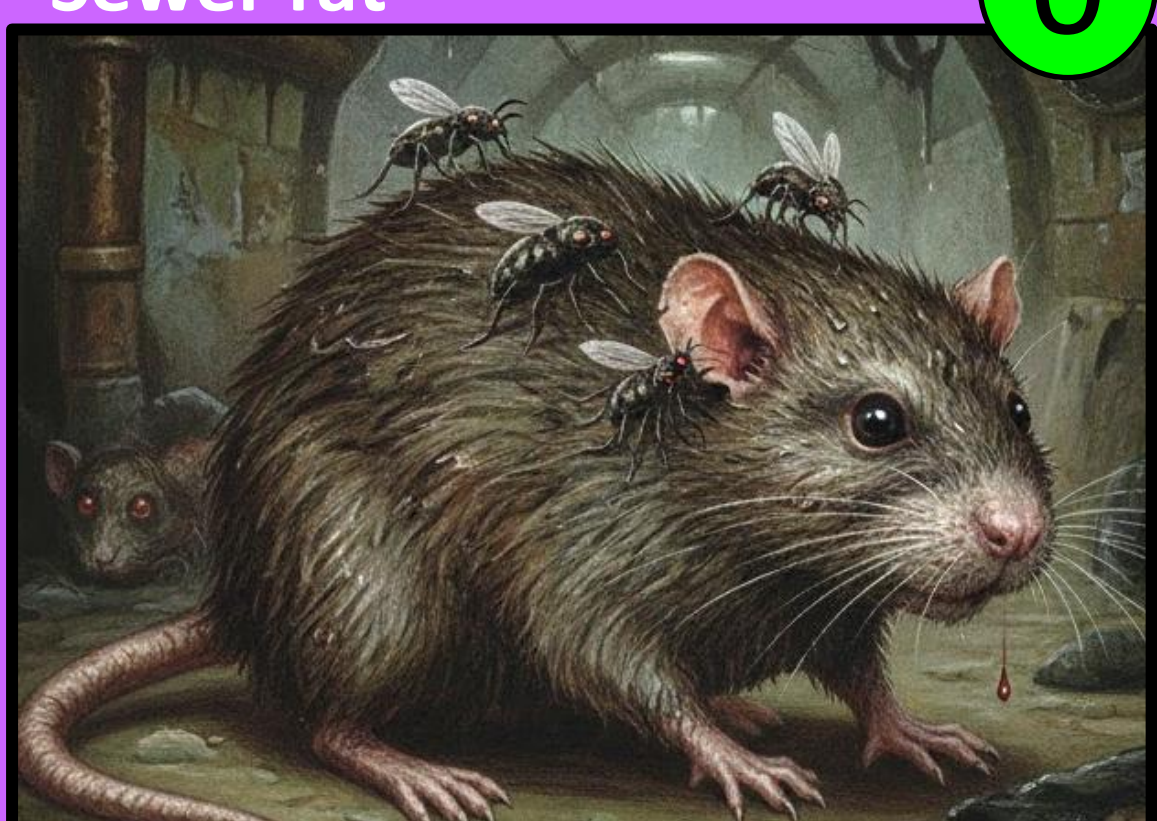

Yersinia pestis. Facilitates bubonic plague transmission.

Game-Based Learning in Microbiology Education: Design and Classroom Use of *MicroBioCombat*

Raul Vinicius Salata Souto[1], Edmara Rocha dos Santos Moreira[1]
and Leandro Marcio Moreira[1,2#]

Supplementary File 2: Scoring table for printing.

Vital Energy Table

MicroBioCombat

Round	Points	Activities	Other observations
1	**20**		
2			
3			
4			
5			
6			
7			
8			
9			
10			
11			
12			
13			
14			
15			

Game-Based Learning in Microbiology Education: Design and Classroom Use of *MicroBioCombat*

Raul Vinicius Salata Souto[1], Edmara Rocha dos Santos Moreira[1] and Leandro Marcio Moreira[1,2#]

Supplementary File 3: Game rules for printing.

MicroBio COMBAT

TEACHER’S GUIDE

Ouro Preto, Minas Gerais, Brazil

TEACHER’S GUIDE
MicroBioCombat

1. About the Game

MicroBioCombat is an educational card game inspired by strategy-based games such as *Magic: The Gathering*™. It was designed to teach microbiology in an engaging and interactive way, especially at the high school level.

The game simulates “biological battles” involving:

- Pathogenic bacteria
- Transmission mechanisms
- Defense and prevention strategies

Through strategic gameplay, students apply microbiology concepts in a playful but structured environment. The game works as a **low-cost virtual laboratory**, allowing teachers to simulate complex biological processes even in schools without laboratory facilities.

2. Learning Objectives

2.1. Cognitive Goals

Students will be able to:

- Identify pathogenic bacteria and their characteristics
- Understand how infections are transmitted
- Connect pathogens to affected organs and symptoms
- Recognize host defense mechanisms
- Understand prevention strategies such as vaccines and hygiene

2.2. Skills Development

Students will:

- Apply scientific concepts to problem-solving situations
- Develop strategic reasoning
- Integrate multiple biological concepts in decision-making

2.3. Social and Emotional Skills

The game promotes:

- Teamwork
- Scientific argumentation
- Leadership and respectful discussion
- Reduced anxiety toward complex scientific topics

3. Guidance for Teachers

3.1. Before Playing

It is recommended to prepare students with:

- A lesson covering:
    - Domain Bacteria / Archaea
    - Bacterial morphology and physiology
    - Commensal vs pathogenic relationships
    - Antibiotic resistance
    - Basic immune system concepts
- A short diagnostic activity to assess prior knowledge

3.2. During the Game

The teacher acts as:

- A scientific mediator
- A facilitator of discussion
- A concept clarifier
- An observer of student reasoning

Encourage students to:

- Justify their moves biologically
- Use correct scientific vocabulary
- Explain why an attack or defense works

This step is essential for transforming the game into meaningful learning.

3.3. After the Game

Recommended activities:

- A post-game diagnostic activity
- Group discussion about strategies used
- Creation of concept maps connecting:
    - Pathogen
    - Transmission route
    - Target organ
    - Defense strategy

4. Game Components

MicroBioCombat includes:

- **81 Cards divided into three categories:**
- pathogenic bacteria (yellow = 26 cards),
- defense strategies (blue = 33 cards)
- mechanisms of conveyance and aiding of pathogens (purple = 22 cards)

4.1. Pathogenic Bacteria Cards (26 cards)

Each card includes:

- Scientific name
- Associated disease
- Biological characteristics
- Energy cost for activation
- Damage potential

4.4. Transmission / Conveyance Cards (22 cards)

Examples:

- Bloodstream
- Deep wound
- Contaminated food
- Anaerobic environment

These cards represent how bacteria reach the host.

4.3. Defense / Protection Cards (33 cards)

Examples:

- Vaccines (e.g., DPT)
- Antibiotics
- Immune cells (neutrophils, macrophages)
- Hygiene measures

- Environmental changes
- Healing ointments

Additional Materials

- 1 die.
- Pen or pencil.
- Vital Energy Table (score sheet) - Available as a separate downloadable file.

5. Game Setup

- Each player (or pair) starts with **20 life points**.
- Each player chooses **7 cards** from any category.
- Remaining cards stay in organized piles.
- The Vital Energy Table must be available to record life points.

The game can be played:

- Individually
- In pairs
- On a table or defined floor space

6. How to Play – Step by Step

6.1. Start of Each Round

1. The player rolls the die.
2. The number rolled equals the available **energy points**.
3. Unused energy points can be saved for future rounds.

6.2. Attack Phase

To attack:

- The player must activate a pathogenic bacteria card.
- In many cases, they must also activate a transmission card.
- The total energy cost must be equal to or lower than available energy.

If successful:

- The opponent loses life points equal to the attack cost.

6.3. Defense Phase

The opponent may:

- Roll the die
- Activate defense cards
- Block the attack if energy is sufficient and the defense is biologically appropriate

If the defense is scientifically coherent and energetically valid:

- The attack is neutralized.

6.4. Card Exchange

At the end of each full round:

- Players may exchange one card
- The returned card goes to the bottom of its original pile

6.5. Winning the Game

The winner is the player (or pair) who reduces the opponent’s life points to **zero**.

7. Vital Energy Table (Scoring System)
Important:
- Record all damage immediately
- Update life points after each attack
- Track stored energy if accumulated

The table helps students:
- Visualize consequences of strategies
- Understand energy cost vs biological impact
- Reflect on decision-making

8. Example Scenarios
✓ Successful Attack
***Staphylococcus aureus* + Bloodstream**
If the player has enough energy:
- The bacterium reaches systemic circulation
- The opponent loses life points
- Unless a valid defense is activated

✓ Successful Defense
***Clostridium tetani* + Deep Wound**
Possible defenses:
- Aerobic environment (oxygen exposure)
- Healing ointment
- DPT vaccine

Students must explain:
- Why tetanus bacteria require anaerobic conditions
- How oxygen or vaccination prevents disease

This discussion is where the strongest learning happens.

9. Educational Impact
MicroBioCombat:
- Functions as a "virtual microbiology lab"
- Encourages critical thinking
- Promotes scientific dialogue
- Works well in low-resource schools
- Increases engagement and motivation

The game transforms abstract microbiology concepts into dynamic, interactive learning experiences.

10. Target Audience
- High school biology classes
- Upper middle school science
- Introductory microbiology courses
